# *Aspergillus hancockii* sp. nov., a biosynthetically talented fungus endemic to southeastern Australian soils

**DOI:** 10.1371/journal.pone.0170254

**Published:** 2017-04-05

**Authors:** John I. Pitt, Lene Lange, Alastair E. Lacey, Daniel Vuong, David J. Midgley, Paul Greenfield, Mark I. Bradbury, Ernest Lacey, Peter K. Busk, Bo Pilgaard, Yit-Heng Chooi, Andrew M. Piggott

**Affiliations:** 1 Commonwealth Scientific and Industrial Research Organisation, North Ryde, Australia; 2 Department of Chemical and Biochemical Engineering, Technical University of Denmark, Lyngby, Denmark; 3 Microbial Screening Technologies, Smithfield, Australia; 4 School of Chemistry and Biochemistry, University of Western Australia, Crawley, Australia; 5 Department of Chemistry and Biomolecular Sciences, Macquarie University, Sydney NSW, Australia; University of Wisconsin Madison, UNITED STATES

## Abstract

*Aspergillus hancockii* sp. nov., classified in *Aspergillus* subgenus *Circumdati* section *Flavi*, was originally isolated from soil in peanut fields near Kumbia, in the South Burnett region of southeast Queensland, Australia, and has since been found occasionally from other substrates and locations in southeast Australia. It is phylogenetically and phenotypically related most closely to *A*. *leporis* States and M. Chr., but differs in conidial colour, other minor features and particularly in metabolite profile. When cultivated on rice as an optimal substrate, *A*. *hancockii* produced an extensive array of 69 secondary metabolites. Eleven of the 15 most abundant secondary metabolites, constituting 90% of the total area under the curve of the HPLC trace of the crude extract, were novel. The genome of *A*. *hancockii*, approximately 40 Mbp, was sequenced and mined for genes encoding carbohydrate degrading enzymes identified the presence of more than 370 genes in 114 gene clusters, demonstrating that *A*. *hancockii* has the capacity to degrade cellulose, hemicellulose, lignin, pectin, starch, chitin, cutin and fructan as nutrient sources. Like most *Aspergillus* species, *A*. *hancockii* exhibited a diverse secondary metabolite gene profile, encoding 26 polyketide synthase, 16 nonribosomal peptide synthase and 15 nonribosomal peptide synthase-like enzymes.

## Introduction

The fungal genus Aspergillus is a very important source of industrial enzymes and metabolic products as well as the major mycotoxins aflatoxin and ochratoxin A. During an ecological survey of *Aspergillus* species in the South Burnett region of Southeast Queensland in 1982, an unusual fungus was isolated from soil that had previously been under peanut cultivation. The isolate was conspicuous because it produced rapidly growing, floccose colonies, with very long conidiophores bearing spherical heads and forming elongate black sclerotia in age. These features were indicative of a species that would be classified currently in the *A*. *alliaceus* clade in *Aspergillus* subgenus *Circumdati* section *Flavi*, near *A*. *leporis*, but distinguished by producing green conidia. It was lost from our collection, but isolated again five years later from the same property. It was not isolated from any other locality in the South Burnett. More than 10 years later, we isolated other strains from a food sample (dried peas) in northwestern Victoria, and subsequently from uncultivated road side soil samples in southern NSW, over 1000 km south of the South Burnett region. It therefore has a widespread but sparse distribution in eastern Australia. Further examination showed that this species produced a unique DNA sequence and an unusually large range of secondary metobiltes, many of them unique. Using the genome sequence, it was also possible to study its production of carbohydrate degrading enzymes and the diversity of its secondary metabolite gene profile. This species is described in this paper as *A*. *hancockii* Pitt sp. nov.

## Materials and methods

### Classical taxonomy

Isolates were grown for 7 days on the standard regimen of Czapek yeast extract agar (CYA), malt extract agar (MEA) and glycerol nitrate agar (G25N) at 25°C, and CYA at 37°C [1], and morphological descriptions were prepared. Capitalised colours mentioned are taken from the Methuen Handbook of Colour [2]. Colonies on CYA and MEA were photographed after growth for 7 days on CYA and MEA, and photomicrographs were prepared from colonies on CYA after 7 days incubation at 25°C. Cultures were freeze dried and accessioned into the FRR collection, the culture collection at CSIRO Agriculture and Food, North Ryde, NSW.

### Nomenclature

The electronic version of this article in Portable Document Format (PDF) in a work with an ISSN or ISBN number will represent a published work according to the International Code of Nomenclature for algae, fungi and plants, and hence the new name contained in the electronic publication of a PLoS ONE article are effectively published under that Code.

The new names contained in this work has been submitted to MycoBank from where it will be made available to the Global Names Index. The unique MycoBank number can be resolved and the associated information viewed through any standard web browser by appending the MycoBank number contained in this publication to the prefix http://www.mycobank.org/MB/. The online version of this work is archived and available from the following digital repositories: PubMed Central, LOCKSS and CSIRO ePublish.

### Genome sequencing, gene calling and bioinformatics

*Aspergillus hancockii* FRR 3425 was grown in liquid malt extract medium for 7 days with gentle shaking at ~40 rpm at room temperature. DNA from mycelium (~200 mg) was extracted using the PowerSoil® DNA Isolation Kit (Mo Bio Laboratories, Carlsbad, CA). For genomic sequencing, Nextera XT libraries were prepared using Illumina kits (Sample Prep Kit, Illumina, San Diego, CA), then subjected to quality control using a bioanalyser before whole genome sequencing was carried out using 100 bp paired-end Illumina HiSeq 2000 with an insert size of 300bp. The resultant sequence data were corrected using Blue 1.0.1 [3] then assembled with Velvet 1.2.10 [4], using a kmer length of 57 and a minimum cutoff coverage of 11. The mitochondrial genome was removed from the nuclear genome and is available via the CSIRO Data Access Portal https://data.csiro.au/dap/landingpage?pid=csiro:18690. For the nuclear genome, contigs with a length greater than 1000 bp were analysed and are represented by the accessioned set as a GenBank BioProject under the accession PRJNA328536; the entire nuclear genome, including the small contigs and raw sequencing reads (fastq), are available via the CSIRO Data Access Portal (https://data.csiro.au/dap/search?tn=Mycology).

Genes were predicted and translated with AUGUSTUS [5], using *Aspergillus oryzae* as a model fungus, using the default parameters (augustus–species = aspergillus_oryzae_queryfilename > output.gff). Identified genes were examined using the dbCAN pipeline [6] to detect genes encoding carbohydrate-active enzymes. dbCAN results, the unparsed output from AUGUSTUS, amino acid sequences for genes, exonic and complete coding sequences are all available for download at https://data.csiro.au/search?tn=Mycolocy. Phylogenetic comparisons were made from extracted single gene sequences for β-tubulin and calmodulin, plus the Internal Transcribed Spacer region (Table 1). These sequences were compared with relevant species from *Aspergillus* section *Circumdati* using the Maximum Likelihood technique and the Tamura-Nei model [7]. Bootstrap values were calculated from 1000 replicates. All positions containing gaps and missing data were eliminated. Gene alignment and tree construction were performed using MEGA7 [8].

**Table 1:** Strains used in phylogenetic analyses.

Number	Species	Source	GenBank accession number		
			BT	Calmodulin	ITS
FRR 3425	*A*. *hancockii*	Soil, Qld	MBFL01001228: 26544–27352	MBFL01000377: 5490–6238	KX858324
NRRL 359	*A*. *aculeatus*	Unknown	EF661106.1	EF661146.1	AJ280004.1
NRRL 315	*A*. *alliaceus*	Beetle,Washington DC	EF661465.1	EF661534.1	EF661551.1
NRRL 25528	*A*. *caelatus*	Soil, GA	EF661471.1	EF661522.1	AF004930.1
NRRL 2254	*A*. *clavatus*	Dung, Br. Guiana	EF669809.1	EF669885.1	EF669951.1
NRRL 1957	*A*. *flavus*	Cellophane, S. Pacific	AY017536.1	AY017583.1	AF027863.1
NRRL 164	*A*. *fumigatus*	Soil, Germany	EF669792.1	EF669861.1	EF669932.1
NRRL 3648	*A*. *lanosus*	Soil India	EF661468.1	EF661539.1	EF661553.1
NRRL 3216	*A*. *leporis*	Dung. WY	EF661500.1	EF661542.1	AF104443.1
NRRL 3	*A*. *niger*	USA	EF661088.1	EF661154.1	EF661186.1
NRRL 3161	*A*. *nomius*	Cycad, Guam	EF661494.1	EF661531.1	AF338642.1
NRRL 502	*A*. *parasiticus*	Insect, HI	AY017537.1	AY017584.1	EF661546.1
NRRL 425	*A*. *tamarii*	France	EF661475.1	EF661525.1	EF661558.1
NRRL 3174	*A*. *westerdijkiae*	Sorgum, S. Africa	EF661329.1	EF661360.1	EF661428.1

Secondary metabolite biosynthetic gene clusters (BGCs) were predicted using AntiSMASH 3.0 (https://antismash.secondarymetabolites.org/) [9] using standard parameters for eukaryotes. AntiSMASH 3.0 was integrated with the MIBiG (Minimal Information about a Biosynthetic Gene cluster) database [10] which automatically detects the presence of homologous gene clusters. The conserved functional domains of polyketide synthases (PKSs) and nonribosomal peptide synthases (NRPSs) as predicted by AntiSMASH 3.0 were further confirmed by NCBI Conserved Domain Search and EBI InterProScan.

### Enzyme profiling

*Aspergillus hancockii* FRR 3425, grown on potato dextrose agar [1] was used to inoculate liquid wheat bran medium [5 mL, 5% wheat bran (Finax AB, Helsingborg, Denmark) in deionised water]. The culture was incubated at 20°C and 175 rpm on an orbital shaker for 7 days, then the supernatant was harvested by centrifugation. Eleven AZurine Cross-Linked (AZCL) substrates (Megazyme International, Bray, Ireland, Table 2) were used for testing the enzyme activity profile of the supernatant of *A*. *hancockii*. Supernatants (15 μL, undiluted) were added to triplicate wells in Petri dishes containing the different AZCL substrates, distributed evenly on agarose plates and incubated at 30°C for 24 h according to the manufacturer’s protocol. Negative controls, made from uninoculated wheat bran medium, were included. Enzyme activity was confirmed by a blue halo around the sample well, indicating the presence of active enzymes which can break down the specific AZCL substrate, thereby releasing the blue, soluble dye. Measurable blue halos ranged from 0.5 mm to 12 mm radius, providing a sensitive, semi-quantitative assay.

**Table 2:** Enzyme activity profile of liquid culture of *Aspergillus hancockii* FRR 3425^a^.

AZCL Substrate	Enzyme activity	Average zone radius (mm)
Amylose	α-Amylase	2
β-Glucan	β-Glucanase	3
HE-Cellulose	Endo-1,4-β-glucanase	2
Galactomannan	Endo-1,4-β-Mannanase	1
Xyloglucan	Xyloglucanase	1
Xylan	Endo-1,4-β-xylanase	5
Arabinoxylan	Arabinoxylanase	6
Curdlan and Pachyman	Endo-1,3-β-glucanase	0
Galactan	Endo-1,4-β-galactanase	0
De-branched arabinan	Endoarabinanase	0
Rhamnogalacturonan I	Rhamnogalacturonase	0

^a^ Measured on Megazyme azur-linked substrates (AZCL). Zone radius is average of 3 measurements (mm).

A more thorough investigation of the potential ability of *A*. *hancockii* to produce a spectrum of enzymes was undertaken using a new sequence analysis method, peptide pattern recognition, PPR [11]. The algorithm in PPR searches sequences to identify short, highly conserved peptide motifs that enable classification of enzymes to a subfamily level [12]. A series of validation experiments showed that the peptide pattern recognition subfamily grouping correlates to function [12]. This non-alignment-based sequence analysis method produces lists of peptide patterns that enable another PPR program designated HotPep [13] to mine entire genomes efficiently for all types of carbohydrate active enzymes, and can also predict the protein subfamily to which an enzyme belongs. As each protein family covers several enzyme functions, PPR analysis has the significant advantage that it can predict the function of an enzyme directly from its sequence.

### Culture optimisation

Culturing of *Aspergillus hancockii* for analytical chemical profiling was undertaken on a range of liquid, agar and grain based media. Cultures were sampled (1 g) and extracted with methanol (2 mL) for 1 h on a wrist shaker, centrifuged (15,700 × *g* for 3 min) and analysed by HPLC. The major metabolites were analysed using our in-house database, COMET, of HPLC-diode array detector (DAD) traces from >5000 fungal species [14]. Metabolites not previously observed were accessioned and targeted for preparative cultivation, purification, characterisation and structure elucidation.

Optimisation of *A*. *hancockii* cultures for metabolite production was undertaken on a range of agar- and grain-based media. The agars, Czapek-Dox agar (CZA), malt extract agar (MEA), yeast extract sucrose agar (YES), and casein glycerol Agar (CGA), were prepared according to the recipes in (Table A in S1 File). Hydrated grains, barley, rice (jasmine and basmati) and cracked wheat [grain (50 g) with water (30 mL) in a 250 mL flask] were sterilised (120°C for 40 min). The agars and grains, inoculated with a suspension of fungal spores, were incubated at 24°C for 14 days. Cultures were sampled (1 g), extracted with methanol (2 mL) for 1 h on a wrist shaker, centrifuged (15,700 × *g* for 3 min) and analysed by HPLC (Figures A and B in S1 File). The HPLC traces were accessioned into COMET, and the major metabolites were analysed by retention time and UV-vis spectral fit.

### Preparative cultivation of *Aspergillus hancockii*

Based on results from the previous section, rice was hydrated and sterilised in 40 Erlenmeyer flasks (250 mL, each containing 80 g of rice plus water), inoculated with a spore suspension grown on MEA for 7 days and incubated at 24°C for 21 days, by which time the culture had reached maximal metabolite productivity. The cultures were then pooled into a 5 L Erlenmeyer flask for processing.

### Extraction and isolation of metabolites from *Aspergillus hancockii*

The combined cultures (3.1 kg) were extracted with acetone (2 × 4.5 L) overnight on a rotatory platform (125 rpm) then concentrated *in vacuo* to an aqueous residue (750 mL; 57.3 g). The residue was partitioned over ethyl acetate (2 × 2.0 L) to provide a crude organic extract (15.3 g on freeze drying) and the remaining aqueous residue (42 g). A portion (5 g) of the aqueous residue was suspended in 50% MeOH/H_2_O containing 0.1% trifluoroacetic acid (TFA) and centrifuged (1,800 × *g*) to pellet insoluble material. The supernatant was separated by preparative HPLC (Hypersil C_18_, isocratic 45% MeCN/H_2_O containing 0.1% TFA, 60 mL/min) to yield dehydroterrestric acid (*t*_R_ 13.0 min, 10.6 mg).

The organic extract was redissolved in 10% H_2_O/MeOH (500 mL) then partitioned over hexane (2 × 1 L) to remove the lipids, yielding an enriched extract containing the bulk of the nonpolar secondary metabolites (6.7 g). The enriched extract was adsorbed onto silica gel, which was dry-loaded onto a silica gel column (120 g, 300 × 50 mm, Davisil, Grace Discovery, Epping, Vic, Australia). The column was washed once with hexane (250 mL), then eluted with 50% hexane/CHCl_3_ (250 mL) and CHCl_3_ (250 mL), followed by a stepwise gradient of 1, 2, 4, 8, 16, 32, 64 and 100% MeOH/CHCl_3_ (250 mL each step), to yield 10 fractions (Fr 1–10). The fractions were sampled and analysed by C_18_ analytical HPLC.

The CHCl_3_ fraction (Fr 2, 1.5 g) was dissolved in 50% MeOH/DMSO and chromatographed by preparative HPLC (Hypersil C_18_, stepwise gradient 58-75-100% MeCN/H_2_O containing 0.01% TFA, 60 mL/min) to yield speradine F (*t*_R_ 12.2 min, 22.5 mg), hancockiamide analogue (*t*_R_ 15.4 min, 28.3 mg), hancockiamide B (*t*_R_ 19.3 min, 24.9 mg), hancockiamide F (*t*_R_ 24.4 min, 6.6 mg), hancockiamide C (*t*_R_ 21.2 min, 160.2 mg), onychocin A (*t*_R_ 25.5 min, 50.5 mg), onychocin B (*t*_R_ 26.9 min, 239.8 mg), and fumitremorgin A (*t*_R_ 32.3 min, 38.3 mg).

The 1% MeOH/CHCl_3_ fraction (Fr 3, 0.4 g) was purified by preparative HPLC (Hypersil C_18_, isocratic 35% MeCN/H_2_O containing 0.01% TFA, 60 mL/min) to provide a single major metabolite, 7-hydroxytrichothecolon (*t*_R_ 6.8 min, 43.3 mg).

The 2% MeOH/CHCl_3_ fraction (Fr 4, 0.7 g) eluted the dominant hancockiamide, which was resolved on preparative HPLC (Hypersil C_18_, isocratic 35% MeCN/H_2_O containing 0.01% TFA, 60 mL/min) to yield hancockiamide A (*t*_R_ 6.8 min, 267 mg). Re-chromatography (Alltima C_18_, isocratic 10% MeCN/H_2_O containing 0.01% TFA, 10 mL/min) provided a minor analogue, hancockiamide E (*t*_R_ 22.5 min, 2.0 mg).

The most polar metabolites eluted with 16–100% MeOH/CHCl_3_ (Fr 8–10), which were combined, dried *in vacuo*, resuspended in MeOH (10 mL) and the insoluble material pelleted by centrifugation (1,800 × *g*). The supernatant was purified by preparative HPLC (Hypersil C_18_, isocratic 40% MeCN/H_2_O containing 0.01% TFA, 60 mL/min) to yield an enriched mixture of two components, which were resolved by Sephadex LH-20 chromatography (1 × 30 cm glass column) using MeOH to yield hancockiamide D (68.0 mg) and kojic acid (29.8 mg).

A full isolation scheme, ^1^H and ^13^C NMR spectra and tabulated 2D NMR data for all pure compounds are provided in S1 File.

## Results and discussion

### Taxonomy

Morphologically, the new species *Aspergillus hancockii* shows features characteristic of *Aspergillus* subgenus *Circumdati* section *Flavi*. Within that section, it shows similarity with members of the *A*. *alliaceus* clade, a group of uncommonly encountered, fast growing, loosely textured, lightly sporing species [15]. However, it is readily distinguished from other species in that clade by the production of dull green conidia.

**Fig 1:**
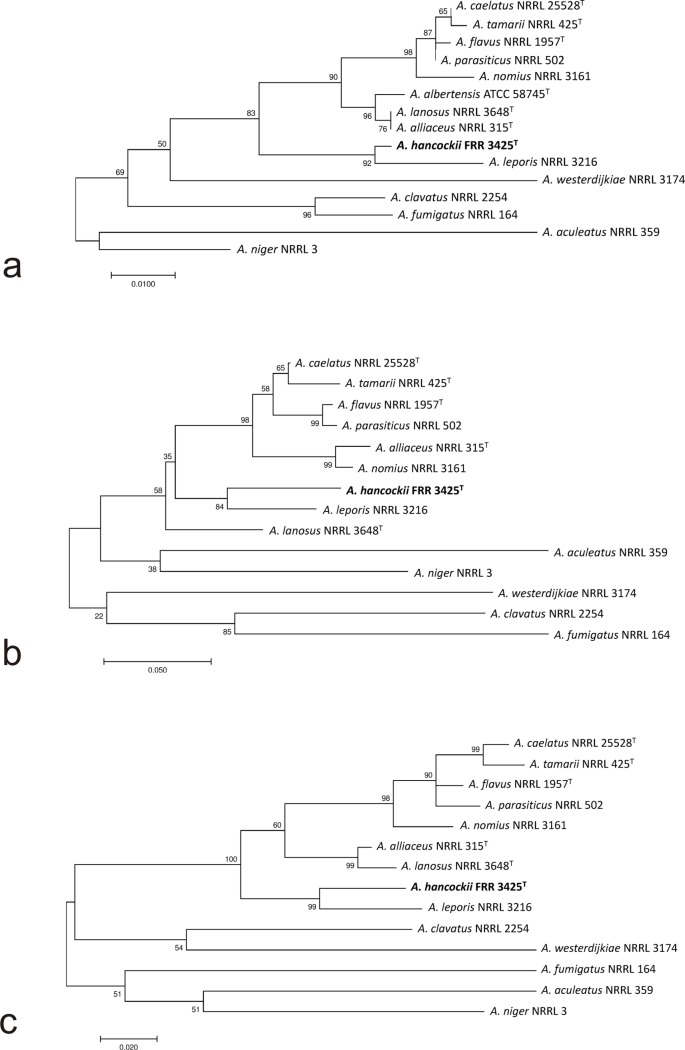
Maximum likelihood trees of (a) the internal transcribed spacer region; (b) β-tubulin gene; and (c) calmodulin gene from *Aspergillus hancockii* and related species. Trees with the highest log likelihood are shown. Trees are drawn to scale, with branch lengths measured as the number of substitutions per site. Numbers on branches indicate bootstrap support (1000 replicates).

Phylogenetically, trees generated from ITS, β-tubulin and calmodulin genes (Fig 1) all indicate that *A*. *hancockii* is a distinct species, in agreement with our morphological and secondary metabolite findings. All three trees indicate that the most closely related species is *A*. *leporis* [16], which shows similarity with *A*. *hancockii* in having a floccose, lightly sporing habit and bullet shaped sclerotia, a distinctive feature seen in closely related *Aspergillus* species. *A*. *leporis* differs from *A*. *hancockii* by producing shorter conidiophores, larger, olive brown conidia and columnar rather than radiate conidial chains. Secondary metabolite production is also distinct, with no compounds in common with *A*. *hancockii* [17].

### Genome assembly and gene prediction

Velvet was used to assemble the 1000bp paired end reads from *A*. *hancockii*. In total, 99.6% of all available reads were assembled into 1874 scaffolds, comprisinf 3102 contguous sequences. The GC content of the genome was approximately 43%. The total assembled genome size was 40,074,632bp, of which the longest contiguous sequence was 165,236bp. The N50for the assembly was 83,637bp. AUGUSTUS gene prediction indicated that the genome consisted of at least 11,240 genes.

### Enzyme profiling

Results from a preliminary screening of enzyme production by *A*. *hancockii* on AZCL assay plates with an azur-linked substrate are given in Table 2. The strongest secreted enzyme activity was observed for enzymes breaking down hemicellulosic compounds (arabinoxylanase and endoxylanase). Moderate activities were observed against other hemicellulose components (e.g. endo-1,4-β-mannanase) and enzyme activities relevant for decomposition of cellulose and starch (endoglucanases and α-amylase, respectively).

The PPR/HotPep analysis of the genome of *A*. *hancockii* showed that this species possesses genes encoding a wide range of types of carbohydrate active enzyme. A selection of the most interesting results is given in Table 3. The complete list of enzymes found that are capable of modifying carbohydrate is given (Table B in S1 File).

**Table 3:** Summarised PPR/Hotpep analysis of *Aspergillus hancockii* FRR 3425 genome with respect to plant polysaccharide degrading enzymes and selected intracellular functions. The enzyme functions are sorted according to the substrate on which they normally act. In many cases several genes in several different families represent the same enzymatic function. (See full list of hits in Table B in S1 File).

Substrate	EC number	Enzyme function	No. of genes	Enzyme families representing function			
Cellulose	3.2.1.176	1,4-β-Cellobiosidase (reducing end)	3	3 GH7	0	0	0
	3.2.1.91	1,4-β-Cellobiosidase (nonreducing end)	1	1 GH6	0	0	0
	3.2.1.4	Endo-β-1,4-glucanase	5	1 GH12	4 GH5	0	0
	3.2.1.21	β-Glucosidase	21	4 GH1	17 GH3	0	0
Hemicellulose	3.2.1.8	Endo-1,4—Xylanase	17	10 GH10	6 GH11	1GH43	0
	3.2.1.37	1,4-β-Xylosidase	7	2 GH3	3 GH43	2 GH5	0
	3.1.1.72	Acetylxylanesterase	4	1 CE1	1 CE2	2 CE4	0
	3.2.1.55	α-*N*-Arabinofuranosidase	12	2 GH43	4 GH51	1 GH54	5
	3.2.1.78	Endo-1,4-β-mannosidase	3	1 GH26	2 GH5	0	GH62
	-	LPMO	19	14 AA9	5 AA11	0	0
Lignin	1.10.3.2	Laccase	8	8 AA1	0	0	0
Pectin	4.2.2.2	Pectate lyase	4	1 PL1	3 PL3	0	0
	4.2.2.10	Pectin lyase	8	8 PL1	0	0	0
	3.2.1.15	Polygalacturonase	9	9 GH28	0	0	0
Starch	3.2.1.1	α-Amylase	4	4 GH13	0	0	0
	3.2.1.3	1,4-α-Glucosidase	3	3 GH15	0	0	0
	3.2.1.20	α-Glucosidase	7	4 GH13	3 GH31	0	0
Chitin/chitosan	3.2.1.14	Chitinase	17	17 GH18	0	0	0
	3.2.1.132	Chitosanase	5	5 GH75	0	0	0
Cutin	3.1.1.74	Cutinase	4	4 CE5	0	0	0
Fructan	3.2.1.80	β-Fructosidase	4	4 GH32	0	0	0
Cell wall modification glycosylation etc	2.4.1.183	α-1,3-Glucan synthase	3	3 GH13	0	0	0
	3.2.1.6	Endo-1,3(4)-β-glucanase	2	2 GH16	0	0	0
	2.4.1.16	Chitin synthase	8	8 GT2	0	0	0
	3.2.1.39	Endo-1,3-β-d-glucosidase	4	2 GH16	1 GH17	1 GH81	0
	3.2.1.58	1,3-β-Glucosidase	3	2GH5	1 GH55	0	0
	3.2.1.59	Endo-1,3-α-glucosidase	21	11 GH71	0	0	0

It appears that *A*. *hancockii* is potentially a broad and versatile biomass degrader, with genes encoding for breaking down a multitude of substrates including cellulose, hemicellulose, lignin, pectin, starch, chitin, cutin and fructan (Table 2 and Table B in S1 File). Furthermore and interestingly, some of the functions are represented on the genome from more than one type of gene, belonging to different enzyme families. In particular, hemicellulose-modifying enzymes are each represented by several types of enzyme genes (see e.g. 3.2.1.55, α-*N*-arabinofuranosidase, represented on the genome by four families, GH43, GH51, GH54, GH62).

A third layer of diversity in the *A*. *hancockii* genome is that most genes are represented in several genes or variants. The most well-represented enzymatic function found was 3.2.1.21 β-glucosidase, which breaks down sugar dimers to monomers. This function was found to be represented by as many as 21 genes in the *A*. *hancockii* genome. A high number of genes were also found for 3.2.1.8 endo-1,4-β-xylanase and 3.2.1.14 chitinase (both 17 genes or gene copies).

Interestingly, genes encoding two different types of lytic polysaccharide monoxygenase (LPMO) enzymes were found by mining the *A*. *hancockii* genome by PPR/HotPep analysis: AA9 (14 genes in 7 subgroups) and AA11 (5 genes in 3 subgroups) in the genome (see Table 2). The peptide patterns used for the LPMO analysis were expanded beyond the Cazy database and validated in a recent study [18]. LPMO enzymes are primarily described to be active on crystalline cellulose, acting in synergy with endoglucanases (EC 3.2.1.4). Recently, LPMO enzymes have been shown to act also on hemicellulose [19], and on starch [20].

As described above, the *A*. *hancockii* genome has genes encoding a broad and versatile enzyme profile relevant to plant biomass decomposition. Only the cellulase profile is relatively weak, as strong cellulose-degrading fungi typically possess more than one type of enzyme with endoglucanase activity (EC 3.2.1.4), typically GH5, GH 12, GH 9 and GH45, plus more types of protein families with a 3.2.1.4 type of function. The rather weak activity on cellulose is also confirmed by the AZCL assay on HE-cellulose (Table 2). Interestingly, a very strong LPMO profile was found: AA9 (14 genes) and AA11 (5 genes) in the genome. LPMO enzymes were first associated with acting synergistically with GH cellulases in degrading cellulose. Recently it has been documented that LPMO enzymes can also play a role in breaking down hemicellulosic structures, and a subdivision of the LPMO enzymes based on peptide pattern recognition has been suggested recently [18]. Further studies are needed to characterise the distribution at subfamily level of the LPMO enzymes found in *Aspergillus* species and to characterise and document the diversity and variation among the many LPMO genes found in the genome of this type of fungus.

### Secondary metabolites

Analytical scale growth of *Aspergillus hancockii* on a range of liquid, agar and grain-based media resulted in moderate to high levels of secondary metabolites, with productivity and metabolite diversity superior on grains, particularly rice-based media (Figures A and B in S1 File). Preparative scale cultivation of *A*. *hancockii* on rice for 21 days gave confluent coverage with a luxurious mass of aerial mycelium. The entire rice cultivation was extracted with acetone to give an aqueous slurry after evaporation. The slurry was partitioned against ethyl acetate, evaporated to a gummy residue, and then defatted with hexane against 10% H_2_O/MeOH to produce an enriched extract. The bulk of the secondary metabolites were located in the methanolic extract, with no selective loss of any specific metabolite on enrichment. Fractionation and chemical analysis of the crude extract by C_18_ preparative HPLC and Sephadex LH-20 chromatography yielded 15 major metabolites, 11 of which had not been previously reported at the time of publication. The metabolic capability of *A*. *hancockii* adds further recognition of the interplay of novel chemical diversity and taxonomic uniqueness within subgenus *Circumdati*, as observed with our recent discovery of kumbicins as metabolites of *A*. *kumbius* [21].

**Fig 2:**
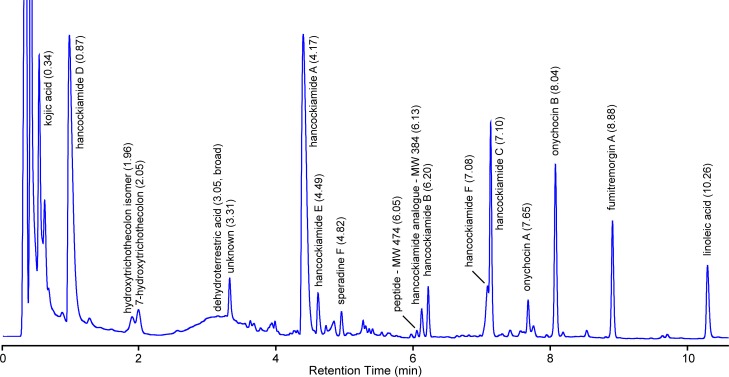
HPLC profile (210 nm) of *Aspergillus hancockii* after cultivation on rice for 21 days.

The methanol extract from *A*. *hancockii* grown on rice for 21 days was separated by gradient HPLC (Fig 2) and analysed using DAD and positive/negative ESIMS TIC traces (Figures C and D in S1 File). Assessment of the co-metabolite diversity was undertaken using UV detection at 210 nm, with 69 peaks being responsible for 99.5% of the total area under metabolite peaks (AUC) from 0.5 to 10.5 min. Analysis of the abundance of the secondary metabolites revealed a hyper-dispersed distribution, with 16 metabolites accounting for 90% of total metabolite AUC, and the remaining 52 metabolites present at only trace levels. At these low levels, retention time, UV-vis and ESIMS allowed only partial characterisation of minor co-metabolites (Table K in S1 File). Linoleic acid (*t*_R_ 10.26 min) is an endogenous fatty acid extracted from grains and many cultivation media and served as an implicit nonpolar standard for comparisons.

The extracts of *A*. *hancockii* showed no metabolite overlap with any other species in the *A*. *alliaceus* clade. Indeed, *A*. *hancockii* shares no secondary metabolites in common with its closest ITS neighbour *A*. *leporis*. A search of UV-vis spectra of the major HPLC non-polar metabolites on COMET [14] identified fumitremorgin A as the only recognizable fungal metabolite present in the extract. Fumitremorgin A [22] (*t*_R_ 8.88 min, 3.46%, λ_max_ 196, 226, 276, 294 nm; ESIMS *m/z* [M–H]^–^ 578, [M+H]^+^ 580) was the most non-polar of the major secondary metabolites. The fumitremorgins are broadly distributed in *Penicillium* and *Aspergillus* species and have limited diagnostic value as species specific metabolite markers. Minor analogues bearing the distinctive fumitremorgin UV-vis spectrum eluted at 5.34, 5.95, 7.24, 7.72, 7.90, 8.50 and 8.78 min.

**Fig 3:**
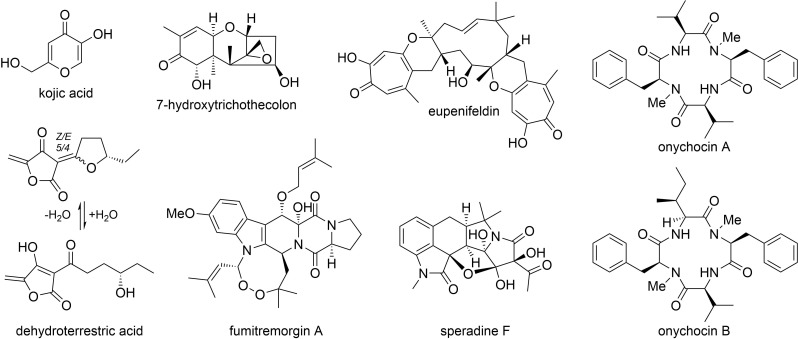
Metabolites isolated from *Aspergillus hancockii*.

The most characteristic feature of the *A*. *hancockii* HPLC trace was the broad peak eluting between 2–4 min when chromatographed using 0.01% TFA phase modifier. This elution behaviour is rare and has not previously been encountered by us within *Aspergillus*. The use of strongly acidic conditions (0.1% TFA) improved the peak shape and allowed purification of the compound, which was identified as the novel metabolite, dehydroterrestric acid (*t*_R_ 3.05 min, 14.9%; λ_max_ 246, 292 nm; ESIMS *m/z* [M+H_2_O–H]^–^ 225, [M+H]^+^ 209). Analysis of the ^1^H and ^13^C NMR spectra of dehydroterrestric acid (Table J in S1 File; Figures T and U in S1 File) revealed an equilibrating 5/4 mixture of *Z*- and *E*-isomers, which has been previously observed for the closely related *Penicillium* metabolites terrestric acid, carolic acid and carlic acid [23]. In DMSO-*d*_6_ solution, dehydroterrestric acid exists exclusively as a cyclic ether, while in aqueous solution, the cyclic form is in equilibrium with a ring-opened hydrated form (Fig 3). This equilibrium is further complicated by the interconversion of tetronic acid tautomers in aqueous solution, as well as their potential to complex with available metal cations. Indeed, analysis of the ESIMS spectra of dehydroterrestric acid in aqueous solution revealed putative complexes of the hydrated form with Na^+^ (*m/z* [2M–2H+Na]^–^ 473; [3M–2H+Na]^–^ 699), Ca^2+^ (*m/z* [3M–3H+Ca]^–^ 715) and Fe^2+^ [3M–3H+Fe]^–^ 731). The poor chromatographic properties of dehydroterrestric acid complicated analysis of the minor metabolites eluting from 2 to 4 min, with most peaks in this region containing traces of the acid. It is noteworthy that the same broad peak shape dominates the HPLC trace of the terrestric acid producer, *Penicillium simplicissimum*.

A more conventional elution behaviour was displayed by a novel family of substituted piperazines, which we have named the hancockiamides. The hancockiamides are related to the brasiliamides reported from *Penicillium brasilianum* [24, 25] and full details of their structure elucidation and characterisation will be reported elsewhere. Hancockiamide D (*t*_R_ 0.87 min, 18.4%; λ_max_ 206, 230s, 246s, 270tr nm; ESIMS *m/z* [M+H]^+^ 343), hancockiamide A (*t*_R_ 4.17 min, 20.3%; λ_max_ 206, 280 nm; ESIMS *m/z* [M–H]^–^ 471, [M+H]^+^ 473) and hancockiamide C (*t*_R_ 7.10 min, 6.52%; λ_max_ 208, 282 nm; ESIMS *m/z* [M+H]^+^ 513) were the dominant metabolites on grains, while hancockiamide F (*t*_R_ 7.08 min, 2.66%; λ_max_ 206, 274 nm; ESIMS *m/z* [M–H]^–^ 527, [M+H]^+^ 529) was the dominant metabolite in many liquid media. Hancockiamide E (*t*_R_ 4.49 min, 1.56%; λ_max_ 208, 282 nm; ESIMS *m/z* [M–H]^–^ 545, [M+H]^+^ 547), an uncharacterised hancockiamide analogue (*t*_R_ 6.13 min, 2.14%; λ_max_ 212, 254, 282 nm; ESIMS *m/z* [M–H]^–^ 383, [M–H_2_O+H]^+^ 367) and hancockiamide B (*t*_R_ 6.20 min, 1.52%; λ_max_ 206, 280 nm; ESIMS *m/z* [M–H]^–^ 383, [M–H_2_O+H]^+^ 367) were present in smaller amounts, while additional minor analogues eluting at 4.69 and 8.15 min were detectable but insufficient material was recovered to resolve to unique structures.

A pair of non-polar cyclic tetrapeptides, onychocin A (*t*_R_ 7.65 min, 0.89%, λ_max_ 210 nm; ESIMS *m/z* [M–H]^–^ 519, [M+H]^+^ 521) and onychocin B (*t*_R_ 8.04 min, 5.42%, λ_max_ 210 nm; ESIMS *m/z* [M–H]^–^ 533, [M+H]^+^ 535), were identified and named in recognition of their prior discovery by Bills and colleagues from the Arachnomycetale, *Onychocola sclerotica* [26]. The metabolites are underrepresented in the 210 nm DAD trace, but are dominant peaks in the positive and negative ESIMS TIC traces (Figure D in S1 File). The structures of onychocin A (*cyclo*-[l-*N*-Me-Phe, l-Val]_2_) and onychocin B (*cyclo*-[l-*N*-Me-Phe, l-Val, l-*N*-Me-Phe, l-Ile]) were confirmed by acid hydrolysis and Marfey’s analysis [27](Figures V and W in S1 File). Onychocin B eluted as an inseparable mixture containing 15% of onychocin D (*cyclo*-[l-*N*-Me-Phe, l-Val, l-*N*-Me-Phe, l-Leu]). The onychocins have thus far not been reported as metabolites of *Pencillium* or *Aspergillus* species. An additional, structurally unrelated cyclic peptide (*t*_R_ 6.02 min, 0.13%; λ_max_ 192, 222, 278 nm; ESIMS *m/z* [M–H]^–^ 473, [M+H]^+^ 475) was observed as eluting prior to hancockiamide B. This peptide exhibits poor solubility in common solvents and its abundance is likely underestimated based on the DAD 210 nm spectrum and the positive and negative ESIMS TIC traces.

The polar metabolites were identified as kojic acid [28] (*t*_R_ 0.32 min, 25.2%; λ_max_ 192, 216, 268 nm; ESIMS *m/z* [M+H]^+^ 143) and an unknown high molecular weight, <190 nm metabolite eluting at 0.50 min (*t*_R_ 0.50 min, 6.17%; λ_max_ <190 nm; ESIMS *m/z* [M–H]^–^ 746, [M+H]^+^ 748). A novel trichothecene metabolite, 7-hydroxytrichothecolon (*t*_R_ 2.05 min, 1.04%; λ_max_ 224 nm; ESIMS *m/z* [M+H]^+^ 281) and its uncharacterised isomer (*t*_R_ 1.95 min, 1.10%; λ_max_ 224 nm; ESIMS *m/z* [M+H]^+^ 281) are the first reported metabolites related to trichothecenes from *Aspergillus* species.

In the intermediate polarity region from 3–5 min, many of the minor co-metabolites were masked by dehydroterrestric acid. Other than hancockiamides A and E, only a single class of three analogues was observed, having a distinctive UV shape (λ_max_ 220, 264, 314 nm) with diagnostic value. The most abundant analogue of this class was purified and identified as the oxindole, speradine F [29] (*t*_R_ 4.82 min, 0.80%; λ_max_ 218, 264, 314 nm; ESIMS *m/z* [M–H]^–^ 413, [M+H]^+^ 415). HPLC peaks eluting at 3.96 and 4.92 min with similar UV-vis maxima were tagged as putative minor analogues of speradine F, but insufficient material was available for isolation and characterisation. Speradines are a family of multicyclic oxindoles that have been previously reported from *A*. *tamarii* [30], *A*. *oryzae* [29, 31] and *A*. *flavus* [32]. Confusingly, speradine F was originally reported from *A*. *oryzae* [29], then later as speradine B from *A*. *flavus* [32], and again as penicamedine A from *Penicillium camemberti* [33]. An *N*-desmethyl analogue of speradine F was recently reported from *P*. *dipodomyicola* [34] and was also erroneously named speradine B.

During the analysis of *A*. *hancockii* cultivation extracts, a distinctive non-polar metabolite was frequently but erratically observed. Purification using normal phase silica led to the identification of eupenifeldin [35] (*t*_R_ 7.65 min, N/A%; λ_max_ 250, 324s, 363 nm; ESIMS *m/z* [M–H]^–^ 547, [M+H]^+^ 549) as an important co-metabolite of *A*. *hancockii*. Over time, or on cold storage, eupenifeldin precipitates from methanolic solutions and its presence cannot be reliably observed or quantified. While extraction with water-miscible solvents such as methanol offers a good compromise between polar and non-polar chemistries, the apparent disappearance of distinct compounds is nonetheless common. While such “losses” can be the result of degradation or volatility, most often they are due to precipitation as either an oil or solid on standing.

### Secondary metabolite biosynthetic gene clusters

Like many of the previously sequenced *Aspergillus* species [36], the genome of *A*. *hancockii* is rich in secondary metabolite biosynthetic genes. The genome encodes 26 polyketide synthase (PKS) genes (Table 4), 16 multimodular nonribosomal peptide synthases (NRPSs), and 15 single modular NRPS-like enzymes (Table 5). In particular, *A*. *hancockii* encodes four PKS-NRPS hybrids. Several genes involved in terpene biosynthesis were also identified in the *A*. *hancockii* genome (Table 6). Using AntiSMASH 3.0 [9], a total of 75 secondary metabolite biosynthetic gene clusters (BGCs) were readily identified in the *A*. *hancockii* genome. Only 15 out of the 75 predicted BGCs in *A*. *hancockii* were identified by AntiSMASH to be homologous to known BGCs deposited on the MIBiG database [10], suggesting that *A*. *hancockii* is enriched with unique and diverse secondary metabolite biosynthetic capabilities. When the ClusterFinder algorithm [37] is enabled in the AntiSMASH 3.0 search parameters, over 100 putative biosynthetic gene clusters can be identified in the *A*. *hancockii* genome. Some of the compounds we have identified in the culture extracts can be easily mapped to the corresponding biosynthetic gene clusters in the *A*. *hancockii* genome based on bioinformatics analysis and prior biosynthetic studies on these identical or related compounds, including the use of various strategies described previously [38].

**Table 4:** Polyketide synthases (PKS) encoded in the *A*. *hancockii* genome.

No.	Contig#	Type	Domain Architecture	Closest BLAST Homologue*	Identity / Coverage (%)
1	23	HR-PKS	KS-AT-DH-cMT-ER-KR-ACP	*Aureobasidium subglaciale* AUEXF2481_7276	46/97
2	105	HR-PKS	KS-AT-DH-cMT-ER-KR-ACP	*Aspergillus clavatus* ACLA_087700	73/100
3	891.3	HR-PKS	KS-AT-DH-cMT-ER-KR-ACP	*A*. *kawachii* AKAW_05432	58/97
4	990.4	HR-PKS	KS-AT-DH-cMT-ER-KR-ACP	*A*. *clavatus* ACLA_098370	76/99
5	1005.6	HR-PKS	KS-AT-DH-cMT-ER-KR-ACP	*A*. *nomius* ANOM_009863	57/98
6	874.4	HR-PKS	KS-AT-DH-cMT-KR-ACP	*Penicillium roquefortii* PROQFM164_S01g002430	57/93
7	990.2	HR-PKS	KS-AT-DH-cMT-KR-ACP	*P*. *camembertii* PCAMFM013_S001g000382	68/98
8	3558	HR-PKS	KS-AT-DH-ER-KR-ACP	*Talaromyces stipitatus* TSTA_030950	55/100
9	1163.2	PKS-NRPS	KS-AT-DH-cMT-KR-ACP-C-A-T-R	*A*. *oryzae* CpaA	76/99
10	1750.3	PKS-NRPS	KS-AT-DH-cMT-KR-ACP-C-A-T-R	*A*. *clavatus* ACLA_077660	61/99
11	2060.2	PKS-NRPS	KS-AT-DH-cMT-KR-ACP-C-A-T-R	*Oidiodendron maius* OIDMADRAFT_169065	53/100
12	2865.3	PKS-NRPS	KS-AT-DH-cMT-KR-ACP-C-A-T-R	*Pseudogymnoascus pannorum* V493_00906	88/100
13	1572.1	PR-PKS	KS-AT-DH-KR-ACP	*A*. *flavus* AFLA70_255g001141	76/100
14	1532.1	PR-PKS	KS-AT-DH-KR-ACP	*Trichophyton tonsurans* TESG_01634	56/99
15	103	NR-PKS	SAT-KS-AT-PT-ACP	*A*. *rambellii* ARAM_002866	65/99
16	189	NR-PKS	SAT-KS-AT-PT-ACP	*A*. *ochraceoroseus* AOCH_005067	70/96
17	1051.1	NR-PKS	SAT-KS-AT-PT-ACP	*A*. *terreus* ATEG_08451	71/100
18	2835.4	NR-PKS	SAT-KS-AT-PT-ACP	*Neosartorya fischeri* NFIA_112240	82/87
19	703.3	NR-PKS	SAT-KS-AT-PT-ACP-ACP-TE	*A*. *flavus* PksP	87/99
20	478.2	NR-PKS	SAT-KS-AT-PT-ACP-cMT	*A*. *flavus* AFLA_127090	75/99
21	331.2	NR-PKS	SAT-KS-AT-PT-ACP-cMT-R	*Acremonium strictum* MOS	57/99
22	1544.7	NR-PKS	SAT-KS-AT-PT-ACP-cMT-R	*A*. *parasiticus* P875_00117035	72/100
23	1448	NR-PKS	SAT-KS-AT-PT-ACP-TE	*A*. *parasiticus* P875_00034103	82/98
24	816.2	Type III PKS	KS	*P*. *camembertii* PCAMFM013_S001g000702	64/96
25	1157.1	Type III PKS	KS	*P*. *camemberti* PCAMFM013_S001g000702	70/87
26	1174.2	Type III PKS	KS	*A*. *flavus* AFLA70_138g002461	66/92

**Table 5:** Nonribosomal peptide synthases (NRPSs) and NRPS-like enzymes encoded in the *A*. *hancockii* genome (excluding PKS-NRPS hybrids).

No.	Contig#	Domain Architecture	Closest BLAST Homologue	Identity / Coverage (%)
1	989	C-A-T-C-T	*Aspergillus flavus* AFLA70_124g001810	73/99
2	874.2	C-A-T-C-A-T-C-CA-T-C-A-C	*A*. *kawachii* AKAW_00537	42/99
3	499.2	C-A-T-C-A	*A*. *fumigatus* GliP	52/95
4	990.4	C-A-T-C	*Tolypocladium ophioglossoides* TOPH_03419	50/98
5	2373.5	A-T-C-T-C-A-T-C	*Talaromyces stipitatus* TSTA_122180	55/99
6	331.2	A-T-C-T-C	*A*. *oryzae* AOR_1_3209	62/99
7	1572.6	A-T-C-T	*A*. *nomius* ANOM_011584	83/99
8	729.1	A-T-C-A-T-E-C-T-C-T	*A*. *flavus* Pes1-like AFLA70_100g002281	81/99
9	400.4	A-T-C-A-T-C-T-C-T-C	*A*. *oryzae* AOR_1_910144	80/100
10	1031	A-T-C-A-T-C-A-T-E-C-T-CA-T-C	*A*. *flavus* AFLA70_124g001810	80/90
11	20	A-T-C-A-T-C-A-T-C-A-T-C-A-T-C	*Trichoderma virens* TRIVIDRAFT_52861	55/99
12	6	A-T-C-A-T-C	*A*. *nomius* ANOM_008097	74/92
13	43	A-T-C-A-T-C	*A*. *clavatus* ACLA_061190	79/99
14	1051.1	A-T-C-A-T-C	*Neosartorya udagawae* NPS7/AUD_3775	70/98
15	1189.3	A-T-C-A-T-C	*A*. *fumigatus* ftmA	86/100
16	284.1	A-T-C-A	*Penicilliom nordicum* ACN38_g12483	41/73
17	498.1	A-T-TE	*P*. *brasilianum* PMG11_03269	67/99
18	91	A-T-TE	*A*. *nomius* ANOM_000859	63/98
19	319.2	A-T-R-KR	*A*. *nomius* ANOM_004242	92/100
20	2835.4	A-T-C	*Uncinocarpus reesii* UREG_04218	46/98
21	2835.4	A-T-R	*P*. *brasilianum* PMG11_03954	58/96
22	990.3	A-T-R	*A*. *terreus* ATEG_09068	51/98
23	21	A-T-R	*A*. *oryzae* AOR_1_1954154	93/98
24	484	A-T-R	*N*. *udagawae* AUD_5118	87/97
25	61	A-T-R	*A*. *ustus* HK57_00285	77/89
26	371	A-T-R	*A*. *flavus* AFLA70_2g008790	72/97
27	466	A-T-R	*A*. *flavus* AFLA70_1g009780	78/100
28	1033.1	A-T-R	*Rasamsonia emersonii* T310_6522	40/92
29	1429	A-T-R	*T*. *cellulolyticus* TCE0_043r15669	62/98
30	2472.1	A-T-R	*A*. *flavus*	74/99
31	723.2	A-T-R	*A*. *oryzae* AO090003000945	75/75

**Table 6:** Genes involving in terpene biosynthesis in the *A*. *hancockii* genome.

No.	Contig#	Putative function	Closest BLAST Homologue	Identity / Coverage (%)
1	305	Trans-Isoprenyl Diphosphate Synthase	*Aspergillus flavus* AFLA70_138g002381	72/99
2	204.1	Phytoene synthase	*A*. *flavus* AFLA_102510	77/100
3	726	Squalene cyclase	*A*. *nomius* ANOM_002681	60/92
4	54	Squalene cyclase	*A*. *flavus* AFLA_102160	86/98
5	67	Squalene cyclase	*Neosartorya udagawae* AUD_3717	73/98
6	528	Squalene synthase	*A*. *nomius* ANOM_007771	86/100
7	755.1	Sesquiterpene synthase	*Stachybotrys chartarum* Tri5	70/92

The gene cluster encoding the biosynthesis of fumitremorgin A was readily identified from the *A*. *hancockii* genome by comparison with fumitremorgin gene clusters in *A*. *fumigatus* and *Neosartorya fischeri* [39]. Using AntiSMASH 3.0, we were also able to identify the homologous gene cluster (matching with BGC0000356_c1). The *A*. *hancockii* NRPS encoded by g8247.t1 shares 96% protein identity with *N*. *fischeri* NRPS FtmPS, which is responsible for biosynthesis of brevianamide F, the precursor of the fumitremorgins and verruculogen. Fumitremorgin A is known to be produced by *N*. *fischeri*, while *A*. *fumigatus* only produces fumitremorgin B and verruculogen. Fumitremorgin A has the same core structure as verruculogen, except that it has an additional *O*-prenyl group not found in fumitremorgin B and verruculogen. A previous study identified a verruculogen *O*-prenyltransferase (NFIA_093390) in *N*. *fischeri*, although the gene was not encoded in the *N*. *fischeri* main fumitremorgin gene cluster, but rather on a separate locus in the genome [40]. Interestingly, we found a homologue of NFIA_093390 in *A*. *hancockii* (g8245.t1, 98.9% protein identity) clustered together with other genes required for the biosynthesis of verruculogen on contig 1189.3. The clustering of homologous biosynthetic pathways in one species but scattering into multiple smaller gene clusters in another has been observed previously, e.g. tryptoquialanine gene cluster [41] and dothistromin gene cluster [42]. This highlights the utility of comparative genomics for identification of the complete gene sets required for biosynthesis of specific secondary metabolites [38].

Kojic acid biosynthetic genes have been identified previously from *A*. *oryzae* [43] and shown to involve a FAD-dependent oxidoreductase (KojA) and a major facilitator superfamily transporter (KojB) in the biosynthesis. Both KojA and KojB homologues were identified in the *A*. *hancockii* genome, g2128.t1 and g2130.t1, sharing 95% and 92% protein identities respectively. The genes were within close proximity of each other and are located on contig 208.

AntiSMASH analysis identified an *A*. *hancockii* PKS-NRPS gene cluster on contig 687 that is homologous to the cyclopiazonic acid biosynthetic gene cluster (BGC0000977_c1). Speradine F is a highly oxygenated and *N*-methylated analogue of cyclopiazonic acid and thus is likely to be encoded by the above PKS-NRPS gene cluster in *A*. *hancockii*. The *A*. *hancockii* PKS-NRPS encoded by g8092.t1 on contig 687 shares 73% head to tail protein identity with CpaA from *A*. *flavus* and *A*. *oryzae* [44]. All of the other genes found in the *A*. *oryzae cpa* gene cluster are also present in *A*. *hancockii* [45], including *cpaM* (g6126.t1 in *A*. *hancockii*, 60% protein identity), which has been demonstrated to encode an *N-*methyltransferase responsible for converting 2-oxo-cyclopiazonic acid to speradine A [46]. Additional oxidations and ring closure will allow the formation of the sixth ring (E ring) of speradine F via speradine A. The genes/enzymes responsible for the additional oxidations remain to be determined, but the P450 oxygenase CpaH in *A*. *hancockii* may carry out such oxidations besides the 2-indole position on 2-oxo-cyclopiazonic acid [45].

Trichothecenes are an important class of sesquiterpenoid mycotoxins that are best known from *Fusarium* species, and have also been identified from other filamentous fungi [47, 48], but so far have been reported only from the order Hypocreales (class Sordariomycetes) [49]. A gene cluster on *A*. *hancockii* contig 755.1 was identified as homologous to trichothecene biosynthetic gene cluster (BGC0000931_c1) by AntiSMASH. The finding corresponds to our identification of 7-hydroxytrichothecolon, structurally related to deoxynivalenol, a type B trichothecene. The oxygenation pattern (at position C-4 but not C-3) of the cyclopentane ring on 7-hydroxytrichothecolon is distinct from 3,4-oxygenated trichothecenes that are characteristic of *Fusarium* species, but similar to those isolated from other fungi, including *Trichoderma* species. This suggests that the biosynthetic pathway in *A*. *hancockii* branches earlier, going through the isotrichodiol intermediate, which is converted to 12,13-epoxytrichothec-9-ene [47].

Relatively few biosynthetic gene clusters for trichothecenes have been described from fungi other than *Fusarium* species, notable examples being from *Trichoderma* and *Stachybotrys* species [49, 50]. The *A*. *hancockii* trichodiene synthase homologue (encoded by g6521.t4) shares 68% protein identity with *Fusarium poae* TRI5, 65% with *Stachybotrys chartarum* TRI5 and 53% with *Trichoderma brevicompactum* TRI5. The TRI4 homologue in *A*. *hancockii*, which is a cytochrome P450 enzyme predicted to catalyse multiple oxidations of trichodiene to yield isotrichodiol (but not isotrichotriol), shares 73% protein identity to *Fusarium* TRI4 [51]. As expected, TRI101, which has been shown to acetylate the 3-OH of the isotrichodermol intermediate in *Fusarium* [52], is missing in the corresponding gene cluster in *A*. *hancockii* as the isolated compound 7-hydroxytrichothecolon lacks a hydroxyl group at the C-3 position. The TRI7 homologue responsible for acetylating the 4-OH in *Fusarium* species is also missing in the *A*. *hancockii* gene cluster. A homologue of *Fusarium graminearum* TRI1 [53] can also be found in *A*. *hancockii* (g6523.t1), corresponding to the hydroxylation of the C-7 position on 7-hydroxytrichothecolon. To our knowledge, *A*. *hancockii*, which belongs to the class Eurotiomycetes, is the first fungus outside the class Sordariomycetes reported to possess a trichothecene biosynthetic gene cluster and produce trichothecene analogues. This expands the previously known taxonomic distribution of trichothecenes in fungi.

Dehydroterrestric acid is a polyketide with an interesting tetronic acid moiety. An analogue of this compound, terrestric acid, was previously isolated from *Penicillium griseoroseum* [54]. Terrestric acid is also related to other tetronic acids with shorter carbon chain, such as carlosic and carolic acids, and agglomerins [55–57]. Previous isotope-feeding studies suggested that a C_4_ dicarboxylic acid precursor is involved in their biosynthesis [58]. This is supported by the recent identification of a PKS-NRPS (CaaA) from *A*. *niger* that produces carlosic acid and agglomerin F [59]. The study suggests that the unusual NRPS module of the enzyme is capable of activating malic acid to form an ester linkage between the activated malic acid and the polyketide acyl chain synthesised by the PKS module. This is followed by a Dieckman cyclisation and release of the product as a tetronic acid [59]. A homologue of CaaA was identified in *A*. *hancockii* (encoded by g9274.t1, sharing 64% identity and 77.5% similarity) along with the trans-enoyl reductase CaaB homologue (encoded by g9278.t1, sharing 70.5% protein identity), which work together with the PKS-NRPS. The *A*. *hancockii* putative dehydroterrestric acid biosynthetic gene cluster also contains a homologue of the cytochrome P450 oxygenase CaaC proposed to be involved in the decarboxylation and formation of the exocyclic methylene common to both carlosic acid and dehydroterrestric acid.

Besides fumitremorgin A, several NRPS-derived compounds were isolated from *A*. *hancockii*. The two *N-*methylated cyclic tetrapeptides, onychocins A and B, are likely to be produced by a tetramodular NRPS based on the collinearity rule commonly observed in both bacterial and fungal NRPSs [60]. However, given that onychocins A and B consist of two pairs of similar amino acids (l-Phe and l-Val or l-Ile), it is possible that the tetrapeptides are biosynthesised by non-canonical NRPSs with functional domains that are capable of acting iteratively, such as those commonly observed in cyclooligomer depsipeptide biosynthesis [61] and more recently demonstrated in the biosynthesis of fungisporin from *Penicillium chrysogenum* [62]. Interestingly, of all the multimodular NRPSs encoded in the genome of *A*. *hancockii*, we did not identify any NRPS that harbours the *N*-methyltransferase domain, such as that identified in enniatin synthetase [63] and cyclosporine synthetase [64]. Therefore, it is possible that the *N*-methylation of the onychocins may be catalysed by a standalone *N-*methyltransferase. Further molecular genetic studies are required to identify the NRPSs responsible for the biosynthesis of the onychocins and other nonribosomal peptides (i.e. hancockiamides and the cyclic tetrapeptide unrelated to onychocins) isolated from *A*. *hancockii*.

### Taxonomic description–*Aspergillus hancockii* Pitt sp. nov Fig 4

CYA, 25°C, 7 days: Colonies 60 mm or more in diameter but remaining distinct from a three point inoculation, plane; margins entire; mycelium deep and floccose, but quite sparse, white or off-white; sporulation usually sparse, with scattered radiate conidial heads near colony centres or margins, coloured Greyish Green to Olive under the stereomicroscope (30D–E5); sclerotia characteristically produced at colony centres, often enveloped by aerial mycelium, and later developing at margins, on the agar or in aerial mycelium, white at first, becoming black, spherical, ellipsoidal or irregular, developing slowly, when mature black and rock hard, 500–1200 × 500–800 μm; clear exudate sometimes produced around colony centres; soluble pigment absent; reverse uncoloured or Buff to Light Orange (5A–B4–5).

**Fig 4:**
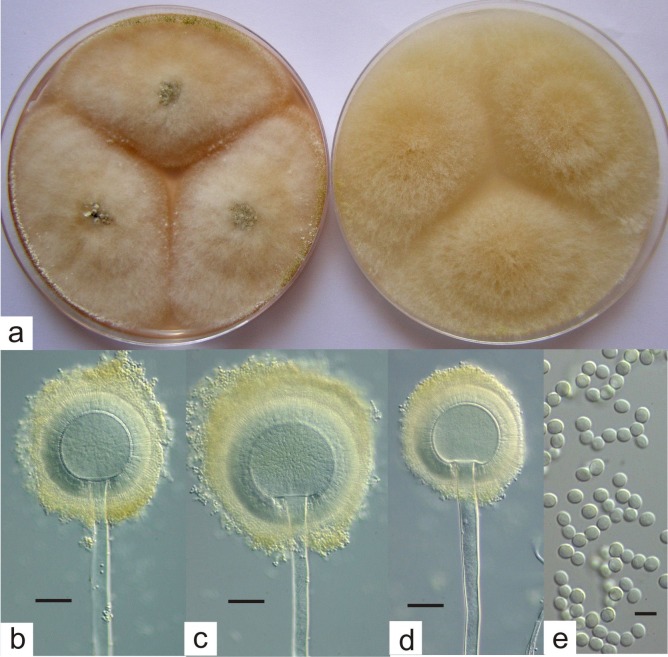
*Aspergillus hancockii* (a) colonies on CYA (left) and MEA (right), 7 days, 25 °C, (b)-(d) fruiting structures, bars 20 μm, (e) conidia, bar 5 μm.

MEA, 25°C, 7 days: Colonies 60 mm or more in diameter, plane, usually covering the whole Petri dish; mycelium deep and floccose, white to slightly grey; sporulation usually occurring at colony centres, but often obscured by the mycelium, coloured as on CYA; sclerotia sometimes evident, as on CYA; exudate and soluble pigment absent; reverse uncoloured or dull yellow brown.

G25N, 25°C, 7 days: Colonies 20–30 mm in diameter, deep and floccose, plane; mycelium white to pale yellow; sporulation scattered, coloured as on CYA; exudate and soluble pigment absent; reverse uncoloured.

37°C, CYA, 7 days: Colonies 12–20 mm in diameter, centrally raised to crateriform; margins fimbriate, white or brown; mycelium velutinous or floccose; sporulation absent; reverse dull to dark brown.

Conidiophores borne from surface or aerial hyphae, sometimes from high in the aerial mycelium, stipes very long (2-)3-6 mm × 7-10(-15) μm, with thick, smooth walls; vesicles spherical, (30-)40-60(-70) μm in diameter, fertile over the entire surface; metulae and phialides packed very tightly, metulae cylindroidal, 9–10 × 4–5 μm, phialides acerose, 8–10 × 2.0–2.5 μm; conidia small, spherical to subspheroidal, 2.8–3.2 μm long, with smooth walls, borne in radiate heads.

Typification: Holotype FRR 3425, from cultivated soil, Kumbia, Qld, 1987, J.I. Pitt.

Held as a lyophilised culture in the FRR culture collection at CSIRO Agriculture and Food, North Ryde, NSW, Australia. Isotype CBS 142004. MycoBank No. 818219.

Etym.: named for Mr Niven Hancock, peanut farmer, Kumbia, Queensland, from whose property this distinctive species was first isolated, and which for a number of years was considered to be its only habitat.

Distinctive features: The type of *Aspergillus* head and the black sclerotia produced unequivocally place this species in *Aspergillus* subgenus *Circumdati* section *Flavi*. Unlike all other species in this section, *A*. *hancockii* produces conidia that are greyish green to olive.

Major metabolites produced include the novel hancockiamides A-F, dehydroterrestric acid and 7-hydroxytrichothecolon, as well as the known metabolites onychocins A and B, speradine F, kojic acid, fumitremorgin A and eupenifeldin.

Isolates examined: FRR 3424, FRR 3425, FRR 3426 from soil, Kumbia, Queensland, 1987, J.I. Pitt.

FRR 5050, CBS 142001, from dried peas, Charlton, Vic, 1997, M. Bull.

FRR 6103, CBS 142002, from roadside soil near Lockhart, NSW, 2003, J.I. Pitt.

FRR 6104, from roadside soil near Urana, NSW, 2003, J.I. Pitt.

## Supporting information

S1 File This file contains supporting information for *Aspergillus hancockii* sp. nov. (PDF)

## References

[1] Pitt JI, Hocking AD. Fungi and Food Spoilage. 3rd ed. New York: Springer; 2009.

[2] Kornerup A, Wanscher JH. Methuen Handbook of Colour. 3rd ed. London: Eyre Methuen; 1978.

[3] Greenfield P, Duesing K, Papanicolaou A, Bauer DC. Blue: correcting sequencing errors using consensus and context. Bioinformatics. 2014; 30: 2723–2732. (http://www.bioinformatics.csiro.au/blue/) 10.1093/bioinformatics/btu368 24919879

[4] Zerbino DR, Birney E. Velvet: algorithms for *de novo* short read assembly using de Bruijn graphs. Genome Res. 2008; 18: 821–829. 10.1101/gr.074492.107 18349386 PMC2336801

[5] Hoff KJ, Stanke M. WebAUGUSTUS–a web service for training AUGUSTUS and predicting genes in eukaryotes. Nucleic Acids Res. 2013; 41: W123–W128. 10.1093/nar/gkt418 23700307 PMC3692069

[6] Yin Y, Mao X, Yang J, Chen X, Mao F, Xu Y. dbCAN: a web resource for automated carbohydrate-active enzyme annotation, Nucleic Acids Res. 2012; 40: W445–451. 10.1093/nar/gks479 22645317 PMC3394287

[7] Tamura K, Nei M. Estimation of the number of nucleotide substitutions in the control region of mitochondrial DNA in humans and chimpanzees. Mol Biol Evol. 1993; 10: 512–526. 8336541 10.1093/oxfordjournals.molbev.a040023

[8] Kumar S, Stecher G, Tamura K. MEGA7: Molecular Evolutionary Genetics version 7.0 for bigger databases. Mol Biol Evol. 2016; 33: 1870–1874. 10.1093/molbev/msw054 27004904 PMC8210823

[9] Weber T, Blin K, Duddela S, Krug D, Kim H-U, Bruccoleri R, et al AntiSMASH 3.0 –a comprehensive resource for the genome mining of biosynthetic gene clusters. Nucleic Acids Res. 2015; 43: W237–243. 10.1093/nar/gkv437 25948579 PMC4489286

[10] Medema MH, Kottmann R, Yilmaz P, Cummings M, Biggins JB, Blin K, et al Minimum information about a biosynthetic gene cluster. Nat Chem Biol. 2015; 11: 625–631. 10.1038/nchembio.1890 26284661 PMC5714517

[11] Busk PK, Lange L. A novel method of providing a library of n-mers or biopolymers. Patent application PCT/EP2012/051099. 2012.

[12] Busk PK, Lange L. Function-based classification of carbohydrate-active enzymes by recognition of short, conserved peptide motifs. Appl Environ Microbiol. 2013; 79:3380–3391. 10.1128/AEM.03803-12 23524681 PMC3648057

[13] Busk PK, Lange M, Pilgaard B, Lange L. Several genes encoding enzymes with the same activity are necessary for aerobic fungal degradation of cellulose in nature. PLoS ONE. 2014; 9: e114138 10.1371/journal.pone.0114138 25461894 PMC4252092

[14] Lacey E, Tennant S. Secondary metabolites: the focus of biodiscovery and perhaps the key to unlocking new depths in taxonomy. Microbiol Aust. 2003; 24: 34–35.

[15] Varga J, Frisvad JC, Samson RA. Two new aflatoxin producing species, and an overview of *Aspergillus* section *Flavi*. Stud Mycol. 2011; 69: 57–80. 10.3114/sim.2011.69.05 21892243 PMC3161756

[16] States JS, Christensen M. *Aspergillus leporis*, a new species related to *A*. *flavus*. Mycologia. 1996; 58: 738–742.

[17] TePaske MR, Gloer JB, Wicklow DT, Dowd PF. Leporin A: an antiinsectan N-alkoxypyridone from the sclerotia of *Aspergillus leporis*. Tetrahedron Lett. 1991; 32: 5687–5690.

[18] Busk PK, Lange L. Classification of fungal and bacterial lytic polysaccharide nonooxygenases. BMG Genomics 2015; 16: 368. 10.1186/s12864-015-1601-6 PMC4424831 25956378

[19] Agger JW, Isaksen T, Várnai A, Vidal-Melgosa S, Willats WGT, Ludwig R, et al Discovery of LPMO activity on hemicelluloses shows the importance of oxidative processes in plant cell wall degradation. Proc Nat Acad Sci USA 2014; 111: 6287–6292. 10.1073/pnas.1323629111 24733907 PMC4035949

[20] Vu VV, Beeson WT, Span EA, Farquhar ER, Marletta MA. A family of starch-active polysaccharide monooxygenases. Proc Natl Acad Sci. USA 2014; 111: 13822–13827. 10.1073/pnas.1408090111 25201969 PMC4183312

[21] Lacey HJ, Vuong D, Pitt JI, Lacey E, Piggott AM. Kumbicins A-D: bis-indolyl benzenoids and benzoquinones from an Australian soil fungus, *Aspergillus kumbius*. Aust J Chem. 2016; 69: 152–160.

[22] Yamazaki M, Suzuki S, Miyaki K. Tremorgenic toxins from *Aspergillus fumigatus*. Chem Pharm Bull. 1971; 19: 1739–1740. 5122678 10.1248/cpb.19.1739

[23] Jacobsen JP, Reffstrup T, Cox RE, Holker JSE, Boll PM. Revision of the structures of the naturally occurring acyl tetronic acids: dehydrocarolic acid, terrestric acid and carlic acid. Tetrahedron Lett. 1978; 12: 1081–1084.

[24] Fujita T, Makishima D, Akiyama K, Hayashi H. New convulsive compounds, brasiliamides A and B, from *Penicillium brasilianum* Batista JV-379. Biosci Biotechnol Biochem. 2002; 66: 1697–1705. 10.1271/bbb.66.1697 12353630

[25] Fujita T, Hayashi H. New brasiliamide congeners, brasiliamides C, D and E, from *Penicillium brasilianum* Batista JV-379. Biosci Biotechnol Biochem. 2004; 68: 820–826. 10.1271/bbb.68.820 15118309

[26] Perez-Victoria I, Martin J, Gonzalez-Menendez V, de Pedro N, El Aouad N, Ortiz-Lopez FJ, et al Isolation and structural elucidation of cyclic tetrapeptides from *Onychocola sclerotica*. J Nat Prod. 2012; 75: 1210–1214. 10.1021/np3000987 22694270

[27] Marfey P. Determination of D-amino acids. II. Use of a bifunctional reagent, 1,5-difluoro-2,4-dinitrobenzene. Carlsberg Res Commun. 1984; 49: 591–596.

[28] Yabuta T. Constitution of kojic acid, a γ-pyrone derivative formed by *Aspergillus oryzae* from carbohydrates. J Chem Soc Trans. 1924; 125: 575–587.

[29] Hu X, Xia Q-W, Zhao Y-Y, Zheng Q-H, Liu Q-Y, Chen L, et al Speradines F-H, three new oxindole alkaloids from the marine-derived fungus *Aspergillus oryzae*. Chem Pharm Bull. 2014a; 62: 942–946. 24966178 10.1248/cpb.c14-00312

[30] Tsuda M, Mugishima T, Komatsu K, Sone T, Tanaka M, Mikami Y, et al Speradine A, a new pentacyclic oxindole alkaloid from a marine-derived fungus *Aspergillus tamarii*. Tetrahedron, 2003; 59: 3227–3230.

[31] Hu X, Xia Q-W, Zhao Y-Y, Zheng Q-H, Liu Q-Y, Chen L, et al Speradines B-E, four novel tetracyclic oxindole alkaloids from the marine-derived fungus *Aspergillus oryzae*. Heterocycles. 2014b; 89: 1662–1669. 10.1248/cpb.c14-00312 24966178

[32] Ma X, Peng J, Wu G, Zhu T, Li G, Gu Q, Li D. Speradines B-D, oxygenated cyclopiazonic acid alkaloids from the sponge-derived fungus *Aspergillus flavus* MXH-X104. Tetrahedron. 2015; 71:3522–3527.

[33] Zhu H-C, Chen C-M, Wang J-P, Li X-N, Wei G-Z, Guo Y, et al Penicamedine A, a highly oxygenated hexacyclic indole alkaloid from *Penicillium camemberti*. Chem Biodiv. 2015; 12: 1547–1553. 10.1002/cbdv.201400412 26460559

[34] Wang D, Bao Y-R, Yang X-X, Meng X-S, Chen G. A new alkaloid from *Penicillium dipodomyicola*. Chem Nat Comp. 2015; 51: 733–735.

[35] Mayerl F, Gao Q, Huang S, Klohr SE, Matson JA, Gustavson DR, et al Eupenifeldin, a novel cytotoxic bistropolone from *Eupenicillium brefeldianum*. J Antibiot (Tokyo). 1993; 47: 1082–1088. 10.7164/antibiotics.46.1082 8360103

[36] Sanchez JF, Somoza AD, Keller NP, Wang CC. Advances in *Aspergillus* secondary metabolite research in the post-genomic era. Nat Prod Rep. 2012; 29: 351–71. 10.1039/c2np00084a 22228366 PMC4568942

[37] Cimermancic P, Medema MH, Claesen J, Kurita K, Brown WLC, Mavrommatis K, et al Insights into secondary metabolism from a global analysis of prokaryotic biosynthetic gene clusters. Cell. 2014; 158: 412–421. 10.1016/j.cell.2014.06.034 25036635 PMC4123684

[38] Cacho RA, Tang Y, Chooi YH. Next-generation sequencing approach for connecting secondary metabolites to biosynthetic gene clusters in fungi. Front Microbiol. 2014; 5: 774 10.3389/fmicb.2014.00774 25642215 PMC4294208

[39] Li SM. Genome mining and biosynthesis of fumitremorgin-type alkaloids in ascomycetes. J Antibiot (Tokyo). 2011; 64: 45–49. 21063425 10.1038/ja.2010.128

[40] Mundt K, Wollinsky B, Ruan HL, Zhu T, Li SM. Identification of the verruculogen prenyltransferase FtmPT3 by a combination of chemical, bioinformatic and biochemical approaches. ChemBioChem. 2012; 13: 2583–2592. 10.1002/cbic.201200523 23109474

[41] Gao X, Chooi YH, Ames BD, Wang P, Walsh CT, Tang Y. Fungal indole alkaloid biosynthesis: genetic and biochemical investigation of the tryptoquialanine pathway in *Penicillium aethiopicum*. J Am Chem Soc. 2011; 133: 2729–2741. 10.1021/ja1101085 21299212 PMC3045477

[42] Zhang S, Schwelm A, Jin H, Collins LJ, Bradshaw RE. A fragmented aflatoxin-like gene cluster in the forest pathogen *Dothistroma septosporum*. Fungal Genet Biol. 2007; 44: 1342–54. 10.1016/j.fgb.2007.06.005 17683963

[43] Marui J, Yamane N, Ohashi-Kunihiro S, Ando T, Terabayashi Y, Sano M, et al Kojic acid biosynthesis in *Aspergillus oryzae* is regulated by a Zn(II)(2)Cys(6) transcriptional activator and induced by kojic acid at the transcriptional level. J Biosci Bioeng. 2011; 112: 40–43. 10.1016/j.jbiosc.2011.03.010 21514215

[44] Seshime Y, Juvvadi PR, Tokuoka M, Koyama Y, Kitamoto K, Ebizukay, Fujii I. Functional expression of the *Aspergillus flavus* PKS-NRPS hybrid CpaA involved in the biosynthesis of cyclopiazonic acid. Bioorg Med Chem Lett. 2009; 19: 3288–3292. 10.1016/j.bmcl.2009.04.073 19410456

[45] Kato N, Tokuoka M, Shinohara Y, Kawatani M, Uramoto M, Seshime Y, et al Genetic safeguard against mycotoxin cyclopiazonic acid production in *Aspergillus oryzae*. ChemBioChem. 2011; 12: 1376–1382. 10.1002/cbic.201000672 21608094

[46] Tokuoka M, Kikuchi T, Shinohara Y, Koyama A, Iio S-I, Kobuta T, et al Cyclopiazonic acid biosynthesis gene cluster gene cpaM is required for speradine A biosynthesis. Biosci Biotechnol Biochem. 2015; 79: 2081–2085. 10.1080/09168451.2015.1065167 26207447

[47] Kimura M, Tokai T, Takahashi-Ando N, Ohsato S, Fujimura M. Molecular and genetic studies of *Fusarium* trichothecene biosynthesis: pathways, genes, and evolution. Biosci Biotechnol Biochem. 2007; 71: 2105–2123. 17827683 10.1271/bbb.70183

[48] McCormick SP, Stanley AM, Stover NA, Alexander NJ. Trichothecenes: from simple to complex mycotoxins. Toxins (Basel). 2011; 3: 802–814. 22069741 10.3390/toxins3070802 PMC3202860

[49] Cardoza RE, Malmierca MG, Hermosa MR, Alexander NJ, McCormick SP, Proctor RH, et al Identification of loci and functional characterization of trichothecene biosynthesis genes in filamentous fungi of the genus *Trichoderma*. Appl Environ Microbiol. 2011; 77: 4867–4877. 10.1128/AEM.00595-11 21642405 PMC3147405

[50] Semeiks J, Borek D, Otwinowski Z, Grishin NV. Comparative genome sequencing reveals chemotype-specific gene clusters in the toxigenic black mold *Stachybotrys*. BMC Genomics 2014; 15: 590 10.1186/1471-2164-15-590 25015739 PMC4117958

[51] Tokai T, Koshino H, Takahashi-Ando N, Sato M, Fujimura M, Kimura M. *Fusarium* Tri4 encodes a key multifunctional cytochrome P450 monooxygenase for four consecutive oxygenation steps in trichothecene biosynthesis. Biochem Biophys Res Commun. 2007; 353: 412–417. 10.1016/j.bbrc.2006.12.033 17188234

[52] McCormick SP, Alexander NJ, Trapp SE, Hohn TM. Disruption of TRI101, the gene encoding trichothecene 3-O-acetyltransferase, from *Fusarium sporotrichioides*. Appl Environ Microbiol. 1999; 65: 5252–5256. 10583973 10.1128/aem.65.12.5252-5256.1999 PMC91713

[53] McCormick SP, Harris LJ, Alexander NJ, Ouellet T, Saparno A, Allard S, Desjardins AE. Tri1 in *Fusarium graminearum* encodes a P450 oxygenase. Appl Environ Microbiol. 2004; 70: 2044–2051. 10.1128/AEM.70.4.2044-2051.2004 15066795 PMC383062

[54] Da Silva JV, Fill TP, da Silva BF, Rodrigues-Fo E. Diclavatol and tetronic acids from *Penicillium griseoroseum*. Nat Prod Res. 2013; 27: 9–16. 10.1080/14786419.2011.647021 22239172

[55] Clutterbuck PW, Raistrick H, Reuter F. Studies in the biochemistry of micro-organisms: the metabolic products of *Penicillium charlesii* G. Smith. III. The molecular constitution of carlic and carlosic acids. Biochem J. 1935; 29: 871–883. 16745739 10.1042/bj0290871 PMC1266565

[56] Shoji J, Sakazaki R, Hattori T, Matsumoto K, Uotani N, Yoshida T. Isolation and characterization of agglomerins A, B, C and D. J Antibiot (Tokyo). 1989; 42: 1729–1733. 2621155 10.7164/antibiotics.42.1729

[57] Terui Y, Sakazaki R, Shoji J. Structures of agglomerins. J Antibiot (Tokyo). 1990; 43: 1245–1253. 2258324 10.7164/antibiotics.43.1245

[58] Bentley R, Bhate DS, Keil JG. Tetronic acid biosynthesis in molds. I. Formation of carlosic and carolic acids in *Penicillium charlesii*. J Biol Chem. 1962; 237: 859–866. 13867384

[59] Yang XL, Awakawa T, Wakimoto T, Abe I. Three acyltetronic acid derivatives: noncanonical cryptic polyketides from *Aspergillus niger* identified by genome mining. ChemBioChem. 2014; 15: 1578–1583. 10.1002/cbic.201402172 25044953

[60] Schwarzer D, Finking R, Marahiel MA. Nonribosomal peptides: from genes to products. Nat Prod Rep. 2003; 20: 275–87. 12828367 10.1039/b111145k

[61] Sussmuth R, Muller J, von Dohren H, Molnar I. Fungal cyclooligomer depsipeptides: from classical biochemistry to combinatorial biosynthesis. Nat Prod Rep. 2011; 28: 99–124. 10.1039/c001463j 20959929

[62] Ali H, Ries MI, Lankhorst PP, van der Hoeven RA, Schouten OL, Noga M, et al A non-canonical NRPS is involved in the synthesis of fungisporin and related hydrophobic cyclic tetrapeptides in *Penicillium chrysogenum*. PLoS One. 2014; 9: e98212 10.1371/journal.pone.0098212 24887561 PMC4041764

[63] Hornbogen T, Riechers S-P, Prinz B, Schultchen J, Lang C, Schmidt S, et al Functional characterization of the recombinant N-methyltransferase domain from the multienzyme enniatin synthetase. ChemBioChem.2007;18:1048–1054. 10.1002/cbic.200700076 17471480

[64] Velkov T, Horne J, Scanlon MJ, Capuano B, Yuriev E, Lawen A. Characterization of the N-methyltransferase activities of the multifunctional polypeptide cyclosporin synthetase. Chem Biol. 2011; 18: 464–475. 10.1016/j.chembiol.2011.01.017 21513883

