# Supplementary material for: Aspergillus hancockii sp. nov., a biosynthetically talented fungus endemic to southeastern Australian soils
Source: PLoS One. 2017 Apr 5;12(4):e0170254. doi: 10.1371/journal.pone.0170254 (PMC5381763; doi:10.1371/journal.pone.0170254)
Supplement: S1 File — (PDF) [file pone.0170254.s001.pdf]

SUPPORTING INFORMATION

Aspergillus hancockii sp. nov., a biosynthetically talented fungus endemic to the soils in southeast Australia

John I. Pitt,[1]* Lene Lange,[2] Alastair E. Lacey,[3] Daniel Vuong,[3] David J. Midgley,[1]
Heather J. Lacey,[3] Paul Greenfield,[1] Mark I. Bradbury,[1] Ernest Lacey,[3]
Peter K. Busk,[2] Bo Pilgaard,[2] Yit Heng Chooi[4] and Andrew M. Piggott[5]*

[1] *Food and Nutrition Flagship, Commonwealth Scientific and Industrial Research Organisation, North Ryde, NSW 2113, Australia*

[2] *Department of Chemical and Biochemical Engineering, Technical University of Denmark, 2800 Lyngby, Denmark*

[3] *Microbial Screening Technologies, Smithfield, NSW 2164, Australia*

[4] *School of Chemistry and Biochemistry, University of Western Australia, Crawley, WA 6009, Australia*

[5] *Department of Chemistry and Biomolecular Sciences, Macquarie University, NSW 2109, Australia*

Table of Contents

General Experimental Details 3

Table S1. Recipes for microbiological media 4

Table S2. Full list of carbohydrate active enzymes functionally annotated by PPR/Hotpep analysis of the *Aspergillus hancockii* FRR 3425 genome. 5

Table S3. ^{1}H (600 MHz) and ^{13}C (150 MHz) NMR data for fumitremorgin A in DMSO-d_6 14
Table S4. ^{1}H (600 MHz) and ^{13}C (150 MHz) NMR data for onychocin A in DMSO-d_6 15
Table S5. ^{1}H (600 MHz) and ^{13}C (150 MHz) NMR data for onychocin B in DMSO-d_6 16
Table S6. ^{1}H (600 MHz) and ^{13}C (150 MHz) NMR data for eupenifeldin in $CDCl_3$ 17
Table S7. ^{1}H (600 MHz) and ^{13}C (150 MHz) NMR data for kojic acid in DMSO-d_6 18
Table S8. ^{1}H (600 MHz) and ^{13}C (150 MHz) NMR data for 7-hydroxytrichothecolon in DMSO-d_6 19
Table S9. ^{1}H (600 MHz) and ^{13}C (150 MHz) NMR data for speradine F in DMSO-d_6 20
Table S10. ^{1}H (600 MHz) and ^{13}C (150 MHz) NMR data for dehydroterrestric acid in DMSO-d_6 21

Table S11. LCMS analysis of *Aspergillus hancockii* 40

Figure S1. HPLC traces of the methanolic extracts of *Aspergillus hancockii* FRR3425 grown on various media for 21 days at 24 °C. 9

Figure S2. Overlaid HPLC traces of the methanolic extracts of *Aspergillus hancockii* FRR3425 grown on various media for 21 days at 24 °C 10

Figure S3. HPLC and UV-vis spectra of the major secondary metabolites present in a methanolic extract of *Aspergillus hancockii* FRR3425 grown on hydrated rice grain for 21 days at 24 °C 11

Corresponding authors.
JIP: Ph: +61-2-9490-8525; Fax: +61-2-9490-8499; Email: John.Pitt@csiro.au
AMP: Ph: +61-2-9850-8251; Fax: +61-2-9850-8313; Email: andrew.piggott@mq.edu.au

Figure S4. HPLC-ESI(+/–)MS analysis of the methanolic extract of *Aspergillus hancockii* FRR3425 grown on hydrated rice grain for 21 days at 24 °C. 12

Figure S5. Isolation scheme for major secondary metabolites of *Aspergillus hancockii* after cultivation on hydrated rice grain for 21 days at 24 °C 13

Figure S6. ^{1}H NMR spectrum (600 MHz, DMSO-d_6) of fumitremorgin A 22
Figure S7. ^{13}C NMR spectrum (150 MHz, DMSO-d_6) of fumitremorgin A 23

Figure S8. ^{1}H NMR spectrum (600 MHz, DMSO-d_6) of onychocin A 24
Figure S9. ^{13}C NMR spectrum (150 MHz, DMSO-d_6) of onychocin A 25

Figure S10. ^{1}H NMR spectrum (600 MHz, DMSO-d_6) of onychocin B 26
Figure S11. ^{13}C NMR spectrum (150 MHz, DMSO-d_6) of onychocin B 27

Figure S12. ^{1}H NMR spectrum (600 MHz, $CDCl_3$) of eupenifeldin 28
Figure S13. ^{13}C NMR spectrum (150 MHz, $CDCl_3$) of eupenifeldin 29

Figure S14. ^{1}H NMR spectrum (600 MHz, DMSO-d_6) of kojic acid 30
Figure S15. ^{13}C NMR spectrum (150 MHz, DMSO-d_6) of kojic acid 31

Figure S16. ^{1}H NMR spectrum (600 MHz, DMSO-d_6) of 7-hydroxytrichothecolon 32
Figure S17. ^{13}C NMR spectrum (150 MHz, DMSO-d_6) of 7-hydroxytrichothecolon 33

Figure S18. ^{1}H NMR spectrum (600 MHz, DMSO-d_6) of speradine F 34
Figure S19. ^{13}C NMR spectrum (150 MHz, DMSO-d_6) of speradine F 35

Figure S20. ^{1}H NMR spectrum (600 MHz, DMSO-d_6) of dehydroterrestric acid 36
Figure S21. ^{13}C NMR spectrum (150 MHz, DMSO-d_6) of dehydroterrestric acid 37

Figure S22. Marfey's Analysis of onychocin A 38
Figure S23. Marfey's Analysis of onychocin B 39

General Experimental Details

NMR spectra were obtained on a Bruker Avance DRX600 spectrometer in the solvents indicated and referenced to residual signals in the deuterated solvents. High resolution electrospray ionisation mass spectra (HRESIMS) were obtained on an Agilent G6538A Q-TOF mass spectrometer by direct infusion. Electrospray ionisation mass spectra (ESIMS) were acquired on an Agilent 1260 UHPLC coupled to an Agilent G6130B single quadrupole mass detector. Chiroptical measurements ($[\alpha]_D$) were obtained on a JASCO P-1000 polarimeter in a 100 × 10 mm cell. UV-vis spectra were acquired in MeCN on a Varian Cary 300 spectrophotometer in a 10 × 10 mm quartz cell.

Analytical HPLC was performed on a gradient Shimadzu HPLC system comprising a LC-10AT VP gradient chromatograph, SPD-M10A VP diode array detector and SCL-10A VP system controller. The column was an Alltima C_{18} "rocket" format column (100 Å, 53 × 7 mm, 3 µm; Grace Discovery, Deerfield, IL, USA) eluted with a 3 mL/min gradient of 10–100% MeCN/H_2O (+ 0.01% TFA) over 7 min.

Preparative HPLC was performed on a gradient Shimadzu HPLC system comprising two LC-8A preparative liquid pumps with a static mixer, SPD-M10AVP diode array detector and SCL-10AVP system controller with a standard Rheodyne injection port. The column was a Hypersil C_{18} spring column (150 × 50 mm, 5 µm; Grace Discovery) eluted isocratically at 60 mL/min. Further purification was undertaken using a semi-preparative Alltima C_{18} column 150 × 22 mm, 5 µm; Grace Discovery) eluted isocratically at 10 mL/min.

Analytical cultivation of the fungal strains was undertaken on a range of liquid, agar and grain-based media. The cultivations were sub-sampled (1 g) and extracted with methanol (2 mL) for a minimum of 1 h on a wrist shaker, centrifuged (13,000 rpm for 3 min) and analysed by HPLC. The major metabolites were analysed using COMET. Metabolites not previously observed were accessioned and targeted for preparative cultivation, purification, characterisation and structure elucidation.

Table S1. Recipes for microbiological media

**Glycerol Casein Agar (CGA)**	
Ingredient	*Quantity*
Glycerol	30 g
Casein peptone (Amyl)	2 g
K_2HPO_4	1 g
NaCl	1 g
$MgSO_4.7H_2O$	0.5 g
Trace element solution*	5 mL
Deionised water	1000 mL
Bacteriological agar (Amyl)	20 g
Autoclave	
***Trace element solution**	
$CaCl_2.2H_2O$	3 g
$FeC_6O_7H_5$	1 g
$MnSO_4$	0.2 g
$ZnCl_2$	0.1 g
CuSO4.5H_2O	0.025 g
$Na_2B_4O_7.10H_2O$	0.02 g
$CoCl_2$	0.004 g
$Na_2MoO_4.2H_2O$	0.01 g
Deionised water	1000 mL
Filter sterilize	

**Czapeks Agar (CZA)**	
Ingredient	*Quantity*
Czapeks Dox Media (Oxoid)	99.88 g
Deionised water	2200 mL

**Malt Extract Agar (MEA)**	
Ingredient	*Quantity*
Bacteriological peptone (Difco)	3 g
Malt Extract (Amyl)	60 g
Bacteriological glucose (Amyl)	60 g
Distilled water	1000 mL
Adjust pH to 5.5	
Bacteriological agar (Amyl)	20 g
Autoclave	

**Yeast Extract Sucrose Agar (YES)**	
Ingredient	*Quantity*
Yeast Extract (Difco) (g)	20 g
Sucrose (Amyl) (g)	150 g
Bacteriological Agar (Amyl) (g)	20 g
Deionised water (mL)	1000 mL
Autoclave	

NOTE: For liquid media, agar is omitted from the recipe

Table S2. Full list of carbohydrate active enzymes functionally annotated by PPR/Hotpep analysis of the *Aspergillus hancockii* FRR 3425 genome.

**Substrate**	**EC number**	**Enzyme function**	**No. of genes**	**Enzyme families representing function**					
Auxiliary Activities	1.*.*.*	Glucooligosaccharide oxidase Chitooligosaccharide oxidase	2	2 AA3	0	0	0	0	0
	1.1.3.*	Glucose oxidase	2	2 AA3	0	0	0	0	0
	1.1.3.4	Galactose oxidase	2	2 AA3	0	0	0	0	0
	1.1.3.9	Pyranose oxidase	3	3 AA5	0	0	0	0	0
	1.1.3.10	Alcohol oxidase	1	1 AA3	0	0	0	0	0
	1.1.3.13	Cellobiose dehydrogenase (acceptor)	1	1 AA3	0	0	0	0	0
	1.1.99.18	Pyranose dehydrogenase (acceptor)	5	3 AA3	2 AA8	0	0	0	0
	1.1.99.29		5	5 AA3	0	0	0	0	0
	1.10.3.*	Laccase	8	8 AA1	0	0	0	0	0
	1.10.3.2	LPMO	8	8 AA1	0	0	0	0	0
	-		19	14AA9	5AA11	0	0	0	0
		**Sum**	**56**						
Polysaccharide lyases	4.2.2.*		3	3 PL4	0	0	0	0	0
	4.2.2.2	Pectate lyase	4	1 PL1	3 PL3	0	0	0	0
	4.2.2.10	Pectin lyase	8	8 PL1	0	0	0	0	0
	4.2.2.14	Glucuronan lyase	1	1 PL20	0	0	0	0	0
		**Sum**	**16**						
Carbohydrate esterases	3.1.1.*		5	1 CE0	3 CE12	1 CE15	0	0	0
	3.1.1.6	Acetylesterase	2	2 CE16	0	0	0	0	0
	3.1.1.11	Pectinesterase	3	3 CE8	0	0	0	0	0
	3.1.1.72	Acetylxylan esterase	4	1 CE1	1 CE2	2 CE4	0	0	0
	3.1.1.74	Cutinase	4	4 CE5	0	0	0	0	0
	3.5.1.*		1	1 CE9	0	0	0	0	0
	3.5.1.25	*N*-Acetylglucosamine-6-phosphate deacetylase	1	1 CE9	0	0	0	0	0
	4.2.2.*		3	3 PL4	0	0	0	0	0
	4.2.2.2	Pectate lyase	4	1 PL1	3 PL3	0	0	0	0
		**Sum**	**27**						

Glycosyl transferases	2.4.1.		14	7 GT1	1 GT22	1 GT24	2 GT32	1 GT50	2 GT57
	2.4.1.1	Phosphorylase	1	1 GT35	0	0	0	0	0
	2.4.1.11	Glycogen(starch) synthase	1	1 GT3	0	0	0	0	0
	2.4.1.15	α,α-Trehalose-phosphate synthase (UDP forming)	6	6 GT20	0	0	0	0	0
	2.4.1.16	Chitin synthase	8	8 GT2	0	0	0	0	0
	2.4.1.34	1,3-β-Glucan synthase	1	1 GT48	0	0	0	0	0
	2.4.1.80	Ceramide glucosyltransferase	1	1 GT21	0	0	0	0	0
	2.4.1.83	Dolichyl-phosphate β-D-Mannosyltransferase	1	1 GT2	0	0	0	0	0
	2.4.1.109	Dolichyl-phosphate-mannose-protein mannosyltransferase	3	3 GT39	0	0	0	0	0
	2.4.1.129	Peptidoglycan glycosyltransferase	1	1 GT51	0	0	0	0	0
	2.4.1.131	GDP-Man:Man3GlcNAc2-PP-dolichol α-1,2-mannosyltransferase	4	3 GT15	0	0	0	0	0
	2.4.1.142	Chitobiosyldiphosphodolichol β-mannosyltransferase	1	1 GT33	0	0	0	0	0
	2.4.1.183	α-1,3-Glucan synthase	3	3 GH13	0	0	0	0	0
	2.4.1.186	Glycogenin glucosyltransferase	1	1 GT8	0	0	0	0	0
	2.4.1.198	Phosphatidylinositol N-ccetylglucosaminyltransferase	1	1 GT4	0	0	0	0	0
	2.4.1.255	Protein O-GlcNAc transferase	1	1 GT41	0	0	0	0	0
	2.4.1.256	Dolichyl-P-Glc:Glc2Man9GlcNAc2-PP-Dolichol α-1,2-glucosyltransferase	1	1 GT59	0	0	0	0	0
	2.4.1.257	GDP-Man:Man2GlcNAc2-PP-Dolichol_alpha-1,6-mannosyltransferase	4	3 GT32	0	0	0	0	0
	2.4.1.258	Dolichyl-P-Man:Man5GlcNAc2-PP-Dolichol_alpha-1,3-mannosyltransferase	1	1 GT58	0	0	0	0	0
	2.4.1.259	Dolichyl-P-Man:Man6GlcNAc2-PP-dolichol_alpha-1,2-mannosyltransferase	1	1 GT22	0	0	0	0	0
	2.4.1.260	Dolichyl-P-Man:Man7GlcNAc2-PP-dolichol_alpha-1,6-mannosyltransferase	1	1 GT22	0	0	0	0	0
	2.4.99.*		1	1 GT29	0	0	0	0	0
	2.4.99.18	dolichyl-diphosphooligosaccharide-protein_glycotransferase	1	1 GT66	0	0	0	0	0
	3.5.1.*		1	1 CE9	0	0	0	0	0
		**Sum**	**59**						

Glycoside hydrolases	2.4.1.5	Dextransucrase	1	1 GH70	0	0	0	0	0
	2.4.1.18	1,4-α-Glucan branching enzyme	1	1 GH13	0	0	0	0	0
	2.4.1.25	4-α-Glucanotransferase	2	1 GH13	1 GH133	0	0	0	0
	2.4.1.183	α-1,3-Glucan synthase	3	3 GH13	0	0	0	0	0
	3.2.1.*		9	2 GH131	1 GH132	1 GH43	2 GH88	1GH93	0
	3.2.1.1	α-Amylase	4	4 GH13	0	0	0	0	0
	3.2.1.3	Glucan 1,4-α-glucosidase	3	3 GH15	0	0	0	0	0
	3.2.1.4	Cellulase	5	1 GH12	4 GH5	0	0	0	0
	3.2.1.6	Endo-1,3(4)-β-glucanase	2	2 GH16	0	0	0	0	0
	3.2.1.8	Endo-1,4-β-xylanase	17	10 GH10	6 GH11	0	0	0	0
	3.2.1.14	Chitinase	17	17 GH18	0	0	0	0	0
	3.2.1.15	Polygalacturonase	9	9 GH28	0	1 GH43	0	0	0
	3.2.1.17	Lysozyme	2	1 GH24	1 GH25	0	0	0	0
	3.2.1.20	α-Glucosidase	7	4 GH13	3 GH31	0	0	0	0
	3.2.1.21	β-Glucosidase	21	4 GH1	17 GH3	0	0	0	0
	3.2.1.22	α-Galactosidase	3	2 GH27	1 GH36	0	0	0	0
	3.2.1.23	β-Galactosidase	4	1 GH2	4 GH35	0	0	0	0
	3.2.1.24	α-Mannosidase	1	1 GH38	0	0	0	0	0
	3.2.1.25	β-Mannosidase	3	3 GH2	0	0	0	0	0
	3.2.1.26	β-Fructofuranosidase	3	3 GH32	0	0	0	0	0
	3.2.1.28	α,α-Trehalase	3	2 GH37	1 GH65	0	0	0	0
	3.2.1.37	Xylan 1,4-β-xylosidase	7	2 GH3	3 GH43	0	0	0	0
	3.2.1.39	Glucan endo-1,3-β-D-glucosidase	4	2 GH16	2 GH17	0	0	0	0
	3.2.1.40	α-L-Rhamnosidase	5	5 GH78	0	2 GH5	0	0	0
	3.2.1.45	Glucosylceramidase	1	1 GH5	0	1 GH81	0	0	0
	3.2.1.52	β-N-Acetylhexosaminidase	4	3 GH20	1 GH3	0	0	0	0
	3.2.1.55	α-N-Arabinofuranosidase	12	2 GH43	4 GH51	5 GH62	0	0	0
	3.2.1.58	Glucan 1,3-β-glucosidase	3	2 GH5	1 GH55	0	0	0	0
	3.2.1.59	Glucan endo-1,3-α-glucosidase	11	11 GH71	0	1 GH54	5 GH62	0	0
	3.2.1.63	1,2-α-L-Fucosidase	2	2 GH95	0	0	0	0	0
	3.2.1.67	Galacturan 1,4-α-galacturonidase	2	2 GH28	0	0	0	0	0
	3.2.1.75	glucan endo-1,6-β-glucosidase	1	1 GH5	0	0	0	0	0
	3.2.1.78	Mannan endo-1,4-β-mannosidase	3	1 GH26	2 GH5	0	0	0	0
	3.2.1.80	Fructan β-fructosidase	4	4 GH32	0	0	0	0	0
	3.2.1.89	Arabinogalactan endo-1,4-galactanase	1	1 GH53	0	0	0	0	0
	3.2.1.91	Cellulose 1,4-β-cellobiosidase (non-reducing end)	1	1 GH6	0	0	0	0	0
	3.2.1.96	mannosyl-glycoprotein_endo-beta-N-acetylglucosaminidase	1	1 GH18	0	0	0		

	3.2.1.99	arabinan_endo-1,5-alpha-L-arabinanase	5	5 GH43	0	0	0		
	3.2.1.106	mannosyl-oligosaccharide_glucosidase	1	1 GH63	0	0	0		
	3.2.1.113	mannosyl-oligosaccharide_1,2-alpha-mannosidase	5	5 GH47	0	0	0		
	3.2.1.131	xylan_alpha-1,2-glucuronosidase	1	1 GH67	0	0	0		
	3.2.1.132	chitosanase	5	5 GH75	0	0	0		
	3.2.1.145	galactan_1,3-beta-galactosidase	1	1 GH43	0	0	0		
	3.2.1.149	beta-primeverosidase	2	2 GH5	0	0	0		
	3.2.1.151	xyloglucan-specific_endo-beta-1,4-glucanase	1	1 GH12	0	0	0		
	3.2.1.164	galactan_endo-1,6-beta-galactosidase	1	1 GH5	0	0	0		
	3.2.1.165	exo-1,4-beta-D-glucosaminidase	1	1 GH2	0	0	0		
	3.2.1.171	rhamnogalacturonan_hydrolase	2	2 GH28	0	0	0		
	3.2.1.174	rhamnogalacturonan_rhamnohydrolase	1	1 GH28	0	0	0		
	3.2.1.176	cellulose_1,4-beta-cellobiosidase_(reducing_end)	3	3 GH7	0	0	0		
	3.2.1.177	alpha-D-xyloside_xylohydrolase	2	2 GH31	0	0	0		
	3.2.1.96	Mannosyl-glycoprotein endo-β-N-acetylglucosaminidase	1	1 GH18	0	0	0	0	0
	3.2.1.99	Arabinan endo-1,5-α-L-arabinanase	5	5 GH43	0	0	0	0	0
	3.2.1.106	Mannosyl-oligosaccharide glucosidase	1	1 GH63	0	0	0	0	0
	3.2.1.113	Mannosyl-oligosaccharide 1,2-α-mannosidase	5	5 GH47	0	0	0	0	0
	3.2.1.131	Xylan α-1,2-glucuronosidase	1	1 GH67	0	0	0	0	0
	3.2.1.132	Chitosanase	5	5 GH75	0	0	0	0	0
	3.2.1.145	Galactan 1,3-β-galactosidase	1	1 GH43	0	0	0	0	0
	3.2.1.149	β-Primeverosidase	2	2 GH5	0	0	0	0	0
	3.2.1.151	Xyloglucan-specific endo-β-1,4-glucanase	1	1 GH12	0	0	0	0	0
	3.2.1.164	Galactan endo-1,6-β-galactosidase	1	1 GH5	0	0	0	0	0
	3.2.1.165	Exo-1,4-β-D-glucosaminidase	1	1 GH2	0	0	0	0	0
	3.2.1.171	Rhamnogalacturonan hydrolase	2	2 GH28	0	0	0	0	0
	3.2.1.174	Rhamnogalacturonan rhamnohydrolase	1	1 GH28	0	0	0	0	0
	3.2.1.176	Cellulose 1,4-β-cellobiosidase (reducing end)	3	3 GH7	0	0	0	0	0
	3.2.1.177	α-D-Xyloside xylohydrolase	2	2 GH31	0	0	0	0	0
		**Sum**	**213**						
		**Total sum**	**371**						

Media 1: CGA Agar

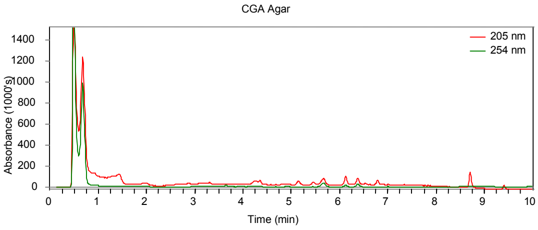

Media 2: CZA Agar

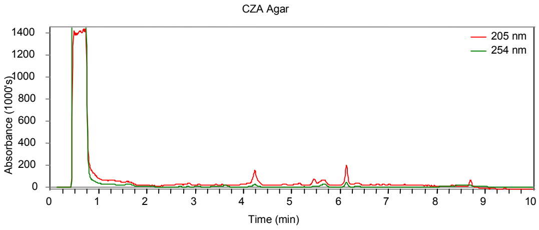

Media 3: MEA Agar

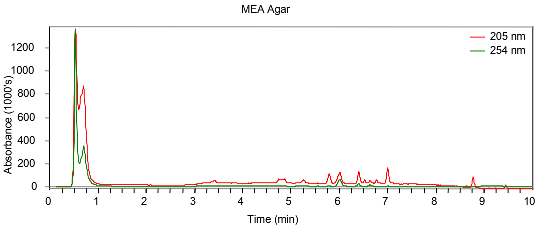

Media 4: YES Agar

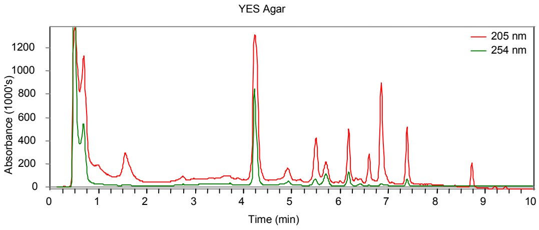

Media 5: Cracked wheat

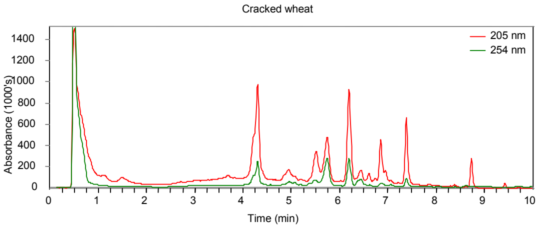

Media 6: Rice (Basmati)

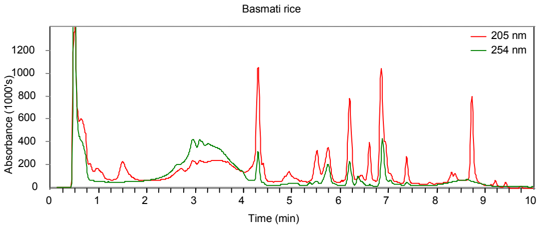

Media 7: Rice (Jasmine)

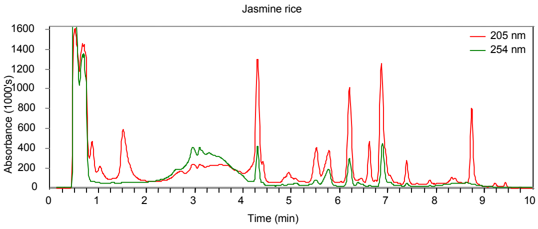

Media 8: Pearl barley

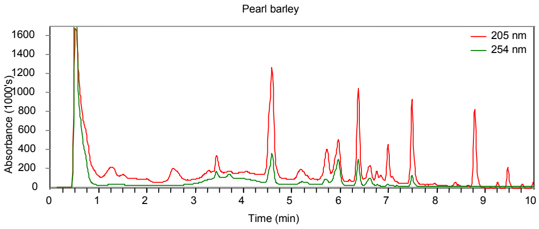

Media 9: CGA Liquid

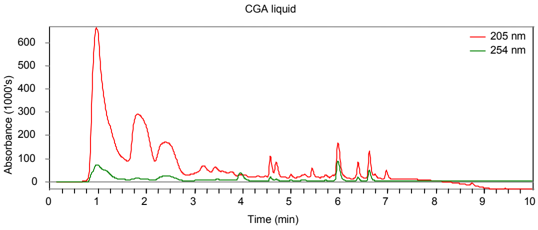

Media 10: CZA Liquid

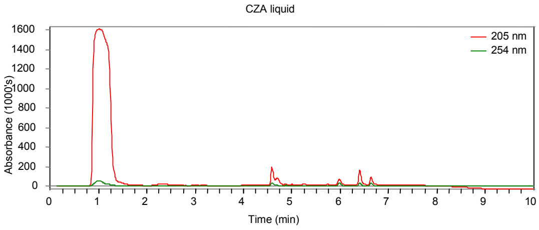

Media 11: MEA Liquid

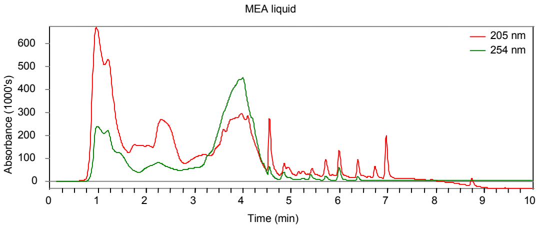

Media 12: YES Liquid

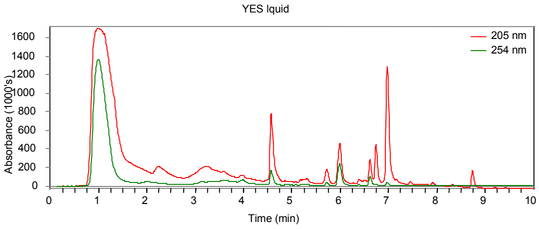

Figure S1. HPLC traces of the methanolic extracts of *Aspergillus hancockii* FRR3425 grown on various media for 21 days at 24 °C.

205 nm

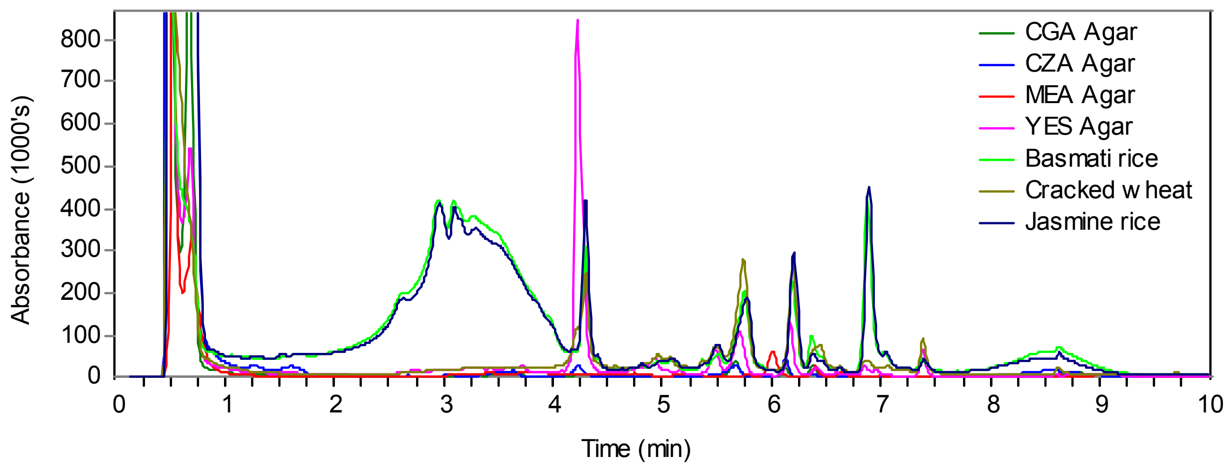

254 nm

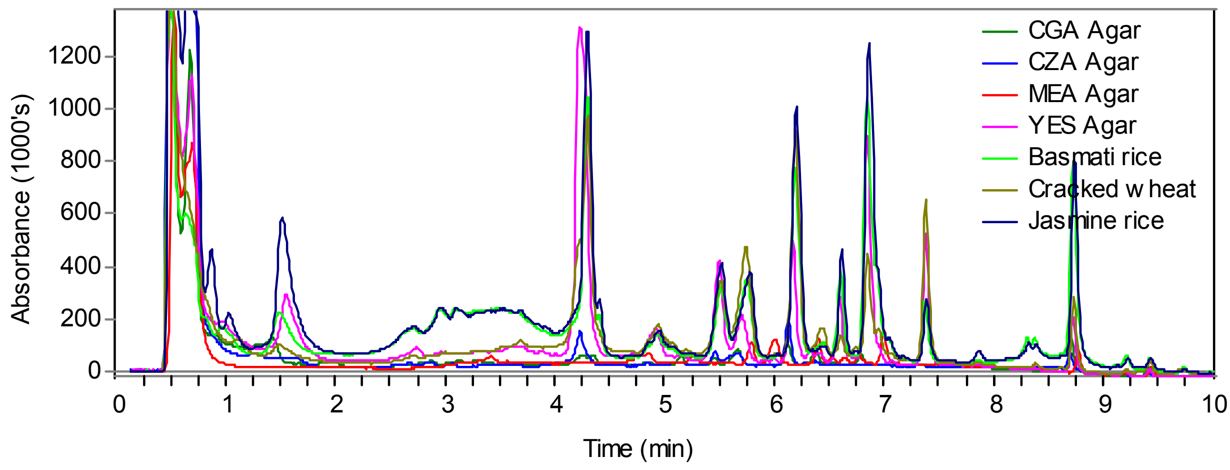

Figure S2. Overlaid HPLC traces of the methanolic extracts of *Aspergillus hancockii* FRR3425 grown on various media for 21 days at 24 °C

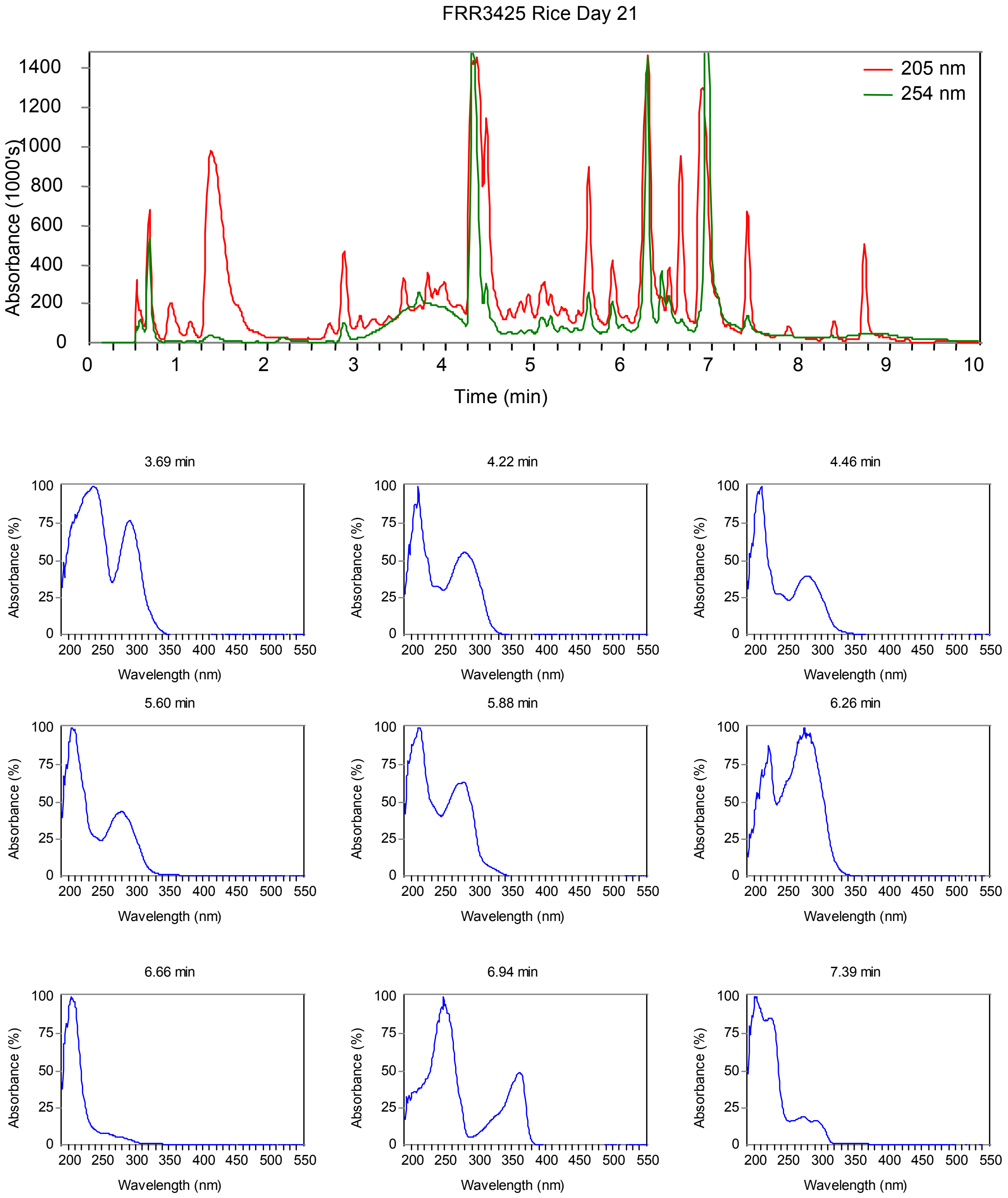

Figure S3. HPLC and UV-vis spectra of the major secondary metabolites present in a methanolic extract of *Aspergillus hancockii* FRR3425 grown on hydrated rice grain for 21 days at 24 °C.

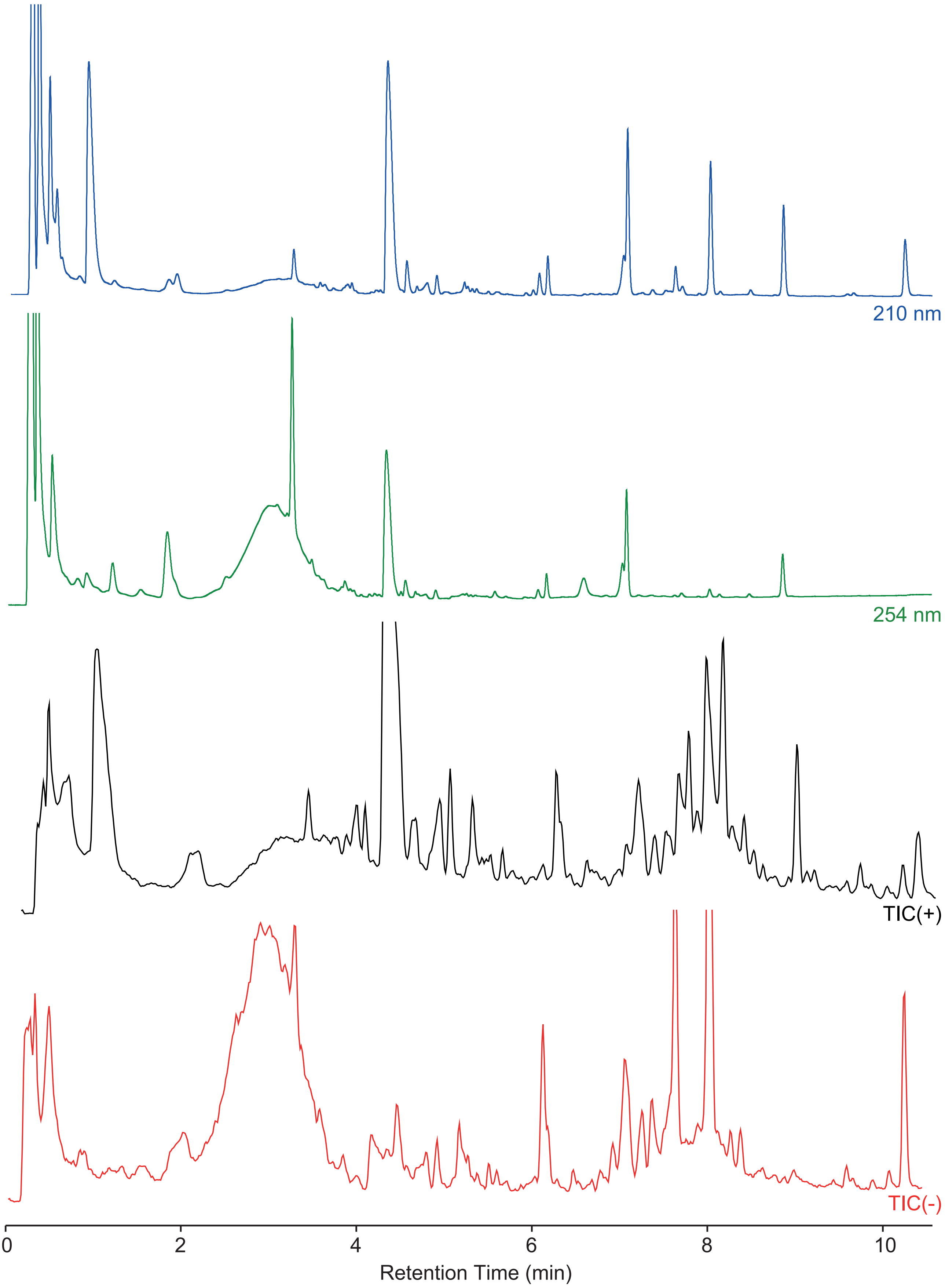

Figure S4. HPLC-ESI(+/–)MS analysis of the methanolic extract of *Aspergillus hancockii* FRR3425 grown on hydrated rice grain for 21 days at 24 °C.

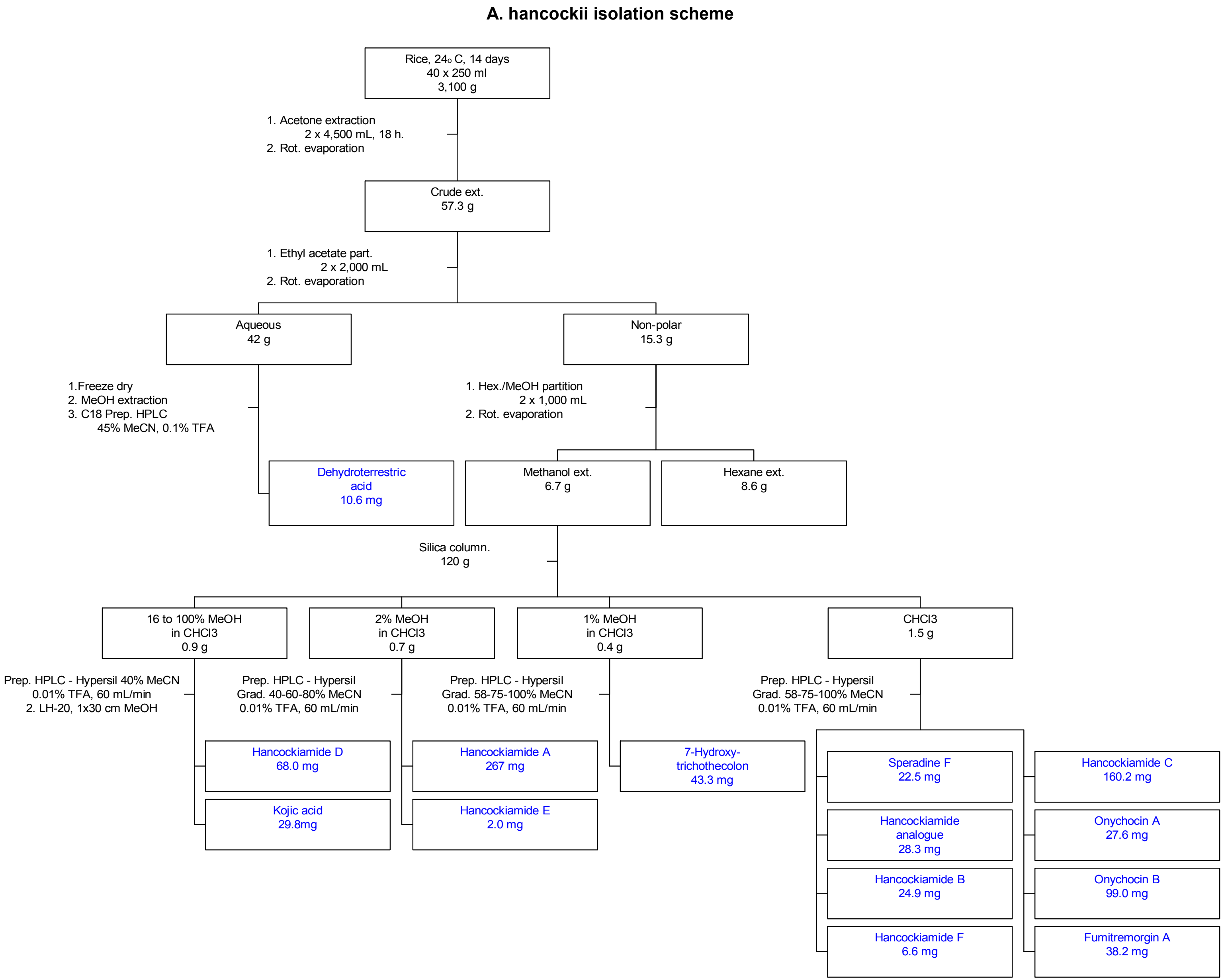

Figure S5. Isolation scheme for major secondary metabolites of *Aspergillus hancockii* after cultivation on hydrated rice grain for 21 days at 24 °C

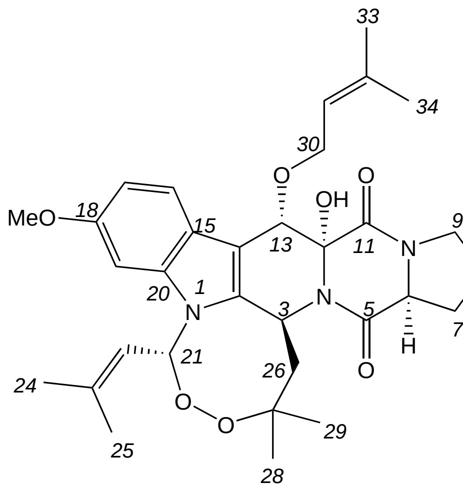

fumitremorgin A

Table S3. ^{1}H (600 MHz) and ^{13}C (150 MHz) NMR data for fumitremorgin A in DMSO-d_6

Pos.	δ_H, mult (J in Hz)	δ_C	HMBC	COSY	ROESY
2		131.2			
3	5.96, d (10.4)	47.0	2, 5, 12, 14, 26, 27	26a/b	
5		171.3			
6	4.61, dd (9.0, 7.5)	58.5	5, 7, 8	7a/b	
7a	2.28, m	28.7	6, 8, 9	6, 7b, 8	
7b	1.98, m		5, 6, 8, 9	6, 7a, 8	
8	1.89, m	22.5		7a/b, 9a/b	
9a	3.48, ddd (11.1, 10.1, 7.1)	45.4	6, 7, 8, 11	8, 9b	
9b	3.39, ddd (11.1, 8.0, 3.1)		6, 7, 8, 11	8, 9a	
11		165.5			
12		84.5			
13	5.14, s	73.4	2, 11, 12, 14, 15, 30		26b, 30a/b
14		107.1			
15		120.4			
16	7.55, d (8.6)	120.4	14, 18, 20	17	30b, 31
17	6.73, dd (8.6, 2.2)	109.0	15, 18, 19	16	18-OMe
18		155.4			
19	6.75, d (2.2)	93.8	15, 17, 18, 20		18-OMe
20		135.8			
21	6.81, d (8.3)	85.2	2, 22, 23	22	25
22	4.96, dm (8.3, 1.2)	118.2	24, 25	21, 24, 25	24
23		142.9			
24	1.69, d (1.2)	25.3	22, 23, 25	22	22
25	1.98, d (1.2)	18.5	22, 23. 24	22	21
26a	1.77, d (13.6)	50.7	2, 3, 27, 28, 29	3, 26b	
26b	1.59, dd (13.6, 10.4)		2, 3, 27, 28, 29	3, 26a	13
27		81.6			
28	1.55, s	24.2	26, 27, 29		29
29	0.95, s	26.7	26, 27, 28		28
30a	4.90, dd (11.1, 6.6)	68.6	13, 31, 32	30b, 31	13
30b	4.55, dd (11.1, 6.8)		13, 31, 32	30a, 31	13, 16, 34
31	5.52, ddm (6.8, 6.6, 1.2)	122.3	33, 34	30a/b, 33, 34	16, 33
32		134.4			
33	1.76, s	25.6	31, 32, 34	31	31
34	1.73, s	18.2	31, 32, 33	31	30b
12-OH	6.32, br s				
18-OMe	3.75, s	55.2	18		17, 19

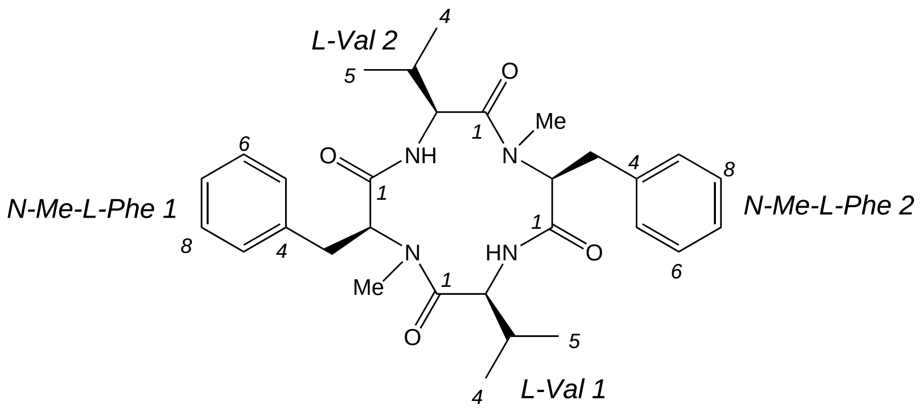

onychocin A

Table S4. ^{1}H (600 MHz) and ^{13}C (150 MHz) NMR data for onychocin A in DMSO-d_6

Pos.	δ_H, mult (J in Hz)	δ_C	HMBC	COSY
***N*-Me-L-Phe 1 / *N*-Me-L-Phe 2**				
1		169.8		
2	4.21, dd (11.7, 3.4)	62.1	1, 3, 4	3a/b
3a	3.48, dd (14.6, 3.4)	33.8	1, 2, 4, 5/9	2
3b	2.94, dd (14.6, 11.7)		1, 2, 4, 5/9	2
4		137.9		
5/9	7.18, d (7.3)	128.2	3, 7	6/8
6/8	7.31, dd (7.5, 7.3)	128.6	4	5/9, 7
7	7.21, t (7.5)	126.6	5/9	6/8
N-Me	2.64, s	30.3	1, 2	
**L-Val 1 / L-Val 2**				
1		170.7		
2	4.02, dd (9.0, 7.2)	54.8	1, 3, 4, 5	3, NH
3	1.91, m	28.8	1, 2, 4, 5	2, 4, 5
4	0.77, d (6.7)	18.1	2, 3, 5	3, 5
5	0.66, d (6.7)	20.3	2, 3, 4	3, 4
NH	8.06, d (9.0)		1, 2	2

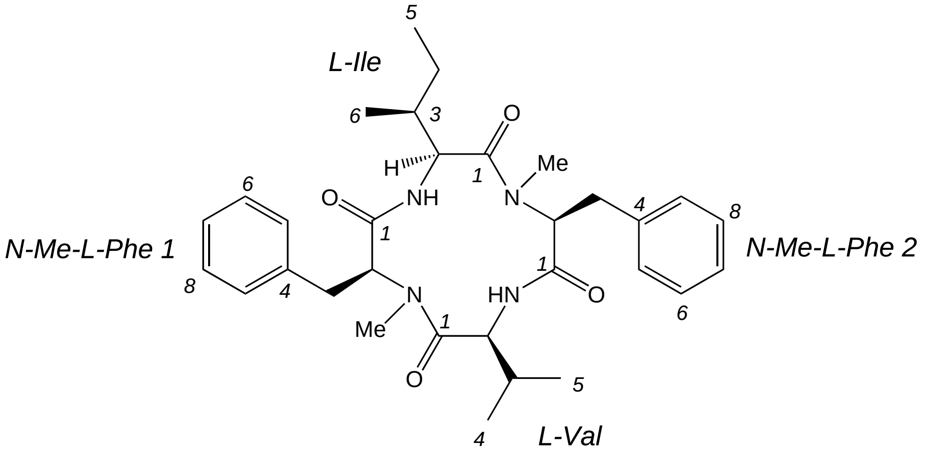

onychocin B

Table S5. ^{1}H (600 MHz) and ^{13}C (150 MHz) NMR data for onychocin B in DMSO-d_6

Pos.	δ_H, mult (J in Hz)	δ_C	HMBC	COSY
***N*-Me-L-Phe 1**				
1		169.8		
2	4.21, dd (11.6, 3.6)	62.2	1, 3, 4	3a/b
3a	3.47, dd (14.6, 3.6)	33.8	1, 2, 4, 5/9	2
3b	2.94, dd (14.6, 11.6)		1, 2, 4, 5/9	2
4		137.9		
5/9	7.17, d (7.4)	128.2	3, 7	6/8
6/8	7.31, dd (7.4, 7.4)	128.6	4	5/9, 7
7	7.21, t (7.4)	126.6	5/9	6/8
N-Me	2.64, s	30.3	1, 2	
**L-Val**				
1		170.7		
2	4.02, dd (8.9, 7.7)	54.8	1, 3, 4, 5	3, NH
3	1.91, m	28.9	1, 2, 4, 5	2, 4, 5
4	0.77, d (6.7)	18.1	2, 3, 5	3, 5
5	0.66, d (6.7)	20.3	2, 3, 4	3, 4
NH	8.05, d (8.9)		1, 2	2
***N*-Me-L-Phe 2**				
1		169.8		
2	4.19, dd (11.6, 3.6)	62.1	1, 3, 4	3a/b
3a	3.47, dd (14.6, 3.6)	33.8	1, 2, 4, 5/9	2
3b	2.94, dd (14.6, 11.6)		1, 2, 4, 5/9	2
4		137.9		
5/9	7.17, d (7.4)	128.2	3, 7	6/8
6/8	7.31, dd (7.4, 7.4)	128.6	4	5/9, 7
7	7.21, t (7.4)	126.6	5/9	6/8
N-Me	2.63, s	30.3	1, 2	
**L-Ile**				
1		170.6		
2	4.08, dd (8.9, 7.7)	54.2	1, 3, 4, 6	3, NH
3	1.64, m	35.6	1, 2, 4, 5, 6	4a/b, 6
4a	1.41, m	24.2	2, 3, 5, 6	3, 4b, 5
4b	0.88, m		2, 3, 5, 6	3, 4a, 5
5	0.77, t (7.0)	11.7	3, 4	4a/b
6	0.64, d (6.6)	16.5	2, 3, 4	3
NH	8.07, d (8.9)		1, 2	

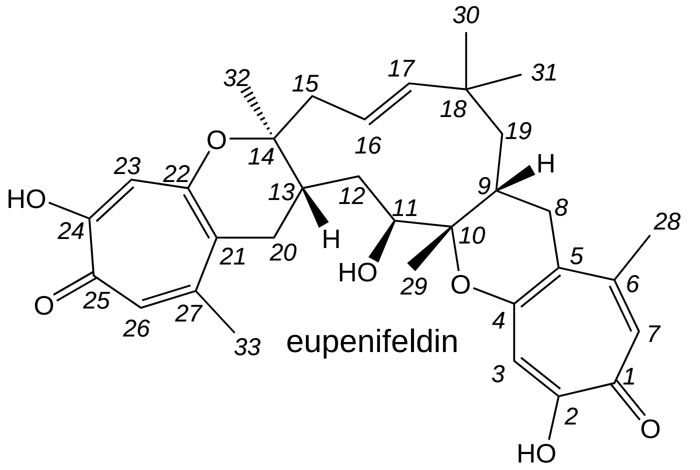

Table S6. ^{1}H (600 MHz) and ^{13}C (150 MHz) NMR data for eupenifeldin in $CDCl_3$

Pos.	δ_H, mult (J in Hz)	δ_C	HMBC	COSY	ROESY
1		172.4			
2		163.2			
3	6.99, s	113.5	1, 2, 4, 5		
4		160.1			
5		119.6			
6		152.0			
7	7.24, s	125.4	1, 2, 5, 6, 28		28
8a	2.88, dd (17.2, 5.2)	34.3	4, 5, 6, 9, 19	8b, 9	29
8b	2.40, d (17.2)		4, 5, 6, 9, 19	8a, 9	19b, 30
9	1.84, m	31.8	5, 8, 10, 18, 19	8a/b, 13, 19a/b	17, 29, 30
10		82.1			
11	4.23, d (11.2)	70.7	9, 10, 12, 13, 29	12a/b	16, 19a, 32
12a	2.24, m	30.0	10, 11, 13, 14, 20	11, 12b	17
12b	1.55, m		10, 11, 13, 14, 20	11, 12a, 13	29
13	2.21, m	41.4	11, 12, 14, 20, 32	12b, 20a/b	15b
14		80.9			
15a	2.77, ddd (13.4, 4.3, 1.9)	46.2	13, 14, 16, 17, 32	15b, 16	16
15b	2.54, dd (13.4, 10.9)		13, 14, 16, 17, 32	15a, 16	13, 17
16	5.69, ddd (15.9, 10.9, 4.3)	125.7	14, 15, 17, 18	15a/b, 17	11, 15a, 19a, 31, 32
17	5.92, dd (15.9, 1.9)	144.1	15, 16, 18, 19, 30, 31	16	9, 12a, 15b, 30
18		34.9			
19a	1.79, d (14.6)	46.5	8, 9, 17, 18, 30, 31	9, 19b	11, 16, 31
19b	0.78, dd (14.6, 4.4)		8, 9, 17, 18, 30, 31	9, 19b	8b, 31
20a	3.44, dd (19.4, 14.2)	33.0	13, 21, 22	13, 20b	32
20b	2.44, d (19.4)		13, 21, 22	13, 20a	13
21		124.3			
22		161.6			
23	7.06, s	114.7	21, 22, 24, 25		
24		163.5			
25		171.0			
26	7.26, s	124.5	21, 24, 25, 27, 33		33
27		151.4			
28	2.40, s	27.4[a]	5, 6, 7		7
29	1.16, s	16.0	9, 10, 11		8a, 9, 12b
30	1.11, s	27.1	17, 18, 19, 31		8b, 9, 17
31	1.08, s	29.6	17, 18, 19, 30		16, 19a/b
32	1.42, s	19.3	13, 14, 15		11, 16, 20a
33	2.45, s	27.4[a]	21, 26, 27		26

[a] Overlapping resonances

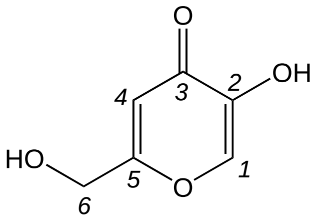

kojic acid

Table S7. ^{1}H (600 MHz) and ^{13}C (150 MHz) NMR data for kojic acid in DMSO-d_6

Pos.	δ_H, mult (J in Hz)	δ_C	HMBC	COSY
1	8.01, s	139.2	2, 3, 5	
2		145.7		
2-OH	9.01, br s			
3		173.9		
4	6.32, t (0.8)	109.8	2, 3, 5, 6	
5		168.0		
6	4.28, d (4.1)	59.4	4, 5	6-OH
6-OH	5.65, t (4.1)		5, 6	6

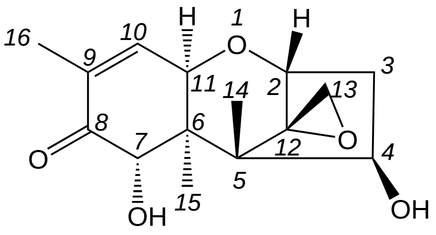

7-hydroxytrichothecolon

Table S8. ^{1}H (600 MHz) and ^{13}C (150 MHz) NMR data for 7-hydroxytrichothecolon in DMSO-d_6

Pos.	δ_H, mult (J in Hz)	δ_C	HMBC	COSY	ROESY
2	3.73, d (4.9)	79.7	4, 5, 11, 12, 13	3b	3a/b, 13a
3a	2.37, dd (14.9, 7.7)	39.2	2, 5, 12	3b, 4	2, 3b, 4, 11
3b	1.73, ddd (14.9, 4.9, 4.1)		4	2, 3a, 4	2, 3a
4	4.18, dd (7.7, 4.1)	71.3	2, 6, 12	3a/b	3a, 11, 15
5		49.3			
6		47.8			
7	4.64, s	74.7	5, 6, 8, 11, 15		13a/b
8		201.0			
9		134.9			
10	6.48, dq (5.9, 1.5)	137.9	6, 8, 11, 16	11, 16	11, 16
11	3.98, d (5.9)	70.3	2, 5, 6, 7, 9, 10, 15	10	3a, 4, 10, 15
12		65.5			
13a	2.97, d (4.7)	45.3	2, 5, 12	13b	2, 7
13b	2.83, d (4.7)		2, 5, 12	13a	7, 14
14	0.88, s	7.9	4, 5, 6, 12		13b
15	0.74, s	12.3	5, 6, 7, 11		4, 11
16	1.71, s	14.8	8, 9, 10	10	10

Selected 2D NMR correlations:

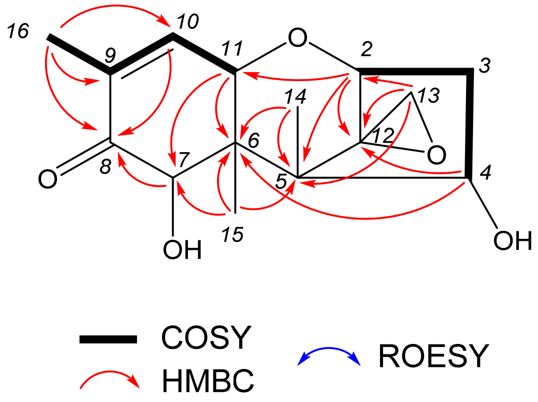

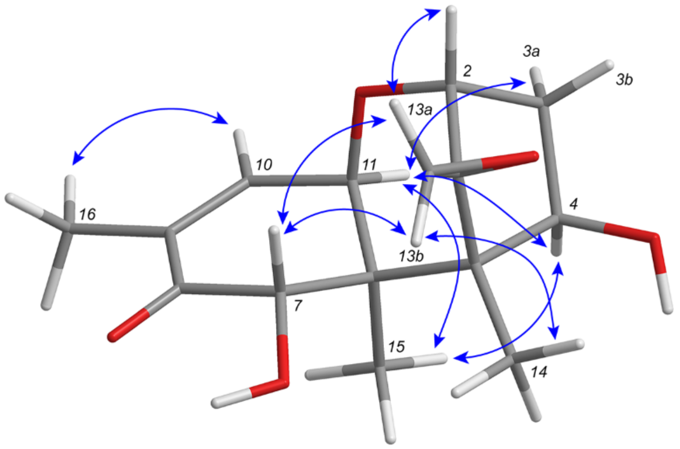

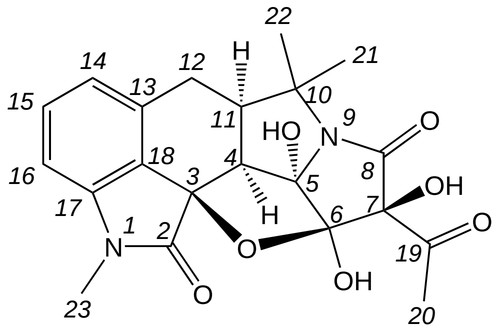

speradine F

Table S9. ^{1}H (600 MHz) and ^{13}C (150 MHz) NMR data for speradine F in DMSO-d_6

Pos.	δ_H, mult (J in Hz)	δ_C	HMBC	COSY	ROESY
2		177.6			
3		82.5			
4	3.21, d (8.4)	53.7	2, 3, 5, 11, 12	11	21
5		102.4			
5-OH	5.36, s		4, 5, 6		6-OH, 21
6		106.9			
6-OH	6.16, s		5, 6, 7		5-OH
7		87.8			
7-OH	6.49, s		6, 7, 8, 19		20
8		169.8			
10		68.4			
11	2.33, ddd (13.8. 8.4, 5.4)	54.4	4, 12, 21, 22	4	21
12a	2.66, dd (13.6, 5.4)	26.7	4, 11, 13, 14, 18	11, 12b	14, 22
12b	2.43, dd (13.8, 13.6)		4, 10, 11, 13, 14, 18	11, 12a	22
13		137.9			
14	6.98, d (7.7)	121.7	12, 15, 16, 18	15	12a
15	7.35, dd (7.8, 7.7)	131.6	13, 14, 16, 17	14, 16	
16	6.94, d (7.8)	107.4	14, 17	15	23
17		142.6			
18		122.0			
19		208.8			
20	2.22, s	26.9	7, 19		7-OH
21	1.45, s	30.0	10, 11, 22		4, 5-OH, 11, 22
22	1.64, s	21.2	10, 11, 21		12a/b, 21
23	3.15, s	26.5	2, 17		16

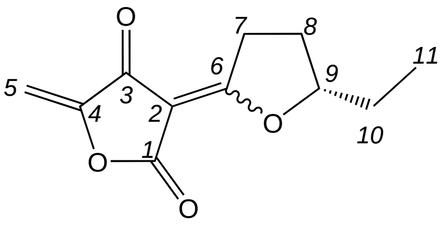

dehydroterrestric acid

Table S10. ^{1}H (600 MHz) and ^{13}C (150 MHz) NMR data for dehydroterrestric acid in DMSO-d_6

Pos.	δ_H, mult (J in Hz) *Z* isomer	δ_C	δ_H, mult (J in Hz) *E* isomer	δ_C	HMBC	COSY
1		162.9		166.6		
2		103.5		103.2		
3		182.2		178.7		
4		151.9		151.6		
5a	5.17, d (2.5)	91.7	5.16, d (2.6)	91.8	3, 4	5b
5b	4.99[a], d (2.5)		5.00[a], d (2.6)		3, 4	5a
6		190.1		189.0		
7a	3.55, ddd (19.9, 9.4, 4.1)	34.5	3.47, ddd (19.9, 9.3, 4.2)	34.3	2, 6, 8, 9	
7b	3.31, ddd (19.9,9.6, 8.4)		3.29, ddd (19.9, 9.6, 8.4)		6, 8, 9	
8a	2.32[b], m	25.9	2.31[b], m	25.8	6, 7, 10	8b, 9
8b	1.81[c], m		1.82[c], m		6, 7, 9, 10	9a, 9
9	5.00[a], m	93.5	5.01[a], m	94.0	6, 7, 10, 11	8a/b, 10a/b
10a	1.80[c], m	26.87	1.80[c], m	26.90	8, 9, 11	9, 10b, 11
10b	1.73[d], m		1.72[d], m		8, 9, 11	9, 10a, 11
11	0.96, t (7.4)	9.41	0.95, t (7.4)	9.37	9, 10	10a/b

[a-d] Overlapping resonances

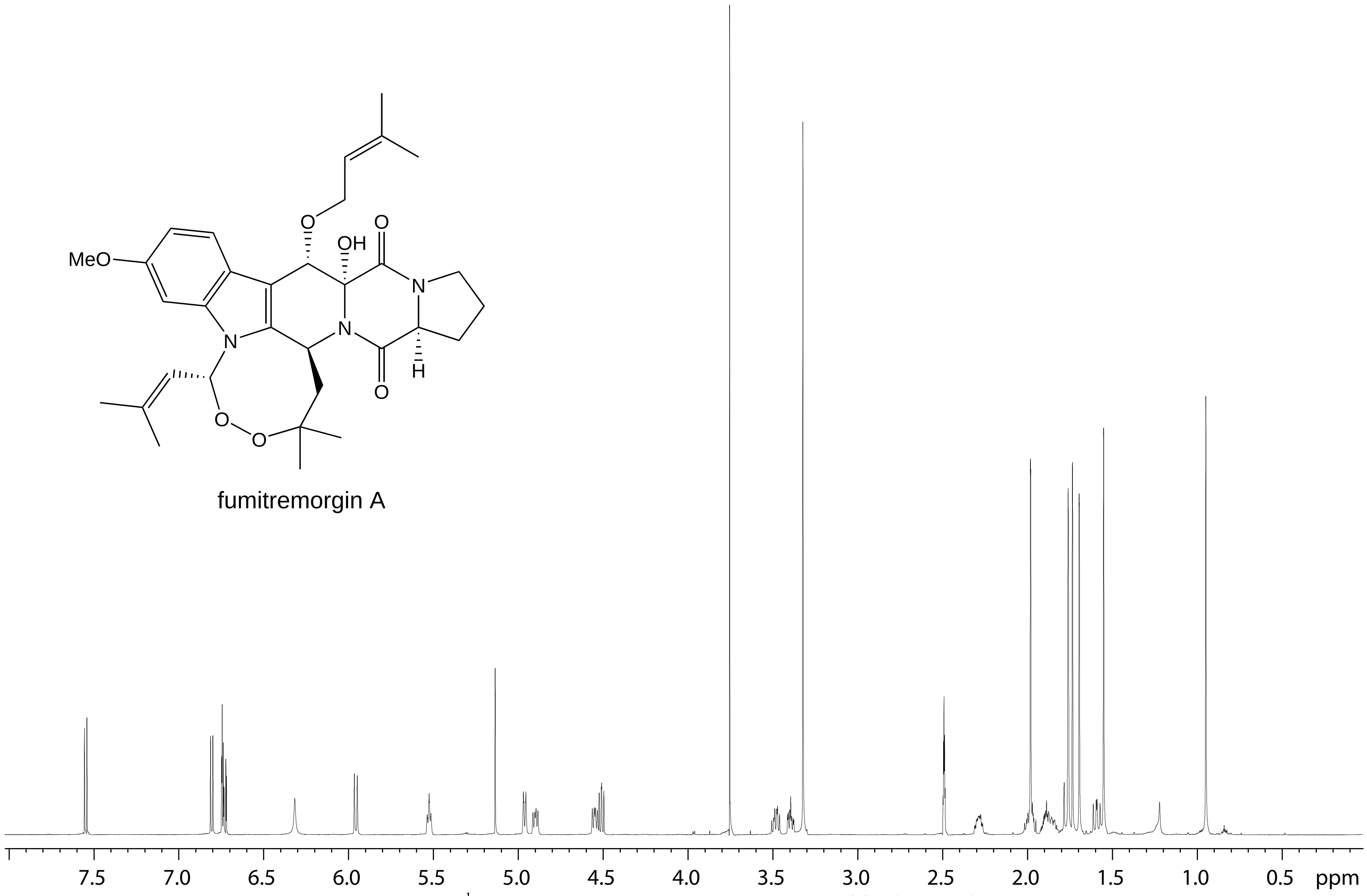

Figure S6. ^{1}H NMR spectrum (600 MHz, DMSO-d_6) of fumitremorgin A

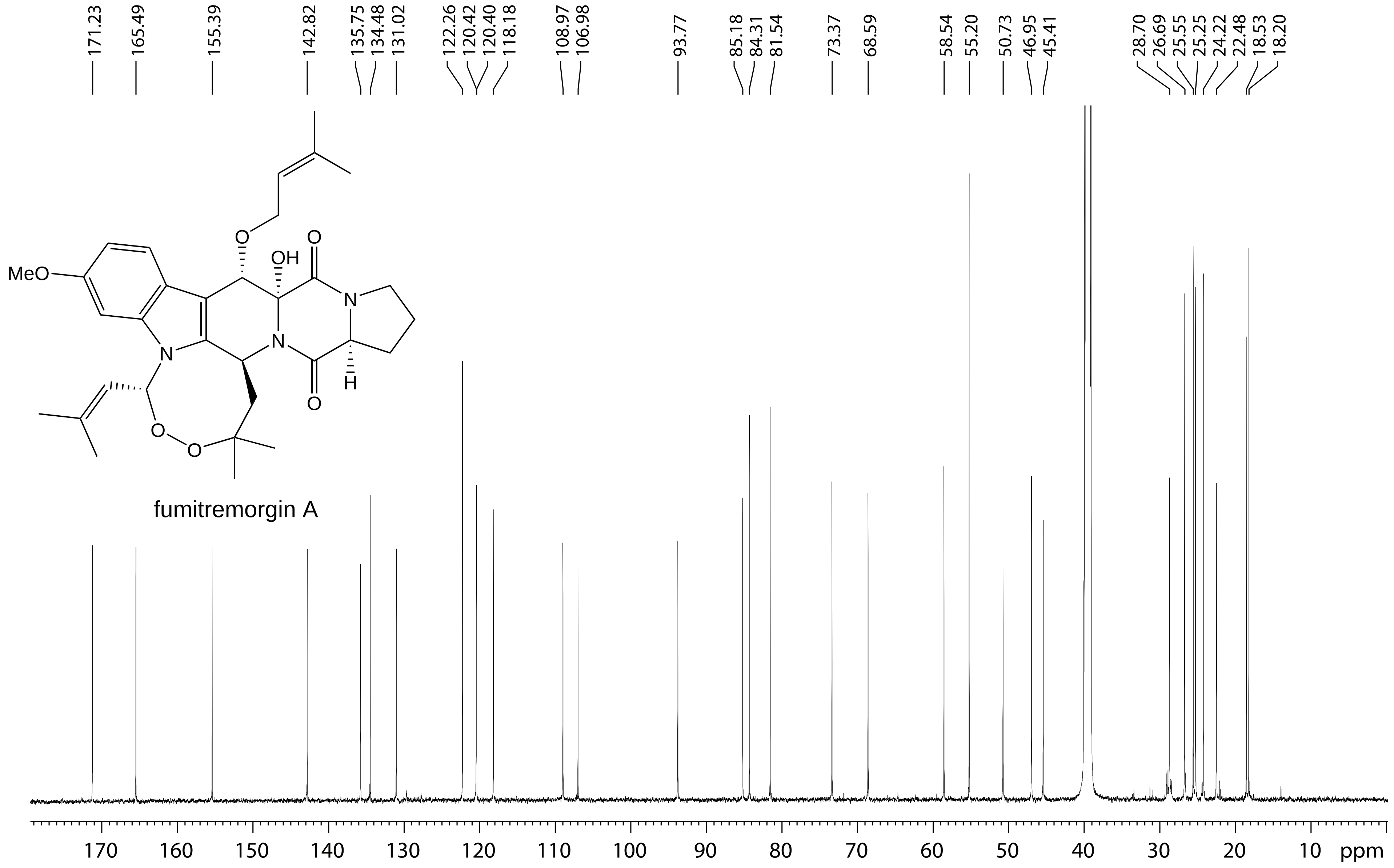

Figure S7. ^{13}C NMR spectrum (150 MHz, DMSO-d_6) of fumitremorgin A

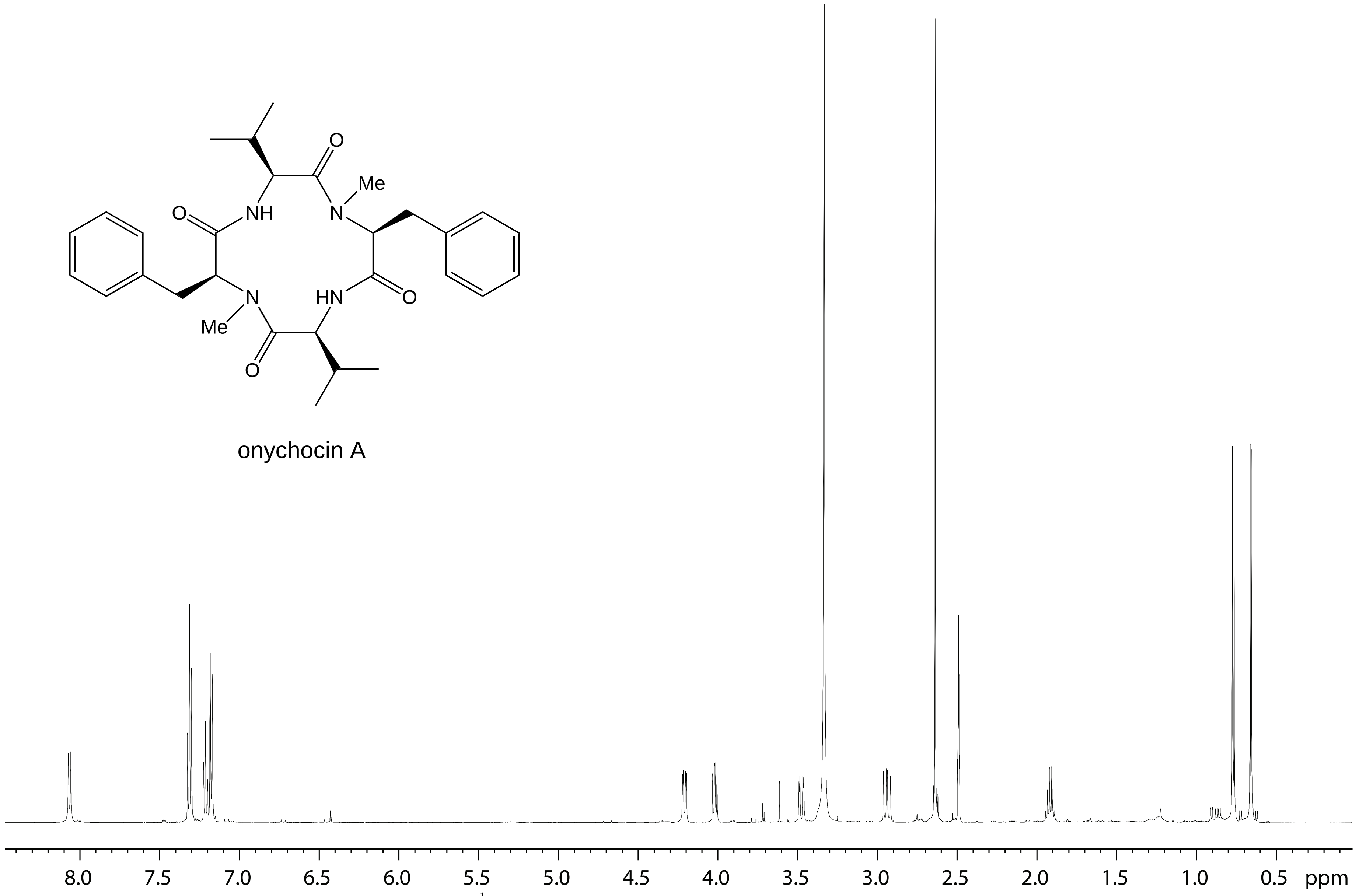

Figure S8. ^{1}H NMR spectrum (600 MHz, DMSO-d_6) of onychocin A

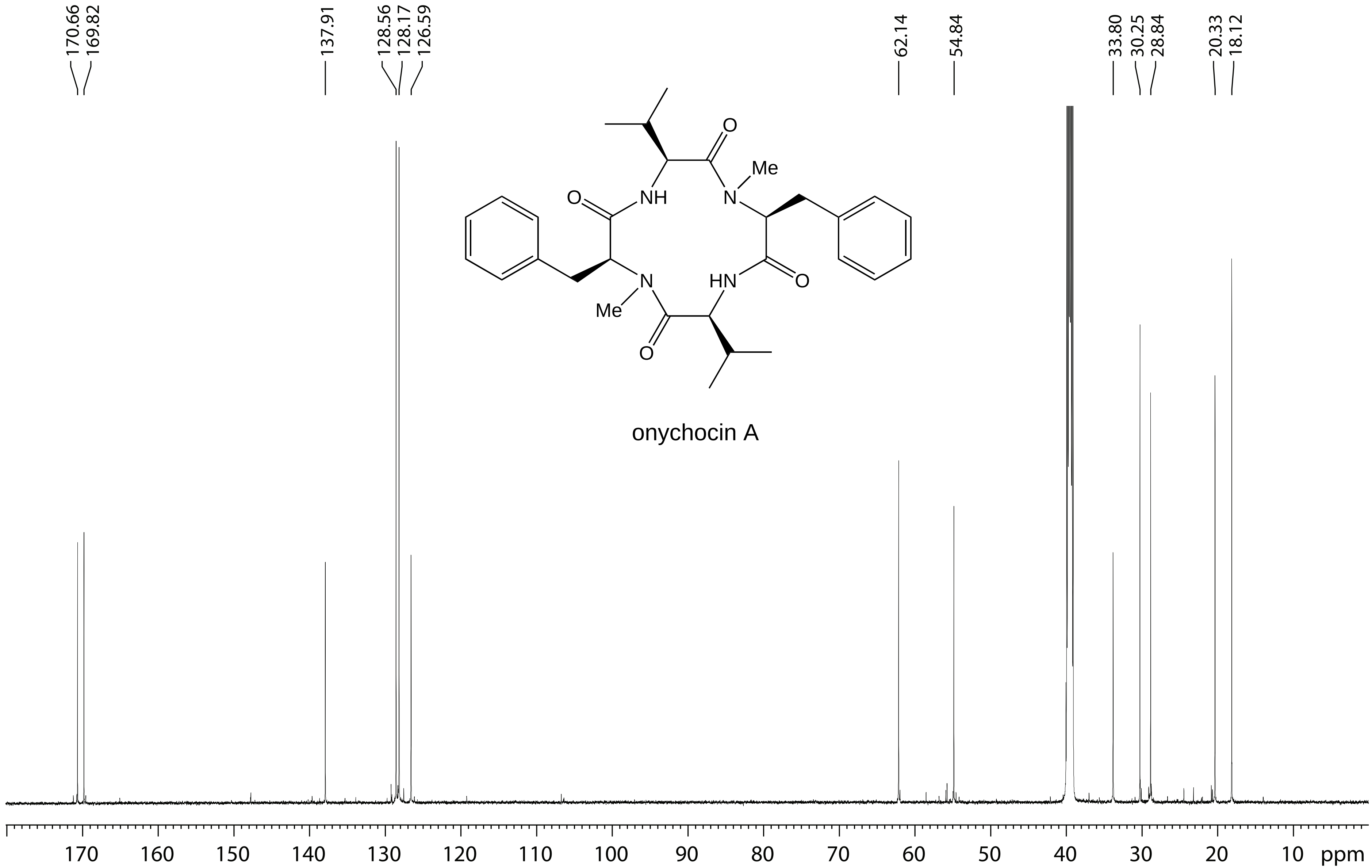

Figure S9. ^{13}C NMR spectrum (150 MHz, DMSO-d_6) of onychocin A

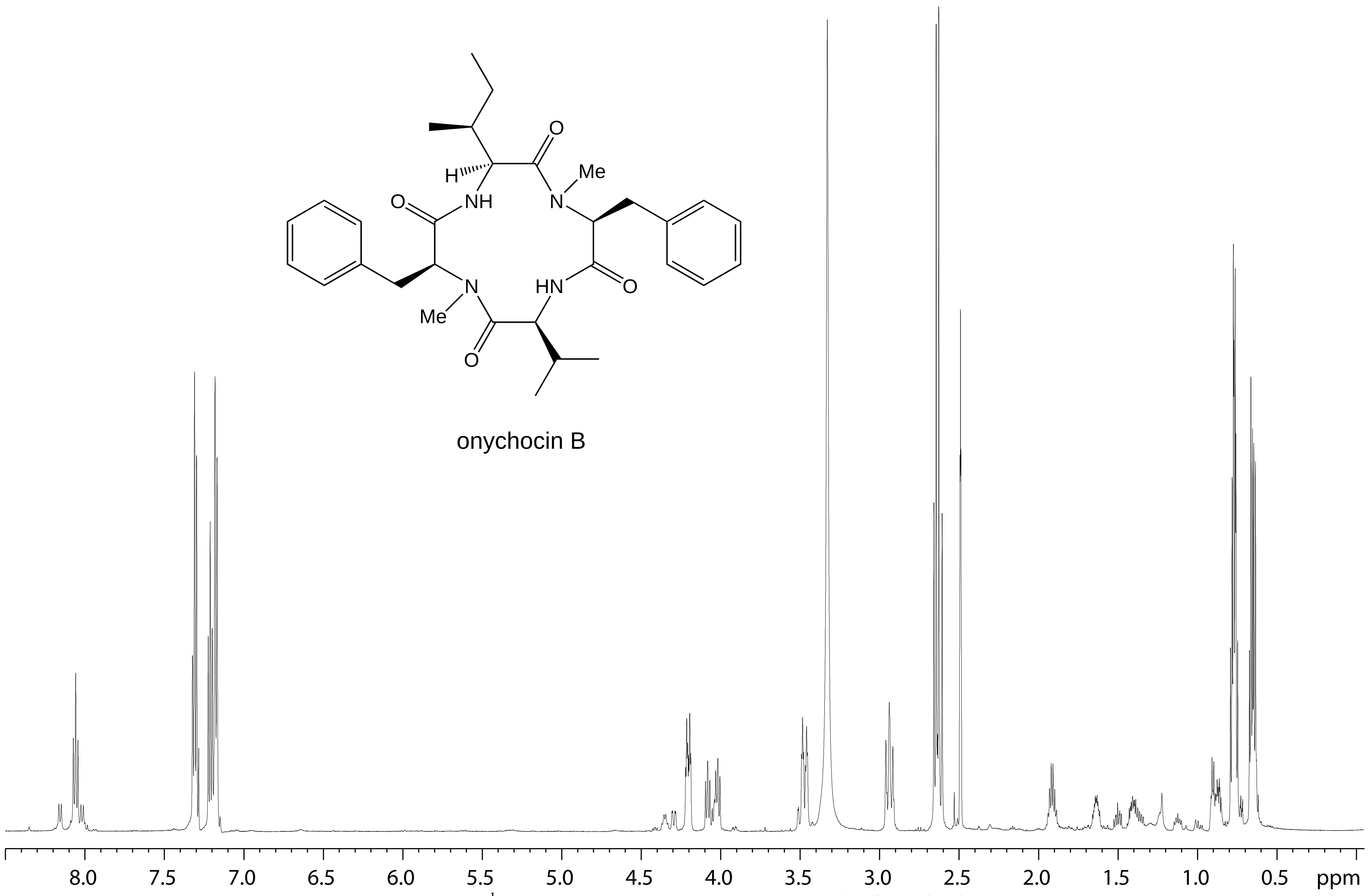

Figure S10. ^{1}H NMR spectrum (600 MHz, DMSO-d_6) of onychocin B

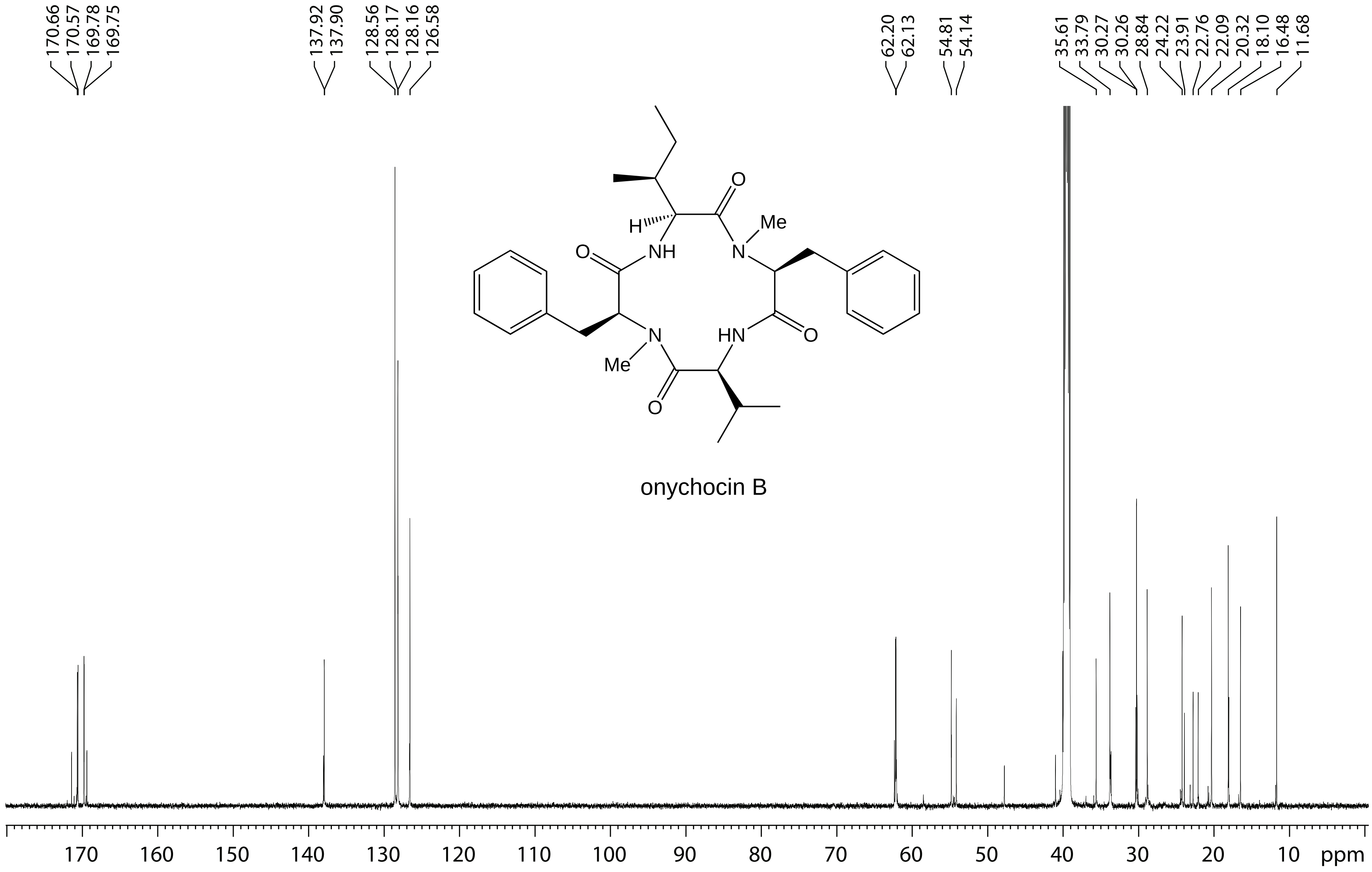

Figure S11. ^{13}C NMR spectrum (150 MHz, DMSO-d_6) of onychocin B

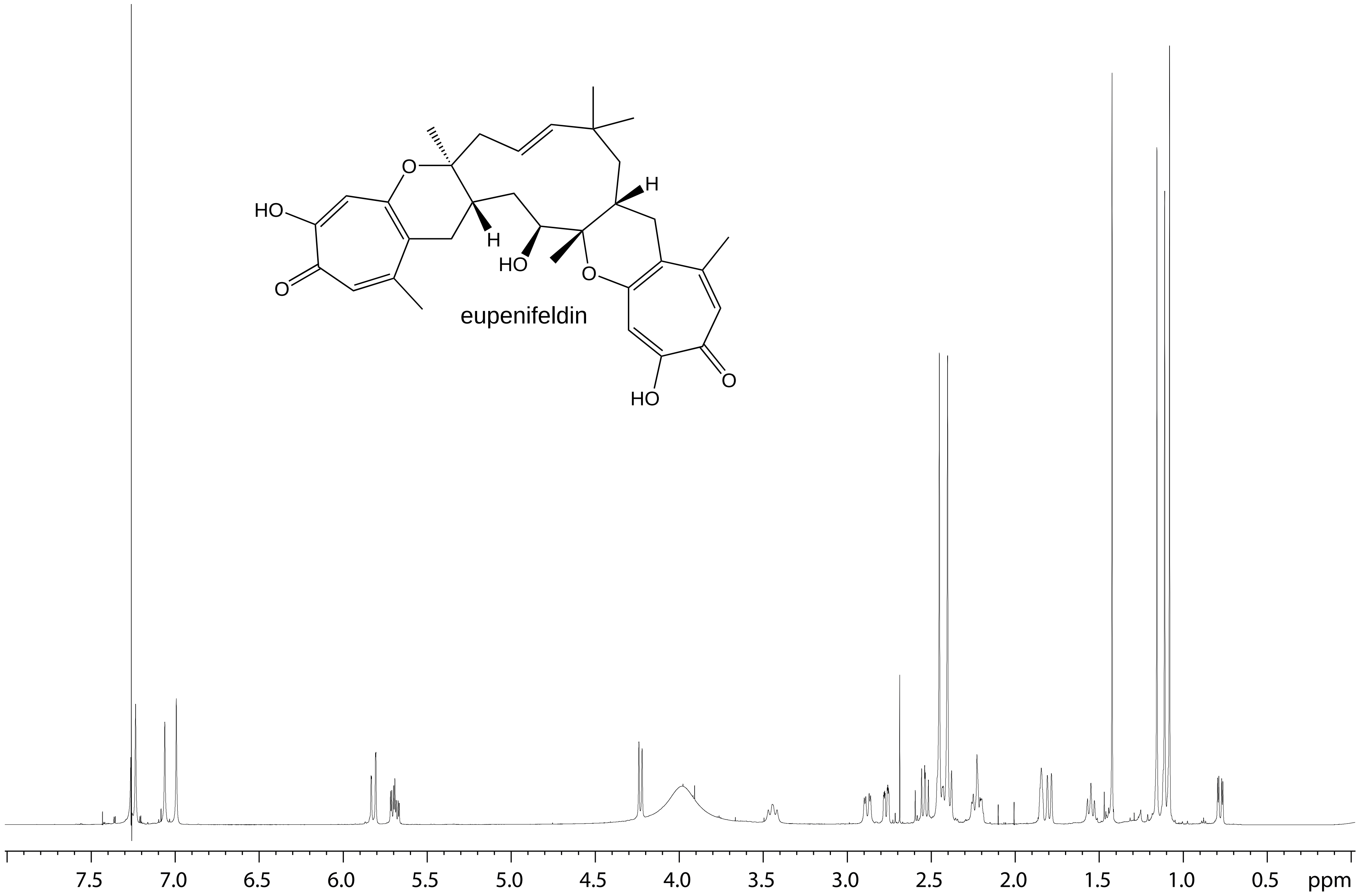

Figure S12. ^{1}H NMR spectrum (600 MHz, $CDCl_3$) of eupenifeldin

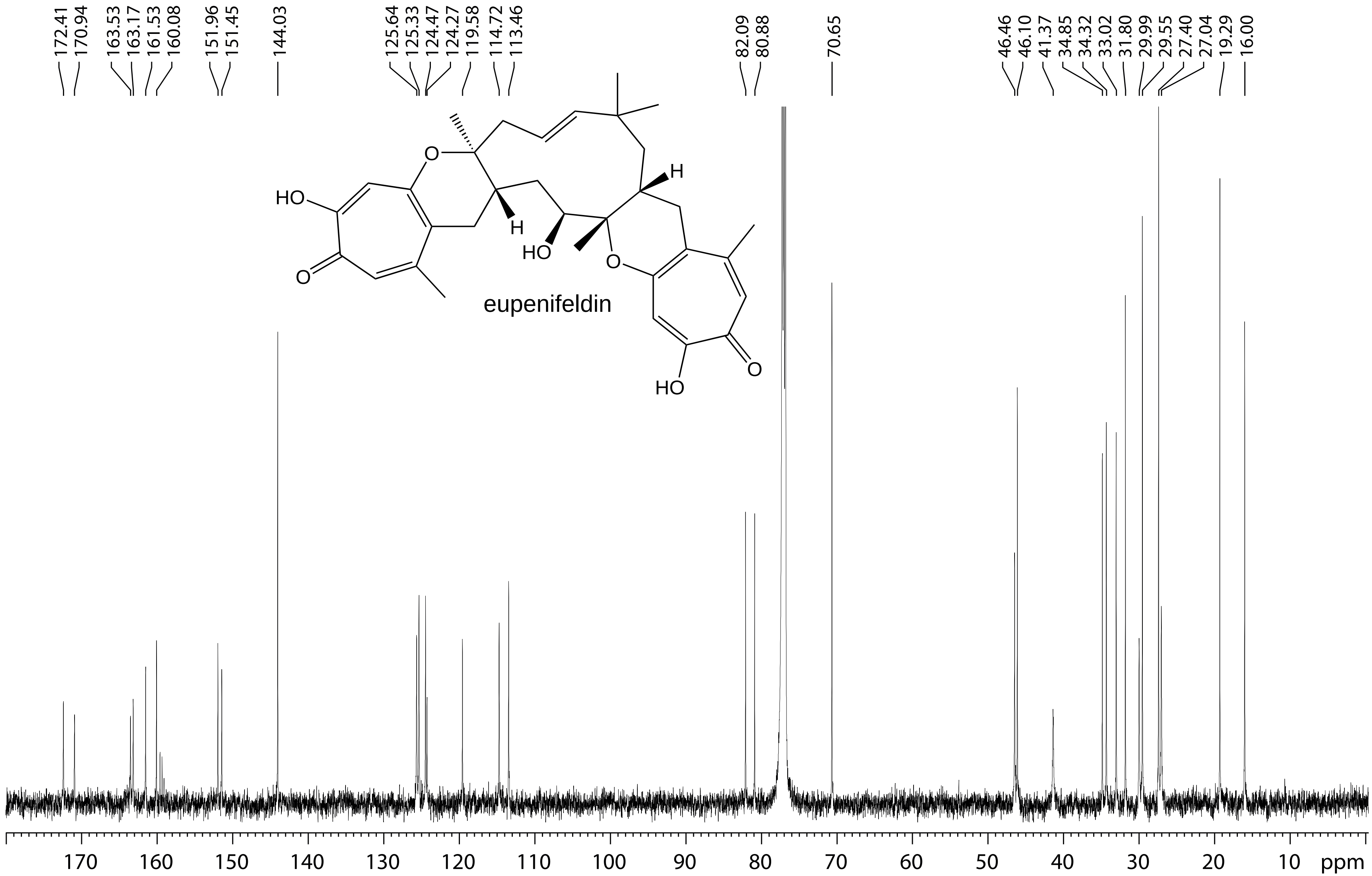

Figure S13. ^{13}C NMR spectrum (150 MHz, $CDCl_3$) of eupenifeldin

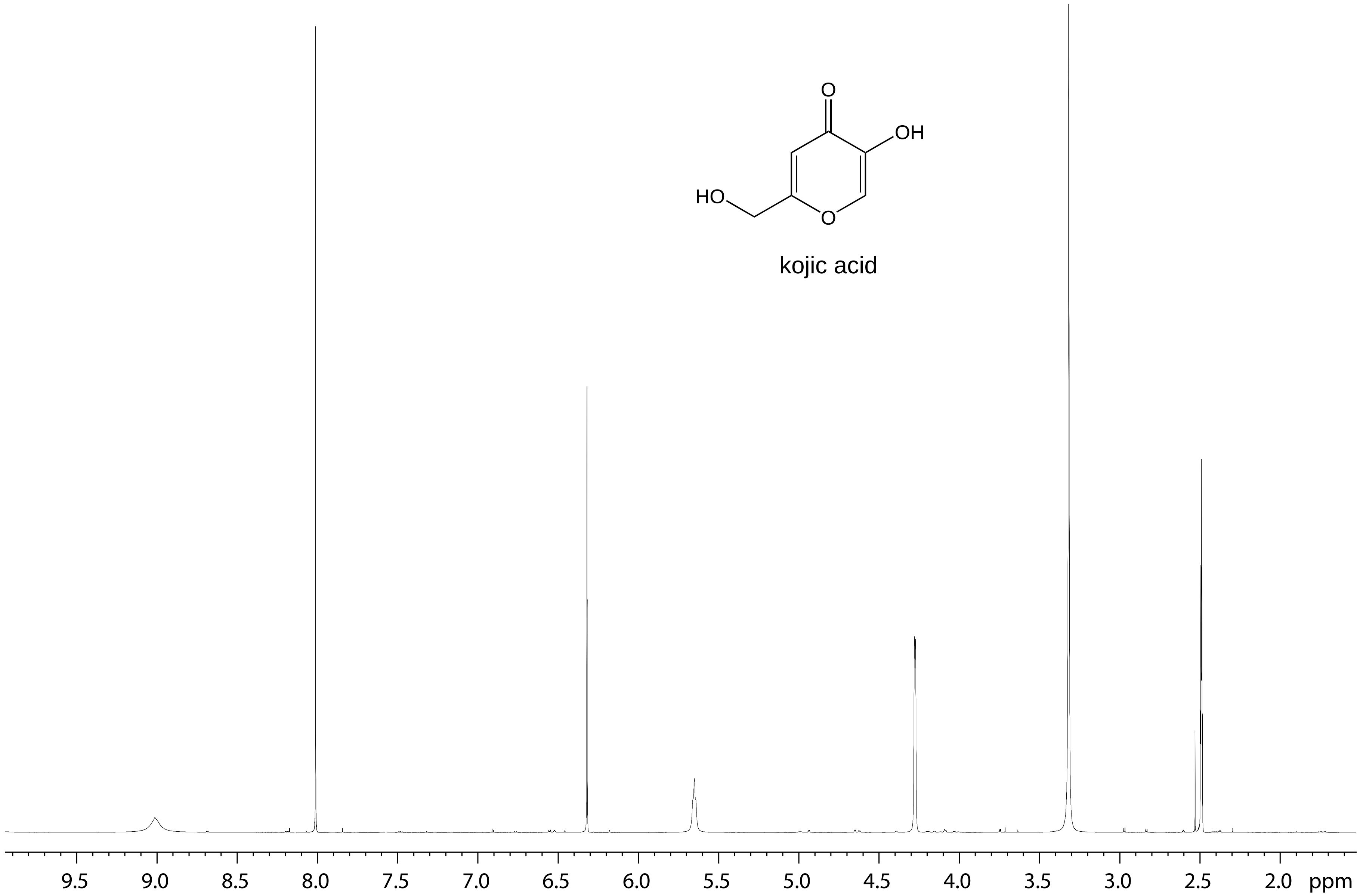

Figure S14. ^{1}H NMR spectrum (600 MHz, DMSO-d_6) of kojic acid

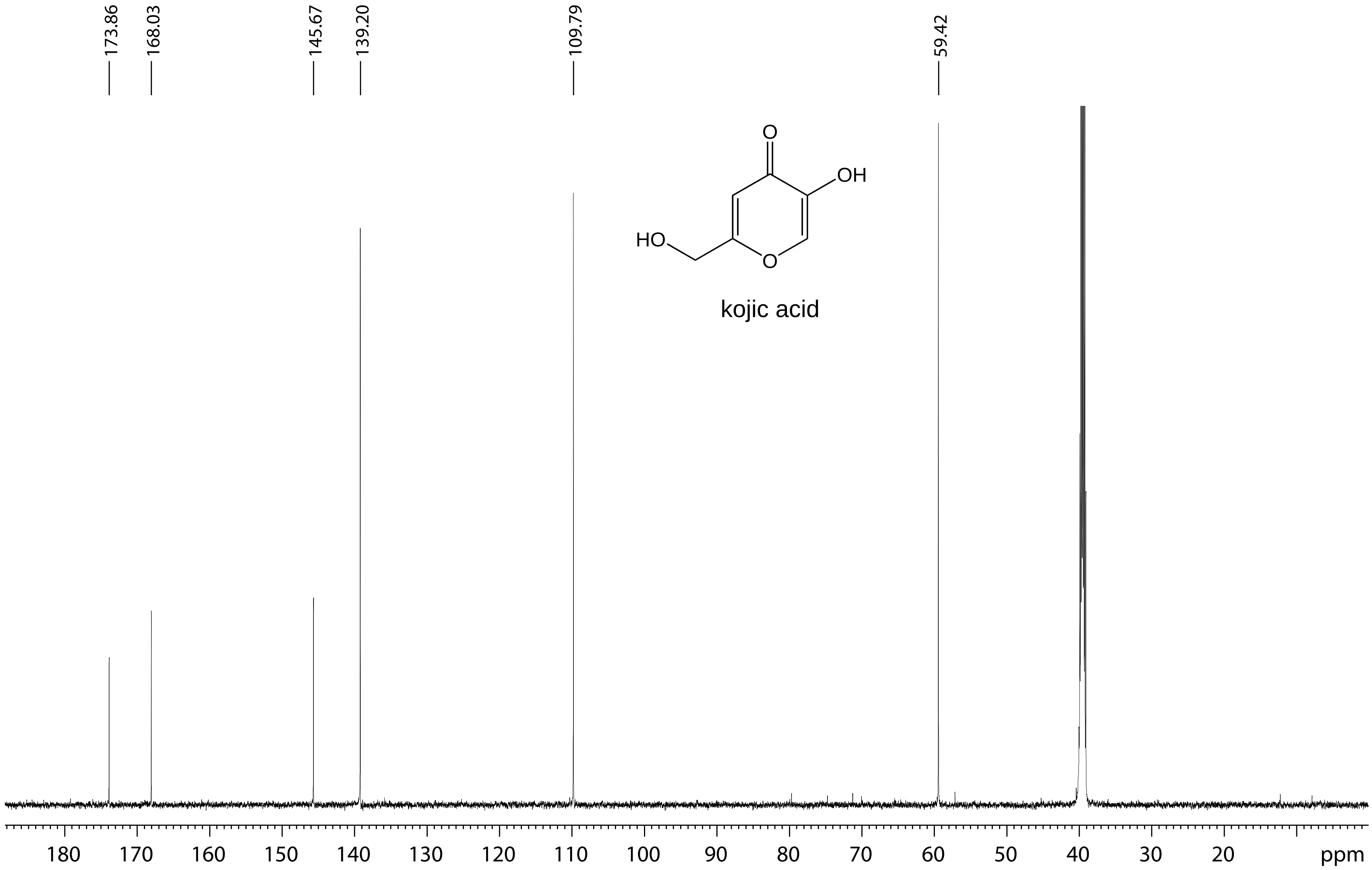

Figure S15. ^{13}C NMR spectrum (150 MHz, DMSO-d_6) of kojic acid

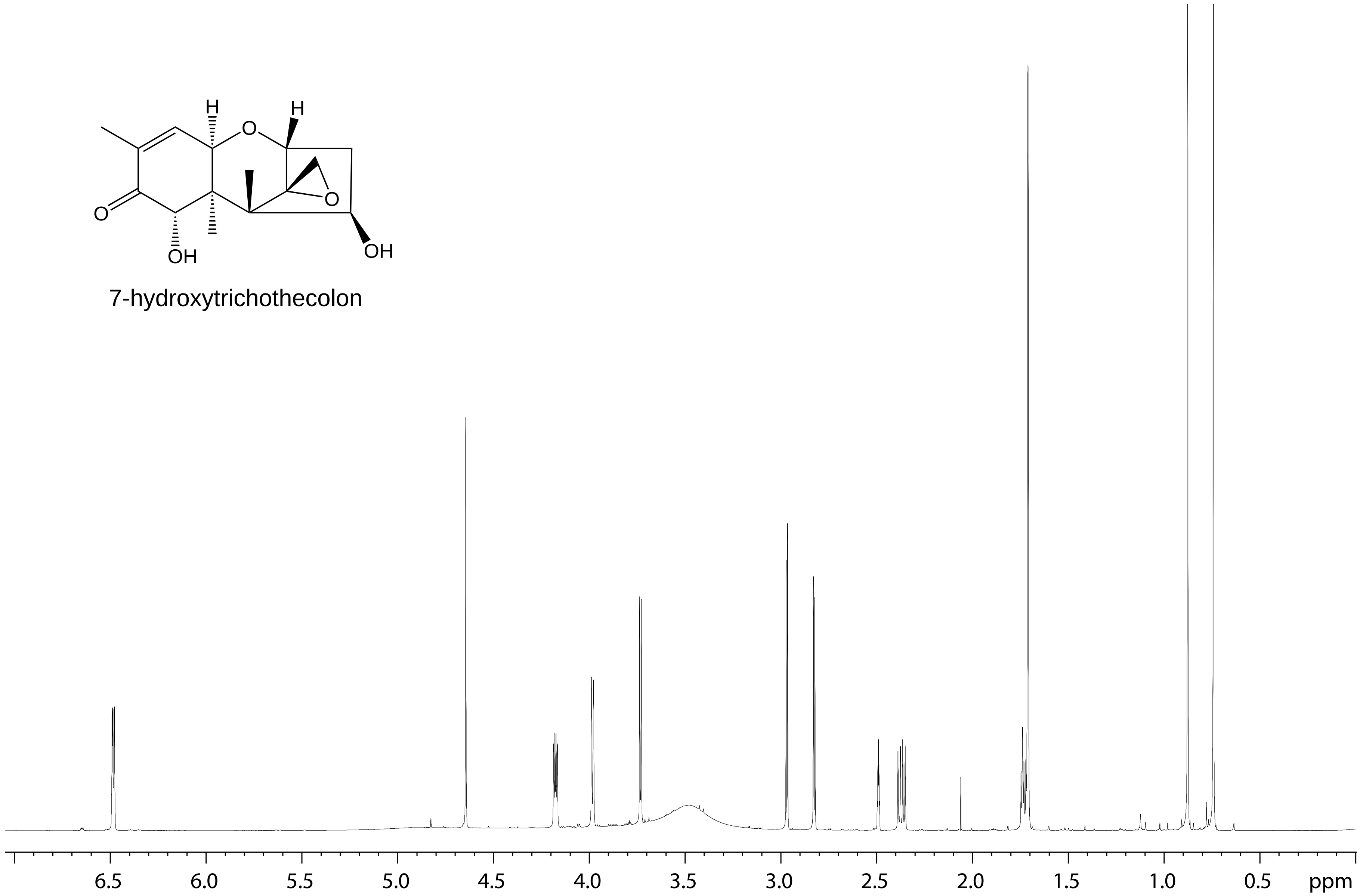

Figure S16. ^{1}H NMR spectrum (600 MHz, DMSO-d_6) of 7-hydroxytrichothecolon

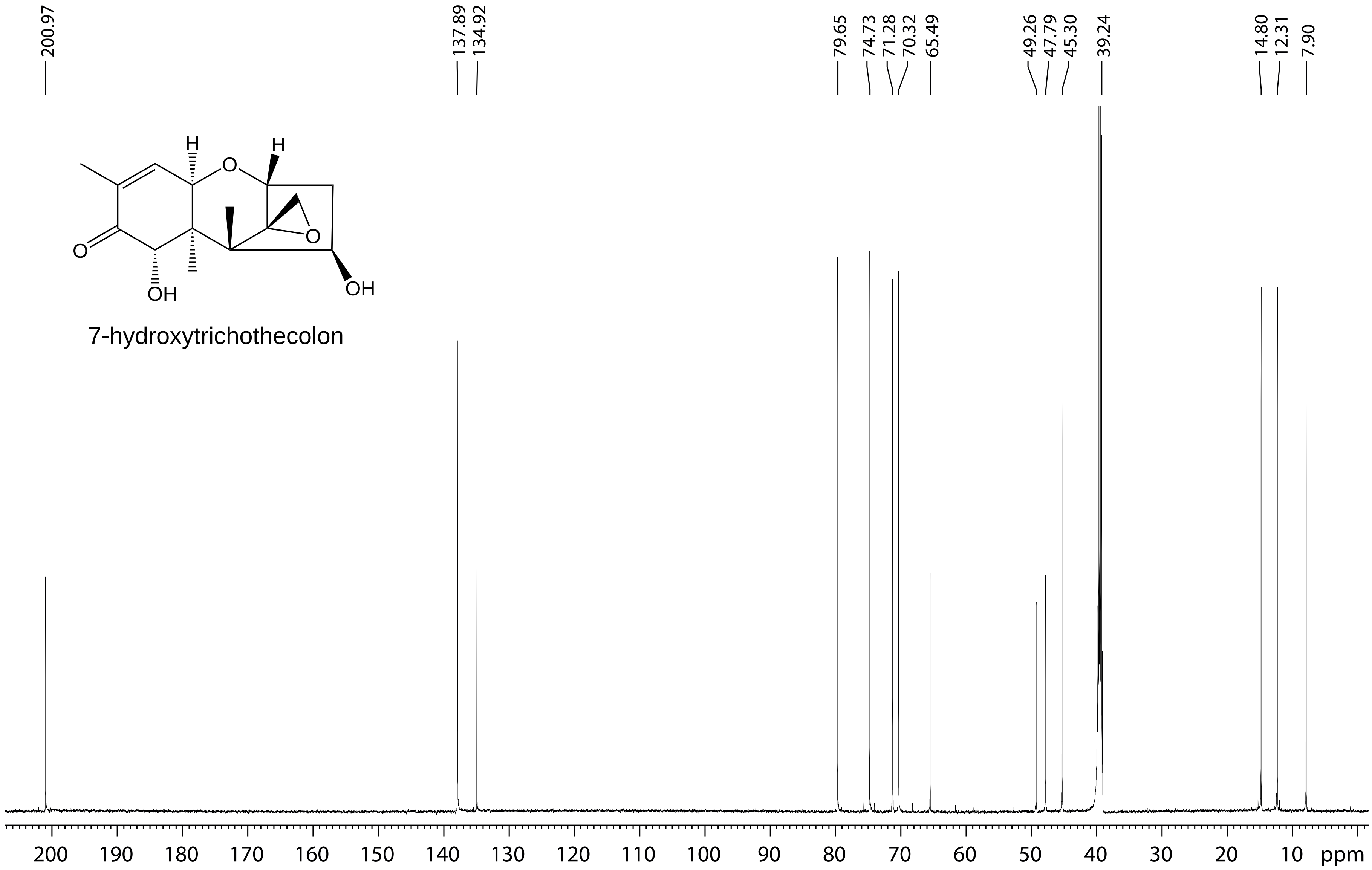

Figure S17. ^{13}C NMR spectrum (150 MHz, DMSO-d_6) of 7-hydroxytrichothecolon

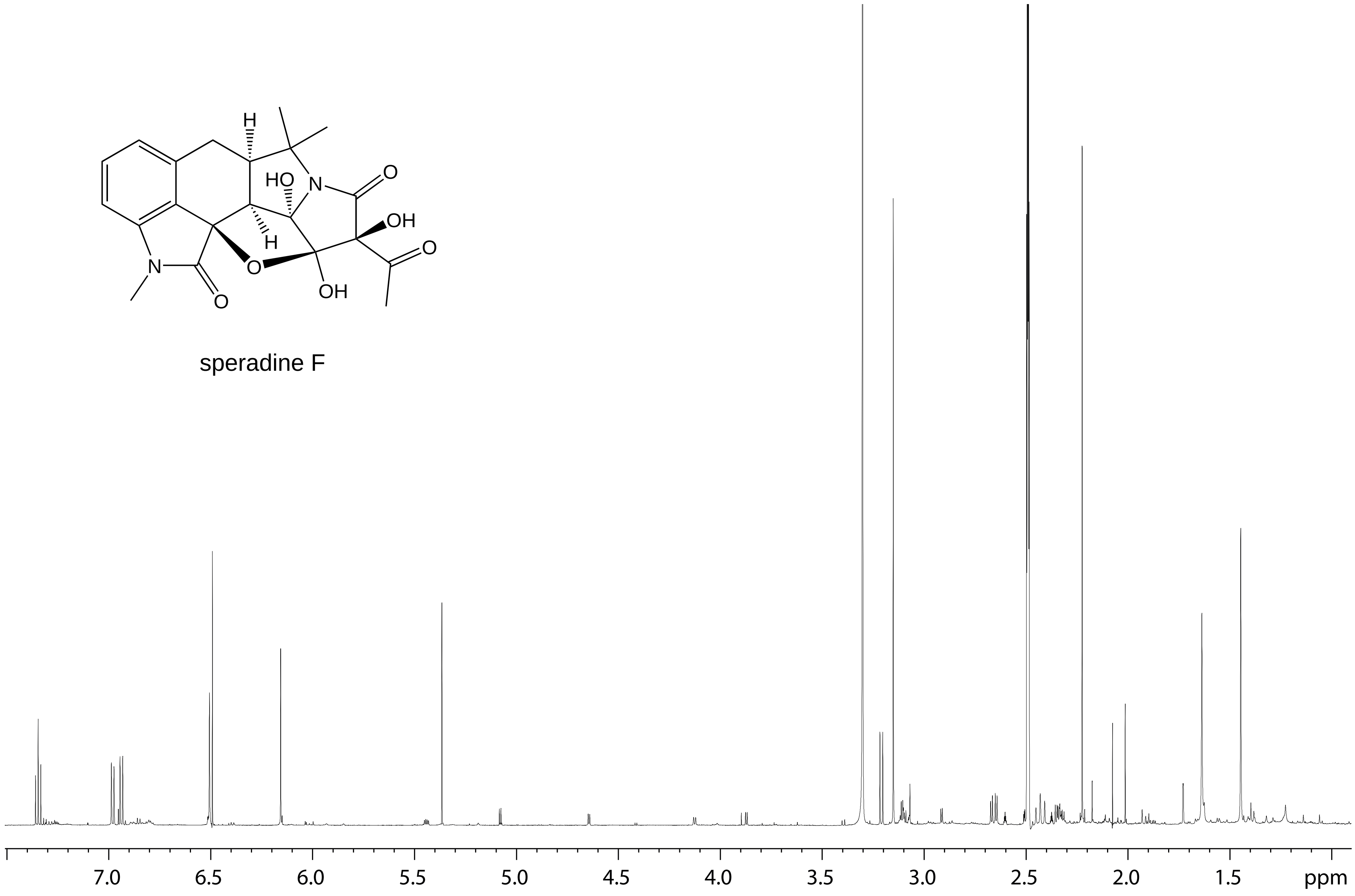

Figure S18. ^{1}H NMR spectrum (600 MHz, DMSO-d_6) of speradine F

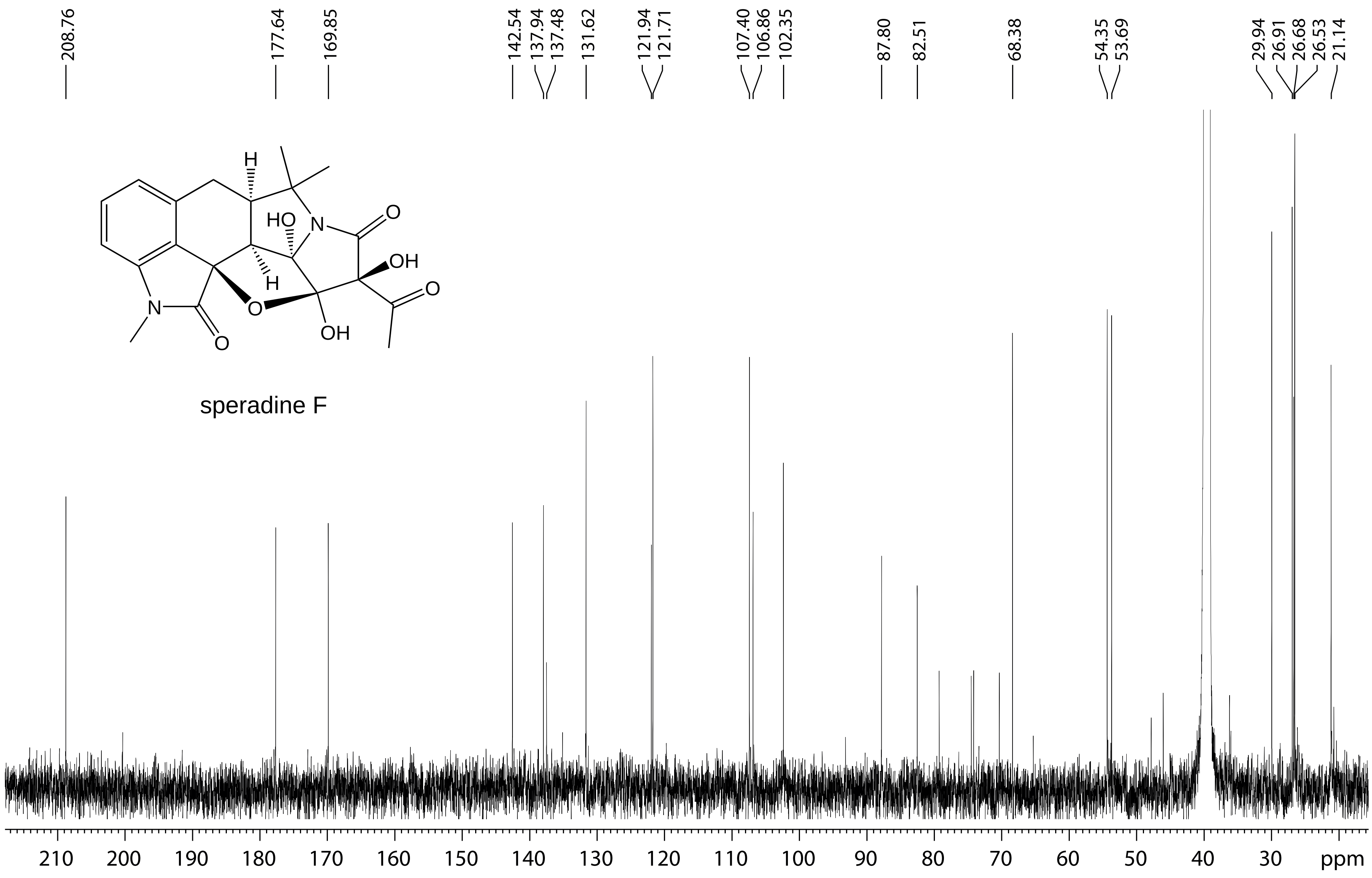

Figure S19. ^{13}C NMR spectrum (150 MHz, DMSO-d_6) of speradine F

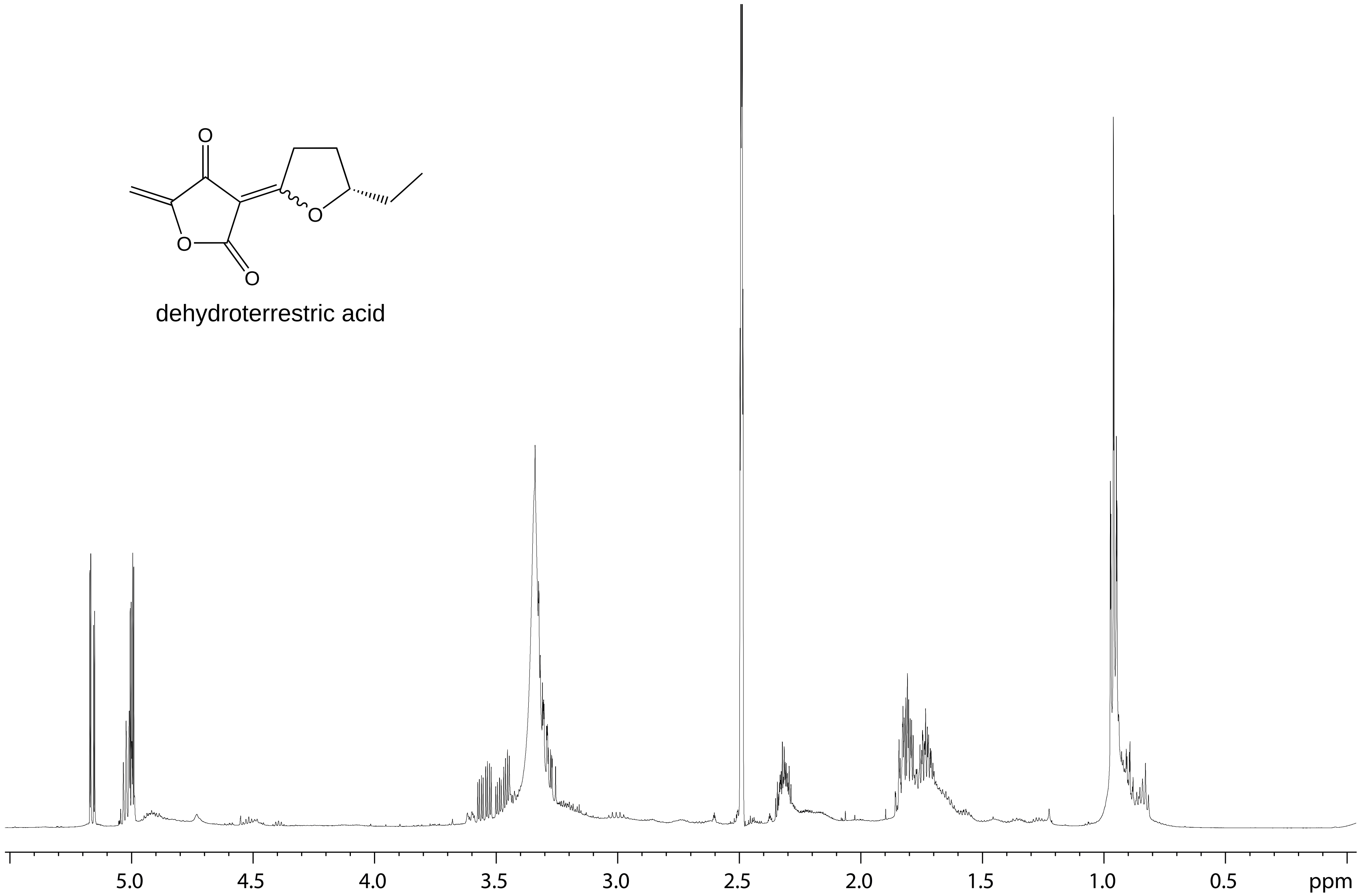

Figure S20. ^{1}H NMR spectrum (600 MHz, DMSO-d_6) of dehydroterrestric acid

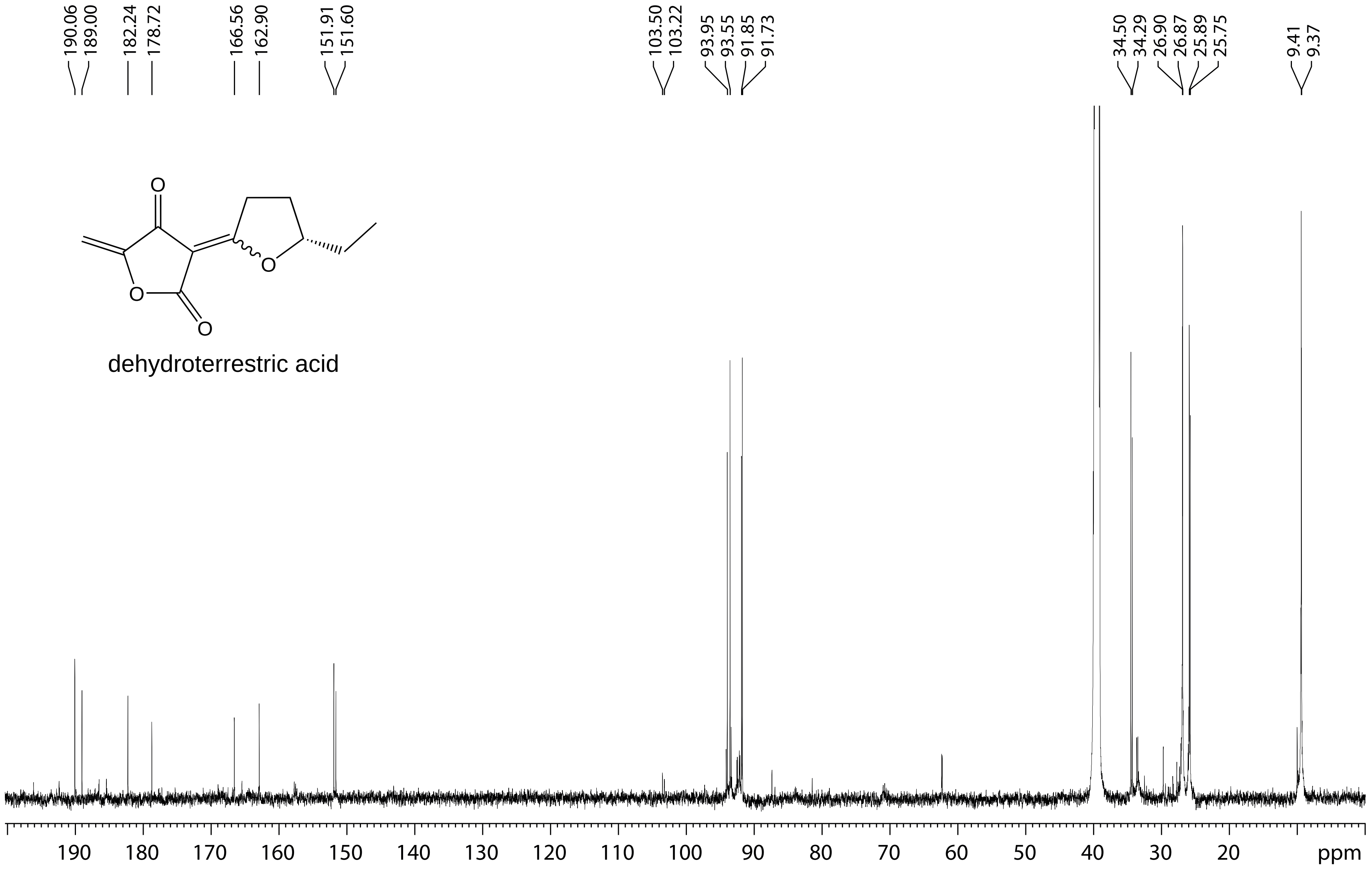

Figure S21. ^{13}C NMR spectrum (150 MHz, DMSO-d_6) of dehydroterrestric acid

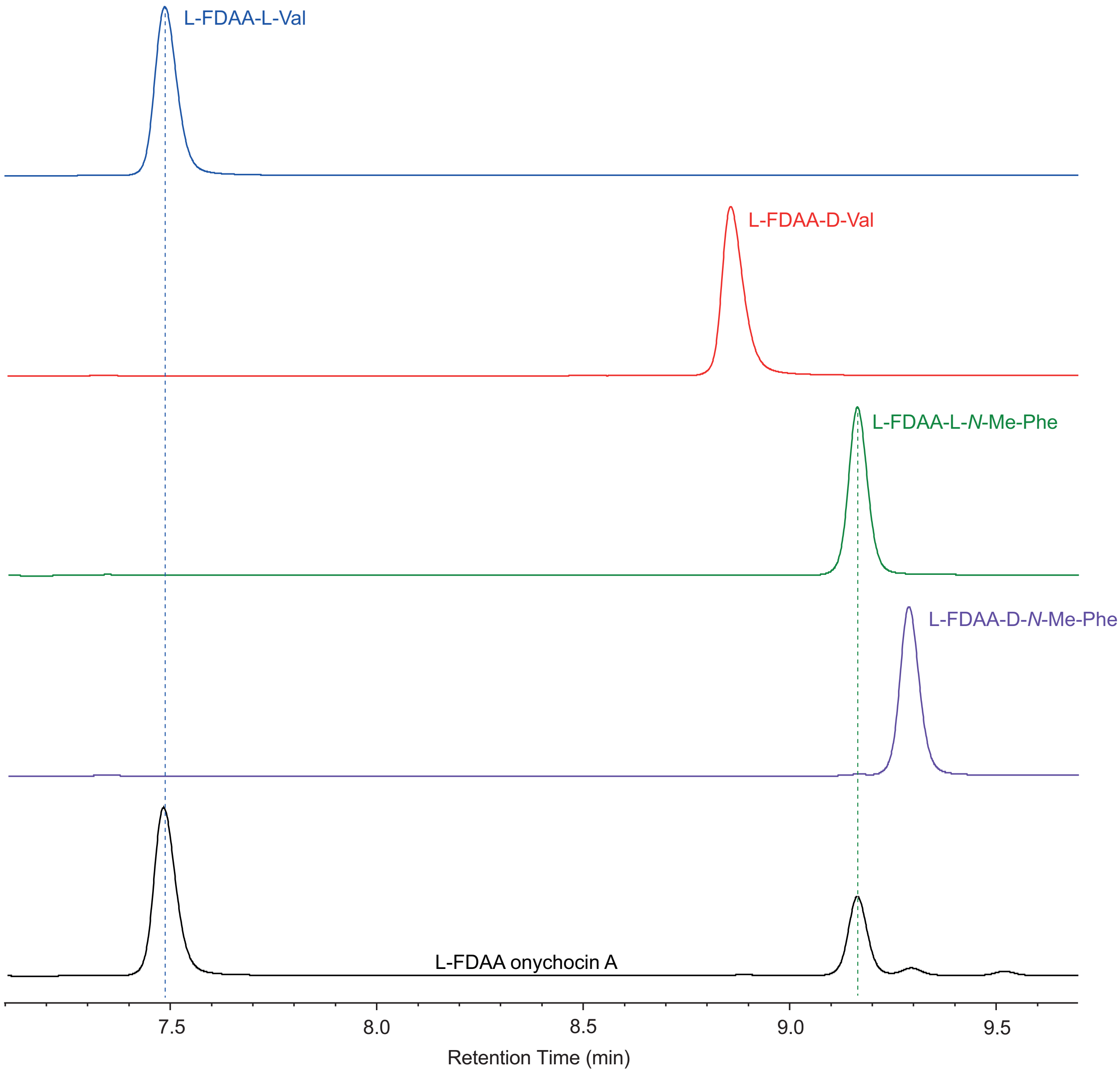

Figure S22. Marfey's Analysis of onychocin A

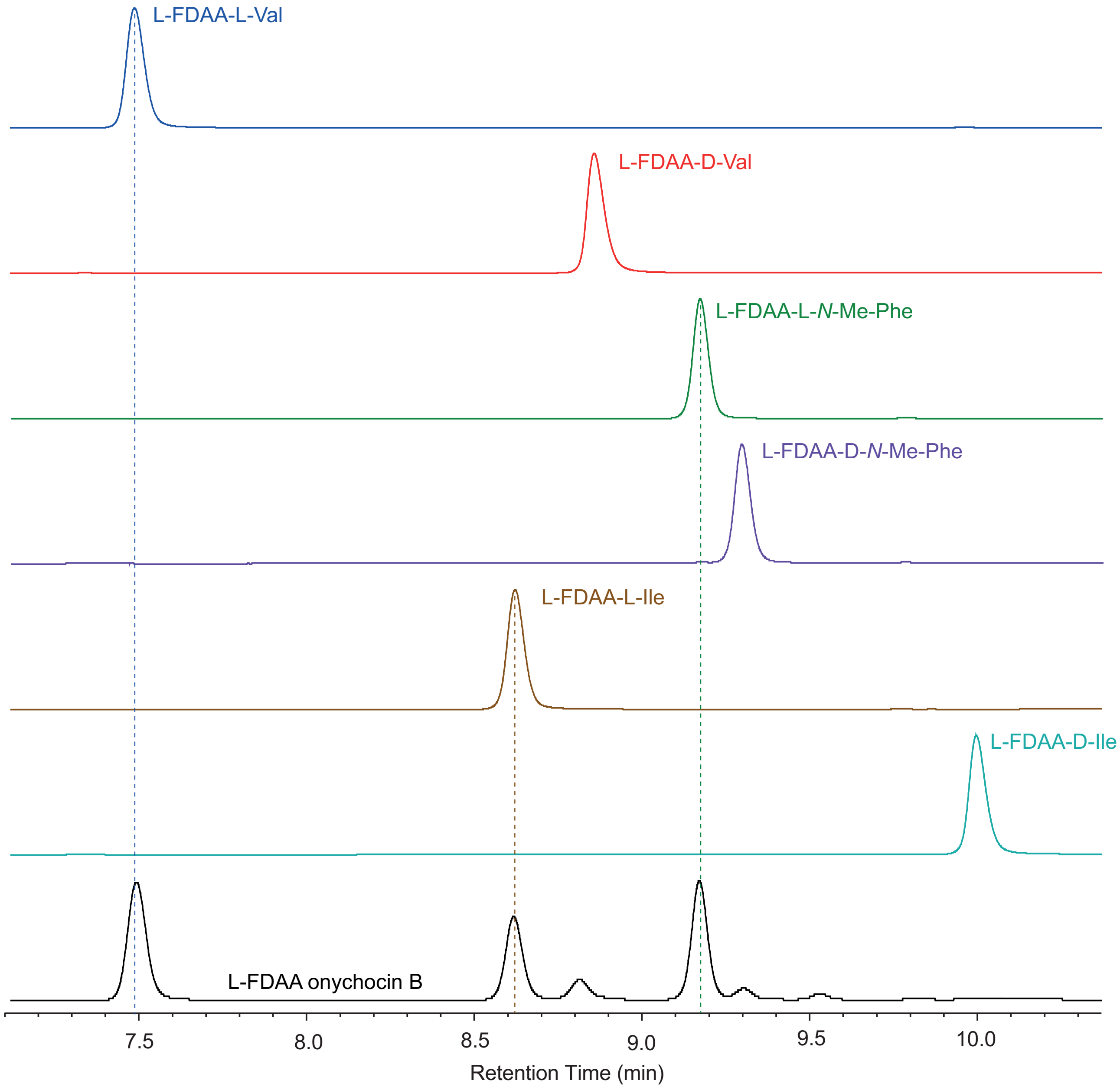

Figure S23. Marfey's Analysis of onychocin B

Table S11. LCMS analysis of *Aspergillus hancockii.* All eluted metabolites were identified by sequential peak numbers (1-69). Resolved peaks are annotated on the chromatogram trace, while the corresponding retention times, areas, UV-vis and MS spectra are listed in the table below. Molecular weights were tentatively assigned based on quasimolecular and adduct ion patterns. Where multiple solutions were observed, all tentative assignments are given. Where insufficient adducts were available, the molecular weights are left unassigned (?). Peaks detected in the positive-TIC but not detected by UV were annotated with an asterisk (*).

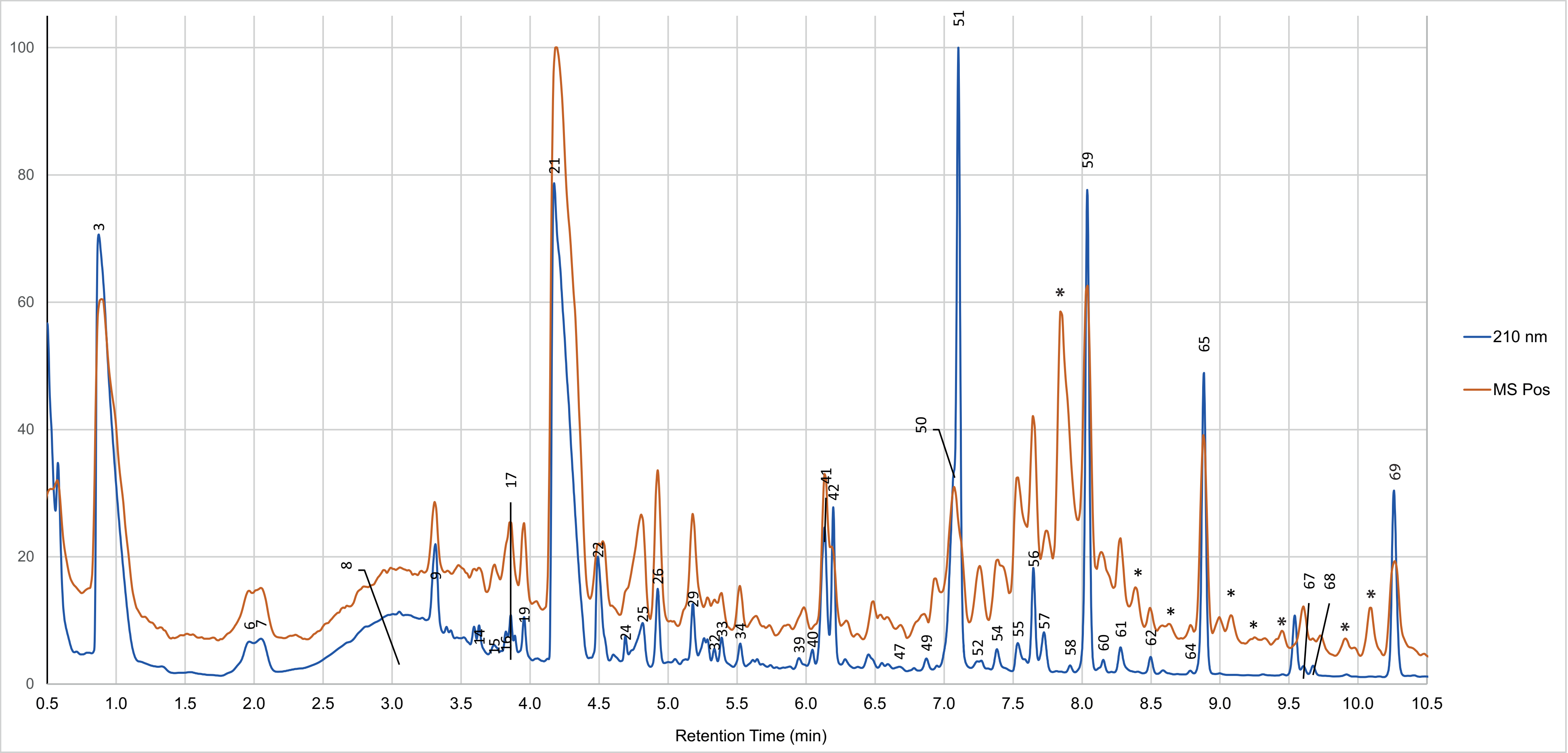

Peak	R.T.	Area	%Area	MW	UV Peaks	ID	UV-Vis	MS Positive	MS Negative
3	0.87	6351.0	18.39	342	206 (100)<br>230s (14)	Hancockiamide D	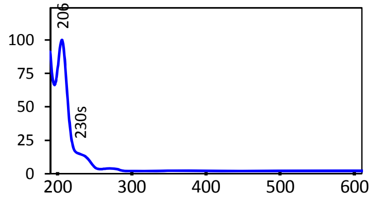	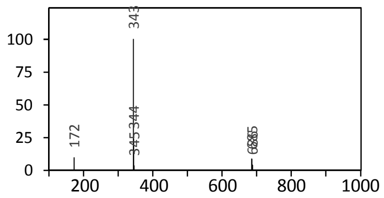	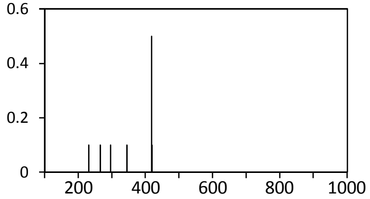
6	1.96	379.4	1.10	280	222 (100)<br>314 (25)	Hydroxytrichothecolon isomer	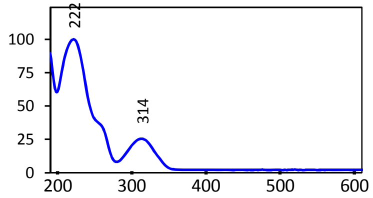	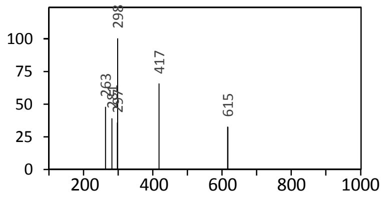	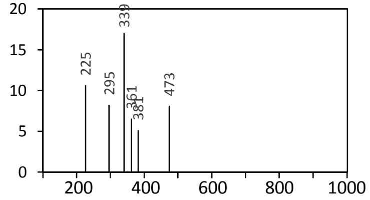
7	2.05	360.3	1.04	280	224 (100)<br>312 (22)	7-Hydroxytrichothecolon	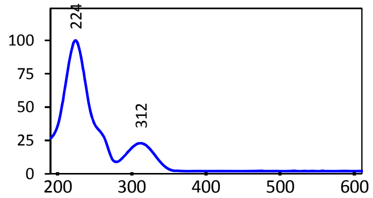	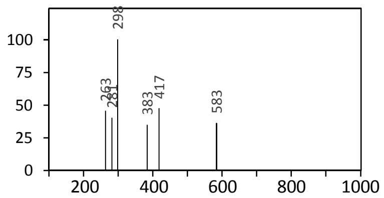	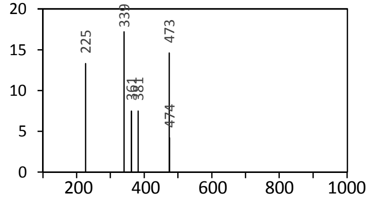
8	3.05	5153.6	14.93	226	246 (100)<br>292 (69)	Dehydroterrestric acid	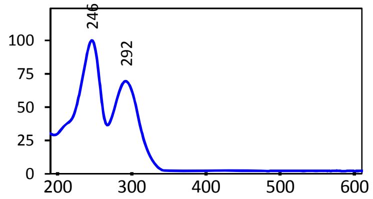	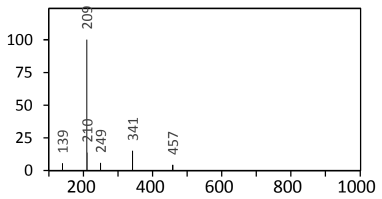	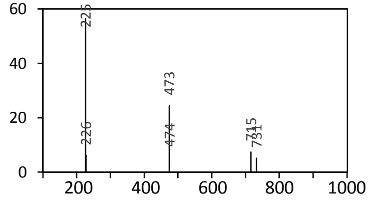
9	3.31	383.7	1.11	225	220 (53)<br>252 (100)<br>300 (81)	Unknown	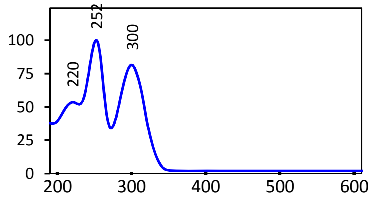	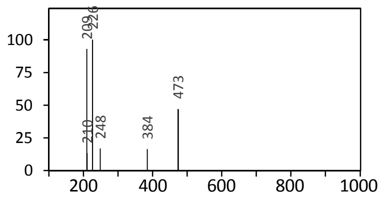	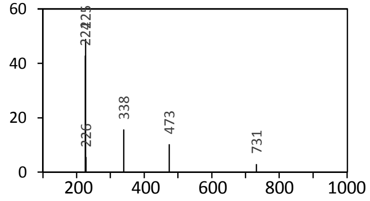
14	3.63	40.1	0.12	528<br>226	212 (100)<br>254 (39)<br>312 (42)	Unknown	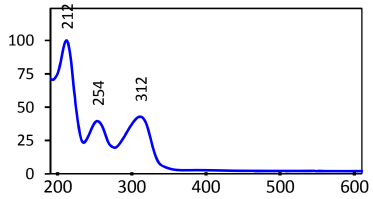	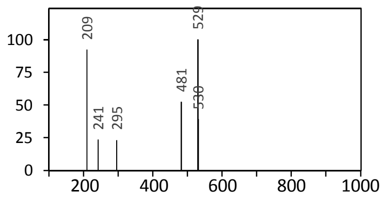	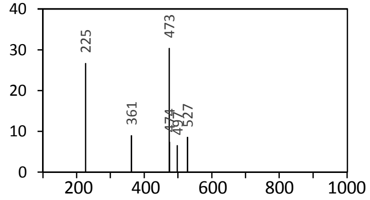

Peak	R.T.	Area	%Area	MW	UV Peaks	ID	UV-Vis	MS Positive	MS Negative
15	3.74	37.3	0.11	390<br>226	260 (12)<br>314 (11)	Speradine analogue	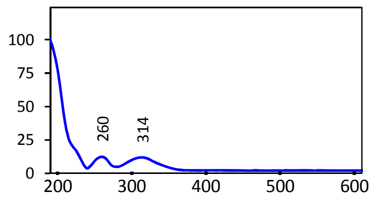	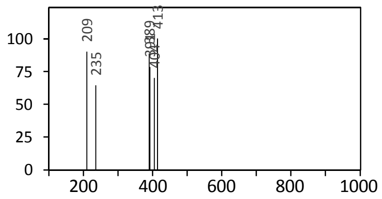	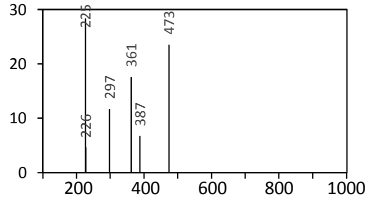
16	3.82	21.1	0.06	390	206 (75)<br>230 (45)<br>250s (27)<br>314 (21)	Unknown	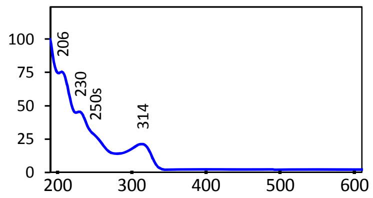	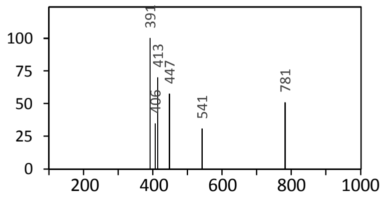	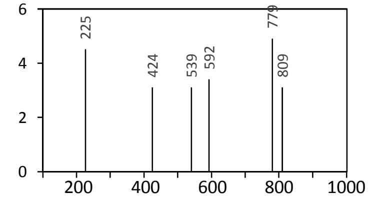
17	3.86	48.1	0.14	593	192 (100)<br>242 (63)<br>278 (51)	Unknown	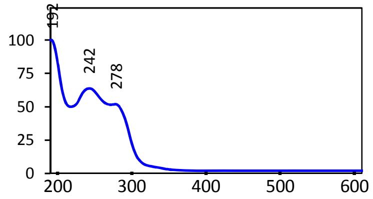	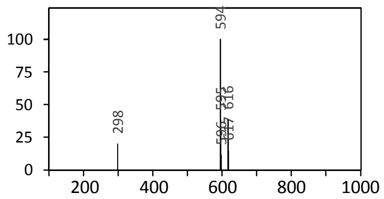	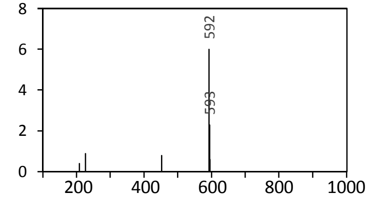
19	3.96	121.6	0.35	286	216 (91)<br>262 (21)<br>300 (14)	Unknown	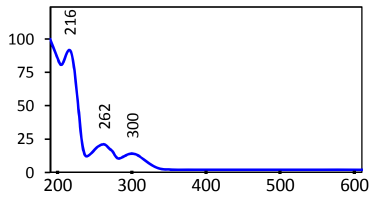	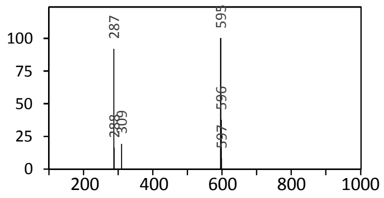	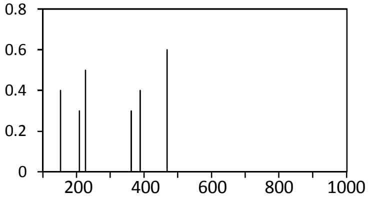
21	4.17	7016.5	20.32	472	206 (97)<br>240s (19)<br>280 (32)	Hancockiamide A	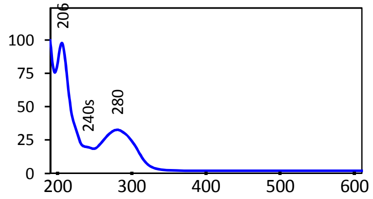	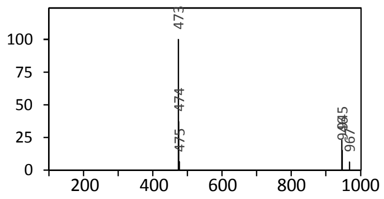	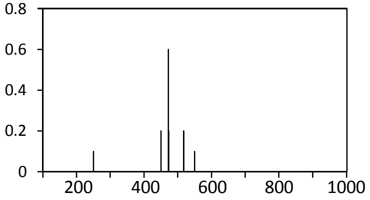
22	4.49	538.5	1.56	546	208 (100)<br>242 (22)<br>270s (22)<br>282 (25)	Hancockiamide E	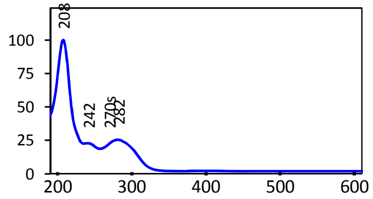	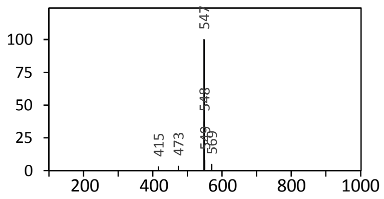	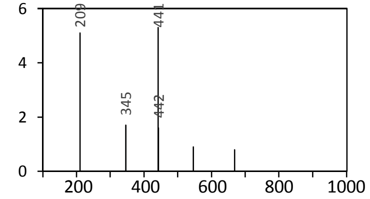

Peak	R.T.	Area	%Area	MW	UV Peaks	ID	UV-Vis	MS Positive	MS Negative
24	4.69	48.5	0.14	573	206 (93)<br>278 (35)	Hancockiamide analogue	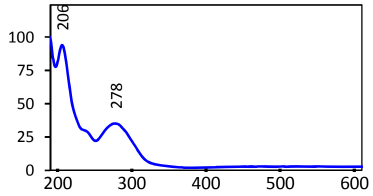	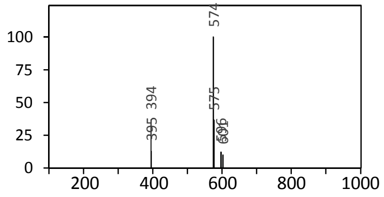	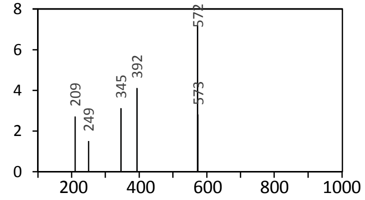
25	4.82	274.0	0.79	414	218 (95)<br>262 (12)<br>310 (8)	Speridine analogue	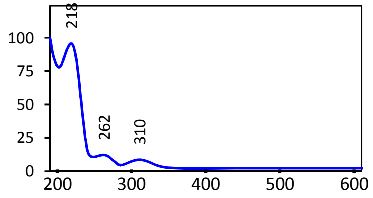	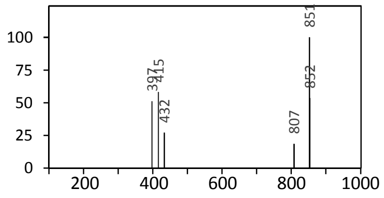	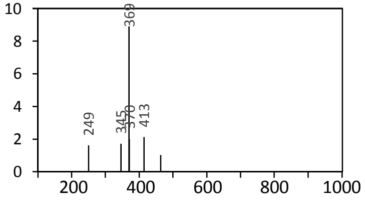
26	4.92	275.3	0.80	414	222 (89)<br>264 (13)<br>302 (10)	Speradine F	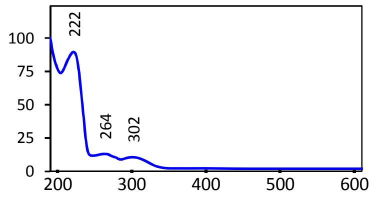	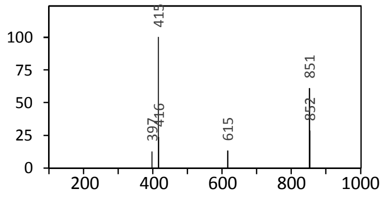	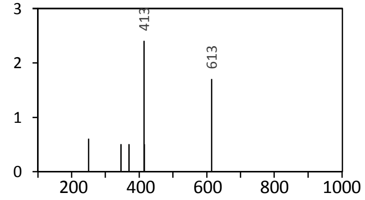
29	5.18	177.8	0.52	382	218 (85)<br>276 (50)	Unknown	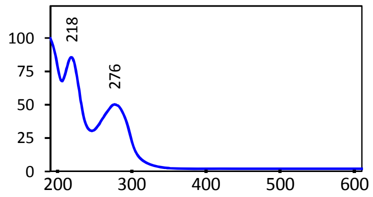	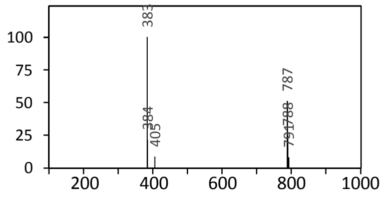	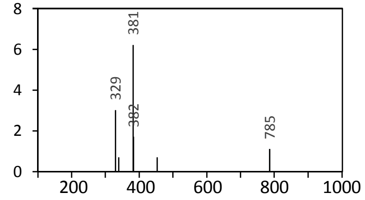
32	5.34	30.6	0.09	393	194 (100)<br>220 (81)<br>262 (13)<br>296 (20)	Fumitremorgin analogue	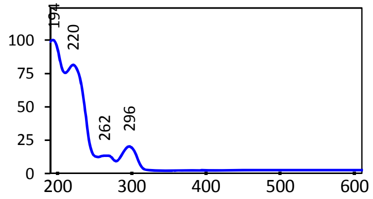	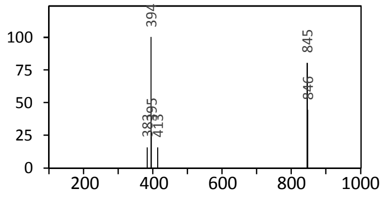	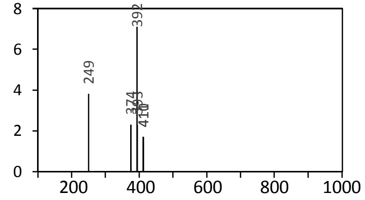
33	5.39	89.0	0.26	378	198 (91)<br>220 (100)<br>264 (48)<br>334 (7)<br>350 (9)	Unknown	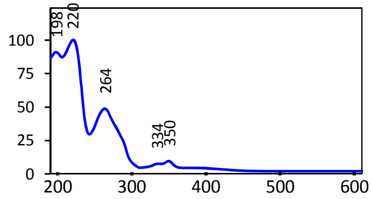	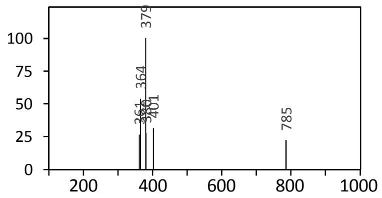	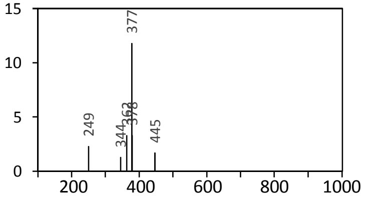

Peak	R.T.	Area	%Area	MW	UV Peaks	ID	UV-Vis	MS Positive	MS Negative
34	5.52	85.0	0.25	382	218 (76)<br>274 (33)	Unknown	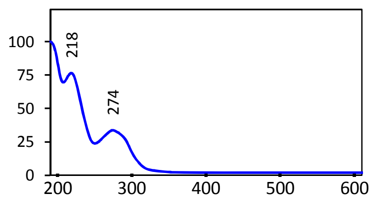	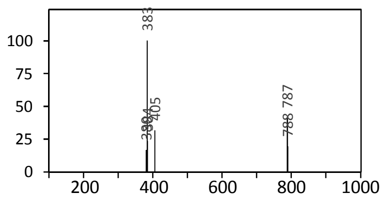	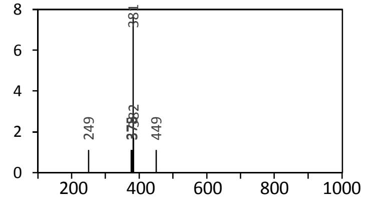
39	5.95	41.7	0.12	366	202 (93)<br>224 (100)<br>266s (17)<br>280s (18)<br>294 (21)	Fumitremorgin analogue	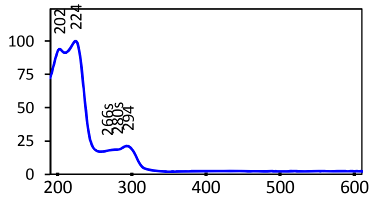	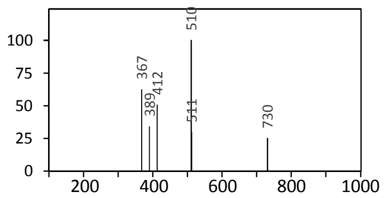	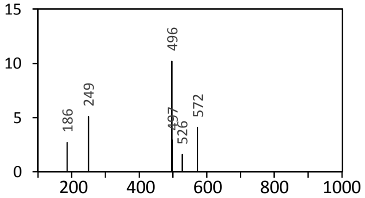
40	6.05	44.6	0.13	474	192 (100)<br>224 (25)	Cyclic peptide	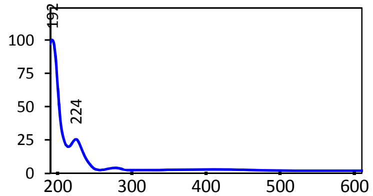	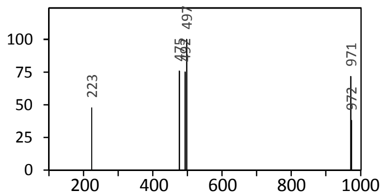	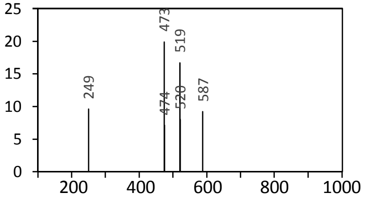
41	6.13	737.8	2.14	384	212 (87)<br>254 (41)<br>282 (40)	Hancockiamide analogue	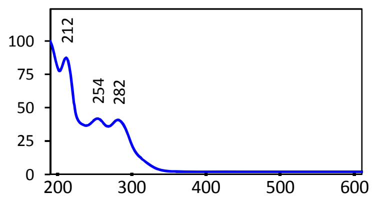	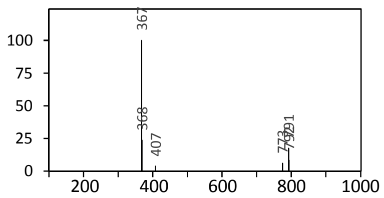	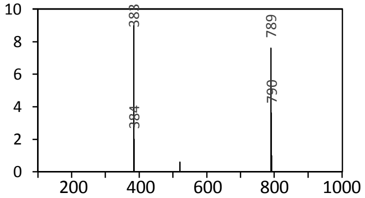
42	6.20	523.5	1.52	514	206 (96)<br>246s (16)<br>280 (33)	Hancockiamide B	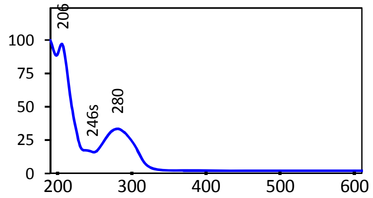	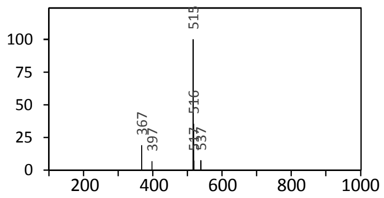	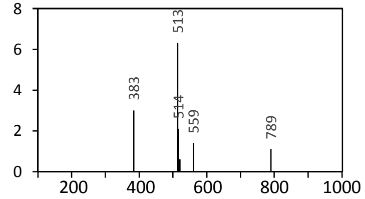
47	6.68	17.6	0.05	502	< 190	Unknown	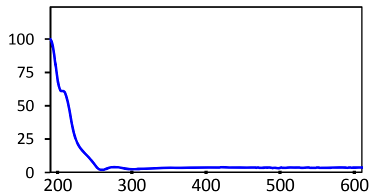	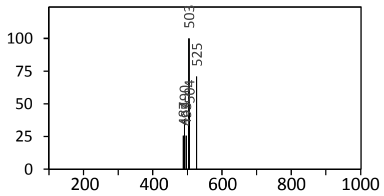	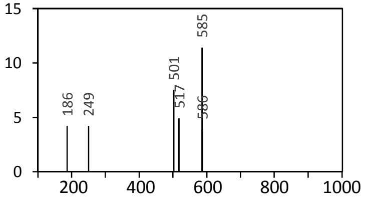

Peak	R.T.	Area	%Area	MW	UV Peaks	ID	UV-Vis	MS Positive	MS Negative
49	6.87	46.2	0.13	467	194 (100)<br>256 (64)	Unknown	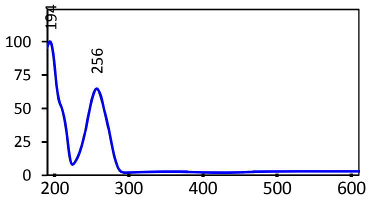	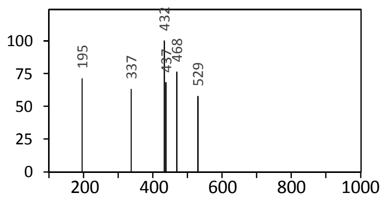	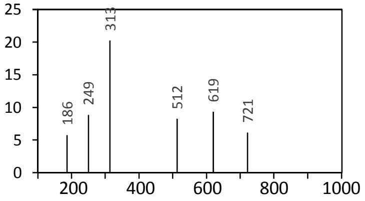
50	7.08	920.1	2.66	512	212 (80)<br>276 (34)	Hancockiamide F	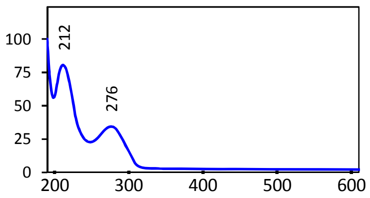	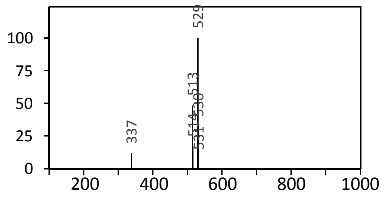	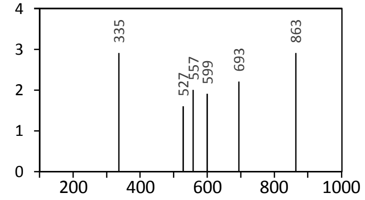
51	7.10	2249.5	6.52	512	208 (95)<br>250 (19)<br>282 (31)	Hanockiamide C	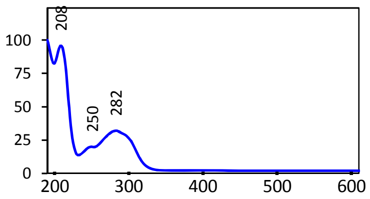	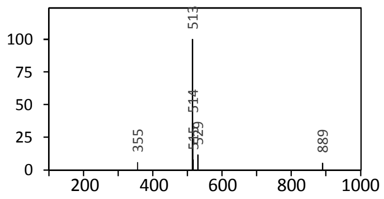	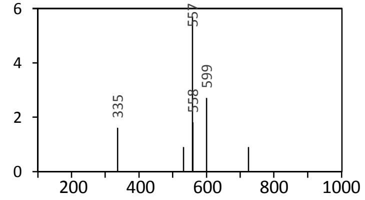
52	7.24	27.8	0.08	477	228 (100)<br>274 (15)<br>294 (17)	Fumitremorgin analogue	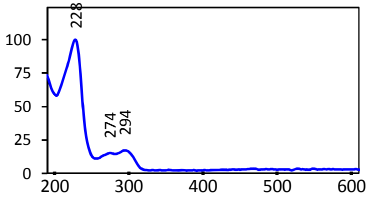	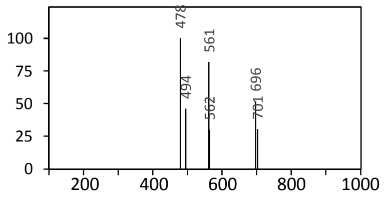	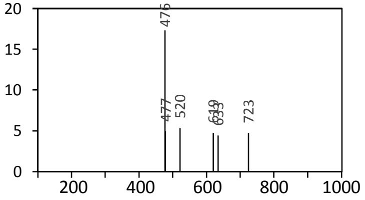
54	7.38	102.3	0.30	477	< 190	Unknown	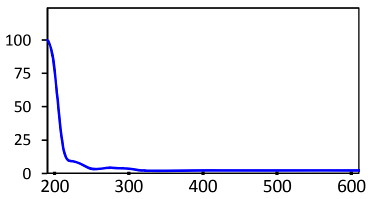	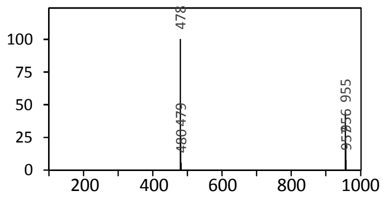	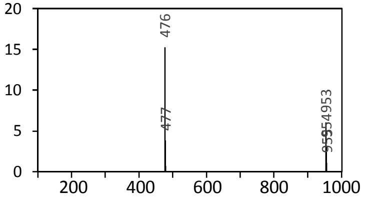
55	7.53	128.8	0.37	519	228 (6)	Unknown	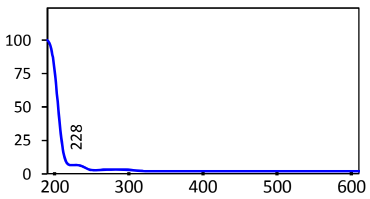	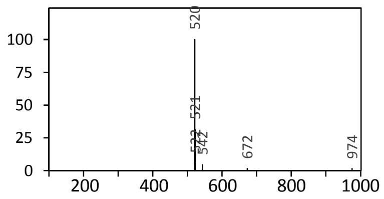	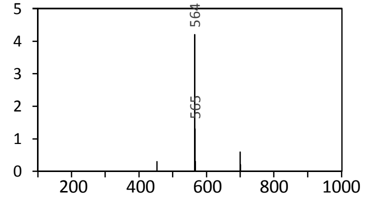

Peak	R.T.	Area	%Area	MW	UV Peaks	ID	UV-Vis	MS Positive	MS Negative
56	7.65	306.2	0.89	520	206s (30)	Onychocin A	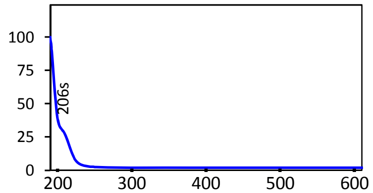	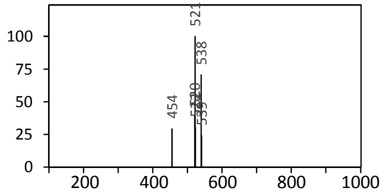	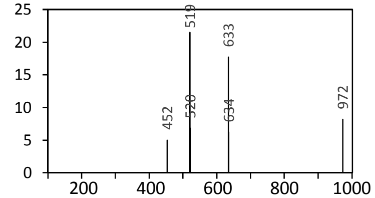
57	7.72	136.9	0.40	495<br>519	198 (100)<br>228 (91)<br>272 (19)<br>294 (15)	Fumitremorgin analogue	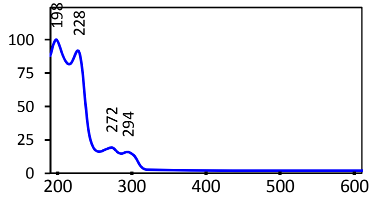	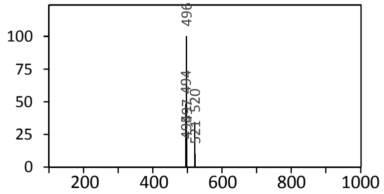	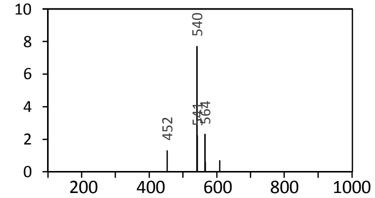
58	7.91	23.0	0.07	495	224 (36)<br>276 (9)<br>296 (9)	Fumitremorgin analogue	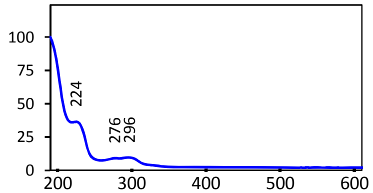	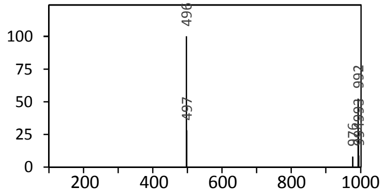	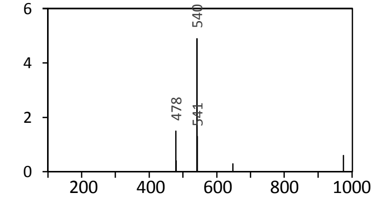
59	8.04	1869.9	5.42	534	192 (100)<br>206s (40)	Onychocin B	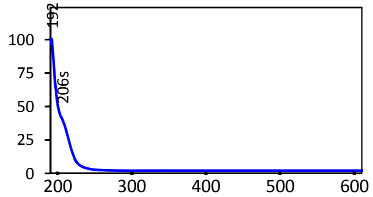	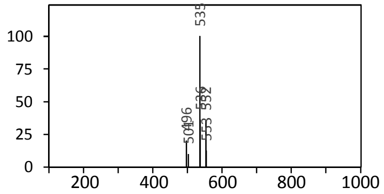	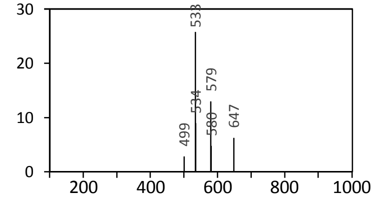
60	8.15	44.1	0.13	521*<br>495	208 (92)<br>252s (25)<br>282 (41)	Hancockiamide analogue	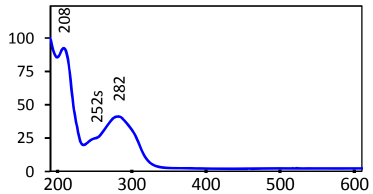	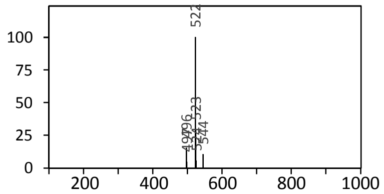	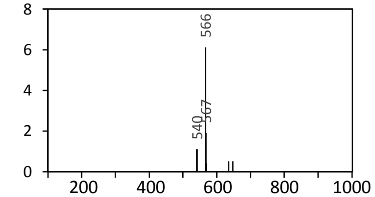
61	8.28	121.6	0.35	380*<br>521<br>782	194 (77)<br>230 (100)<br>280 (60)	Unknown	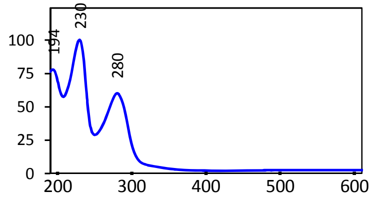	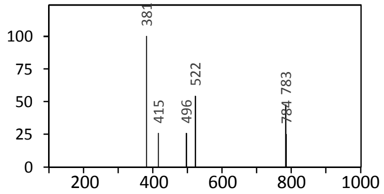	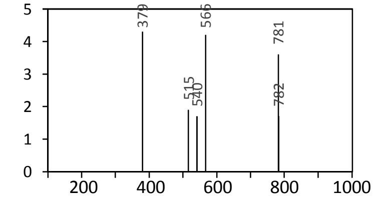

Peak	R.T.	Area	%Area	MW	UV Peaks	ID	UV-Vis	MS Positive	MS Negative
62	8.50	64.2	0.19	495<br>521	226 (75)<br>276 (27)<br>288s (26)	Fumitremorgin	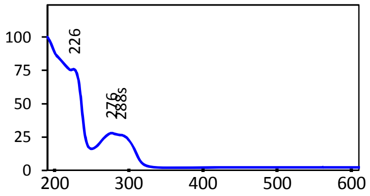	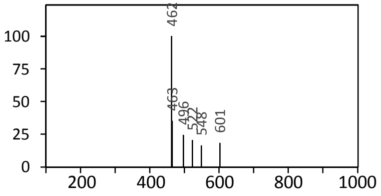	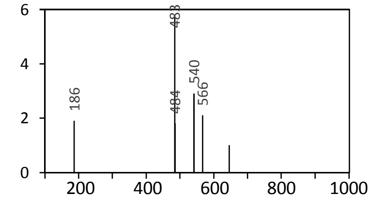
64	8.78	9.8	0.03	?	226 (55)<br>278 (12)<br>294 (12)	Fumitremorgin analogue	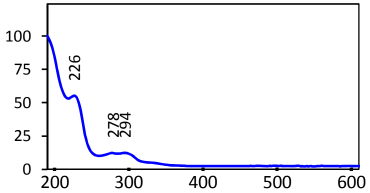	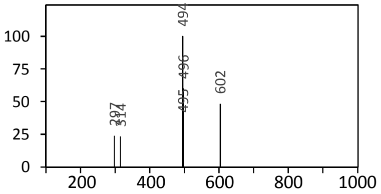	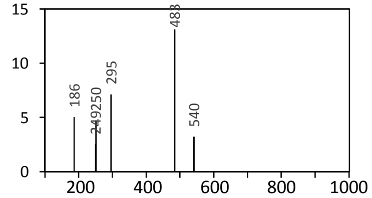
65	8.88	1193.2	3.46	579	196 (100)<br>226 (68)<br>276 (14)<br>294 (14)	Fumitremorgin A	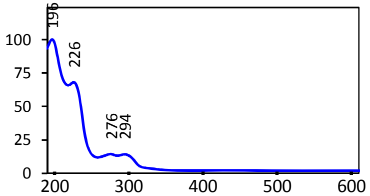	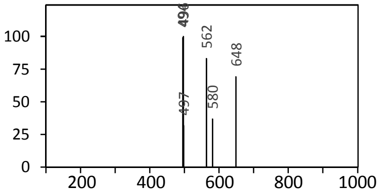	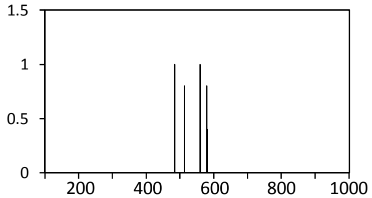
67	9.60	16.2	0.05	354	< 190	Fatty Acid	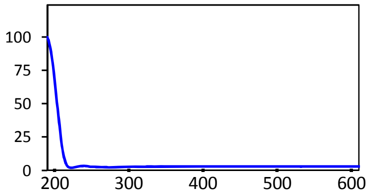	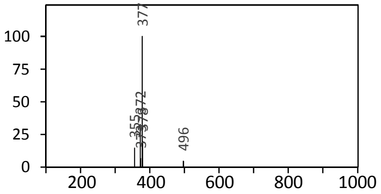	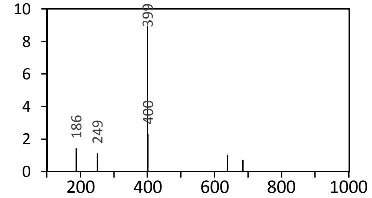
68	9.67	32.8	0.09	278	< 190	Fatty Acid	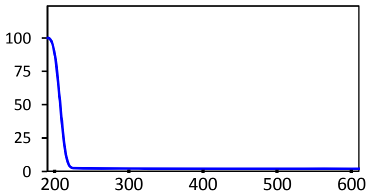	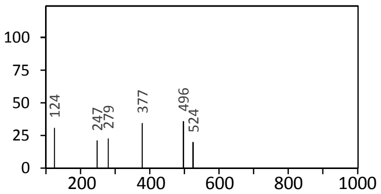	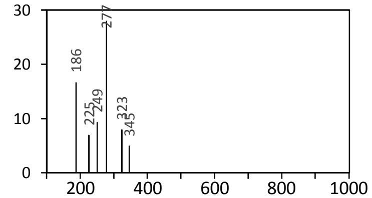
69	10.26	838.4	2.43	280	192 (100)	Linoleic acid	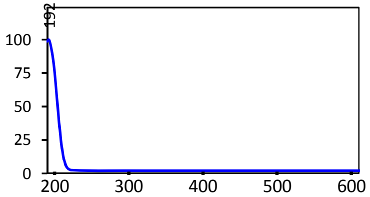	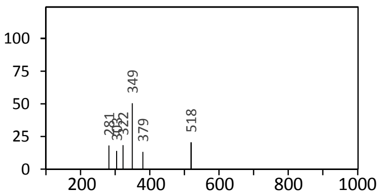	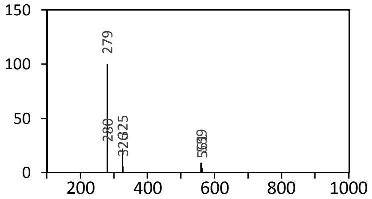
